# Relative risks of adverse events among older adults receiving opioids versus NSAIDs after hospital discharge: A nationwide cohort study

**DOI:** 10.1371/journal.pmed.1003804

**Published:** 2021-09-27

**Authors:** Shoshana J. Herzig, Timothy S. Anderson, Yoojin Jung, Long Ngo, Dae H. Kim, Ellen P. McCarthy

**Affiliations:** 1 Division of General Medicine, Department of Medicine, Beth Israel Deaconess Medical Center, Boston, Massachusetts, United States of America; 2 Harvard Medical School, Boston, Massachusetts, United States of America; 3 Division of Gerontology, Department of Medicine, Beth Israel Deaconess Medical Center, Boston, Massachusetts, United States of America; 4 Hinda and Arthur Marcus Institute for Aging Research, Hebrew SeniorLife, Boston, Massachusetts, United States of America; Harvard Medical School, UNITED STATES

## Abstract

**Background:**

Although analgesics are initiated on hospital discharge in millions of adults each year, studies quantifying the risks of opioids and nonsteroidal anti-inflammatory drugs (NSAIDs) among older adults during this transition are limited. We sought to determine the incidence and risk of post-discharge adverse events among older adults with an opioid claim in the week after hospital discharge, compared to those with NSAID claims only.

**Methods and findings:**

We performed a retrospective cohort study using a national sample of Medicare beneficiaries age 65 and older, hospitalized in United States hospitals in 2016. We excluded beneficiaries admitted from or discharged to a facility. We derived a propensity score that included over 100 factors potentially related to the choice of analgesic, including demographics, diagnoses, surgeries, and medication coadministrations. Using 3:1 propensity matching, beneficiaries with an opioid claim in the week after hospital discharge (with or without NSAID claims) were matched to beneficiaries with an NSAID claim only. Primary outcomes included death, healthcare utilization (emergency department [ED] visits and rehospitalization), and a composite of known adverse effects of opioids or NSAIDs (fall/fracture, delirium, nausea/vomiting, complications of slowed colonic motility, acute renal failure, and gastritis/duodenitis) within 30 days of discharge. After propensity matching, there were 13,385 beneficiaries in the opioid cohort and 4,677 in the NSAID cohort (mean age: 74 years, 57% female). Beneficiaries receiving opioids had a higher incidence of death (1.8% versus 1.1%; relative risk [RR] 1.7 [1.3 to 2.3], *p* < 0.001, number needed to harm [NNH] 125), healthcare utilization (19.0% versus 17.4%; RR 1.1 [1.02 to 1.2], *p* = 0.02, NNH 59), and any potential adverse effect (25.2% versus 21.3%; RR 1.2 [1.1 to 1.3], *p* < 0.001, NNH 26), compared to those with an NSAID claim only. Specifically, they had higher relative risk of fall/fracture (4.5% versus 3.4%; RR 1.3 [1.1 to 1.6], *p* = 0.002), nausea/vomiting (9.2% versus 7.3%; RR 1.3 [1.1 to 1.4], *p* < 0.001), and slowed colonic motility (8.0% versus 6.2%; RR 1.3 [1.1 to 1.4], *p* < 0.001). Risks of delirium, acute renal failure, and gastritis/duodenitis did not differ between groups. The main limitation of our study is the observational nature of the data and possibility of residual confounding.

**Conclusions:**

Older adults filling an opioid prescription in the week after hospital discharge were at higher risk for mortality and other post-discharge adverse outcomes compared to those filling an NSAID prescription only.

## Abstract 2

In a retrospective cohort study, Shoshana Herzig and colleagues investigate the incidence and risk of post-hospital discharge adverse events among those with claims for opioid analgesics compared to NSAIDs among older Medicare beneficiaries in the US.

## Author summary

**Why was this study done?:**

- Each year in the US, millions of older adults are hospitalized and prescribed medications intended for the treatment of pain on hospital discharge.
- Opioids and nonsteroidal anti-inflammatory drugs (NSAIDs) are among the most commonly used medications for the treatment of pain, but they carry risks.
- Studies comparing their risks during this vulnerable transition period are limited.

**What did the researchers do and find?:**

- We used records from the largest payer for healthcare in the US to study a nationwide sample of older adults who received an opioid or an NSAID prescription in the week after hospital discharge.
- We found that older adults filling an opioid prescription were at higher risk for death, healthcare utilization, falls/fractures, nausea/vomiting, and complications related to constipation, compared to those filling an NSAID prescription.
- Risk of acute renal failure and upper gastrointestinal complications did not differ between the groups.

**What do these findings mean?:**

- Our findings suggest that compared to opioids, NSAIDs are associated with fewer medication-related harms to older adults after discharge from the hospital; however, given that causality cannot be inferred from an observational study, these results should be tested in future studies.
- Additional research on the effectiveness of these medications among older adults hospitalized with various conditions would help to further understand overall risk to benefit ratio.

## Introduction

Pain is highly prevalent in the hospital setting [1,2], and opioid use is common, occurring in more than half of hospitalized patients [3,4]. Inpatient opioid use is often continued after discharge, with 15% of previously opioid naïve older adults filling a new opioid prescription within 7 days of hospital discharge [5]. With more than 12 million discharges age 65 and older from US hospitals each year, this suggests that almost 2 million older adults are newly initiated on opioids after a hospitalization annually.

In the context of the opioid crisis, guidelines have increasingly emphasized use of nonopioid analgesics, like nonsteroidal anti-inflammatory drugs (NSAIDs), instead of opioid analgesics whenever possible [6,7]. Clinicians, however, are often hesitant to use NSAIDs, owing to concerns over adverse events [8], including upper gastrointestinal complications and renal failure, which are particularly common among hospitalized adults. Whether these risks are greater than the risks posed by opioids, including delirium, falls, and slowed gastrointestinal motility, is uncertain. This is particularly true in the post-hospitalization period, in which patients are already at heightened risk for these adverse outcomes. Additionally, hospitalized adults tend to be older than the general population and at higher risk for adverse effects of medications in general [9].

Using a national sample of hospitalized older adults, we aimed to determine the incidence of 30-day post-discharge adverse events among those filling an opioid claim in the week after hospital discharge, compared to those filling a claim for an NSAID only. We hypothesized that risks of adverse events would be higher among beneficiaries with an opioid claim compared to those with an NSAID claim, with the exception of renal and upper gastrointestinal complications.

## Methods

The study was approved by the Beth Israel Deaconess Medical Center Institutional Review Board with a waiver of informed consent. This study is reported as per the Strengthening the Reporting of Observational Studies in Epidemiology (STROBE) guideline (S1 Checklist). The prospective study protocol is available in the Supporting information section (S1 Protocol).

### Study population

We conducted a cohort study of the 20% sample of US Medicare beneficiaries with a hospitalization in 2016 using the Center for Medicare and Medicaid Services Medicare Provider Analysis and Review file. We included traditional Medicare beneficiaries who were at least 65 years old and had been continuously enrolled in Medicare Parts A, B, and D for at least 1 year prior and 1 month following discharge. We excluded beneficiaries who died during hospitalization, were discharged to hospice, or had hospice claims within 12 months before or 1 week after discharge. We also excluded beneficiaries who had skilled nursing facility (SNF) claims in the month before hospitalization, or were transferred in from, or discharged to any type of facility, as medication claims were unavailable during these periods. After identifying beneficiaries with an opioid or NSAID claim within 7 days of discharge, we randomly chose a single hospitalization per beneficiary, to avoid correlated observations.

### Opioid and NSAID exposure definitions

We used the Centers for Disease Control (CDC) National Center for Injury Prevention and Control compilation of opioid analgesics (S1 Table) to identify opioid claims occurring within 7 days after hospital discharge [10]. Because we were interested in opioids used for treatment of pain, we excluded claims for buprenorphine formulations intended for treatment of opioid use disorder, as defined by the CDC algorithm [10]. Methadone claims for treatment of opioid use disorder were not included in the dataset. We used the American Hospital Formulary Service to identify both selective (cyclooxygenase-2 [COX-2] inhibitors) and nonselective NSAID claims (S1 Table) occurring within 7 days after hospital discharge. Our main analyses treated opioid and NSAID exposure hierarchically, comparing beneficiaries with an opioid claim within 7 days of hospital discharge (regardless of exposure to NSAIDs) to beneficiaries with an NSAID claim only. We chose this as our main comparison to reflect the standard of care in opioid therapy, since guidelines recommend co-prescribing opioids with nonopioid analgesics whenever possible [6,7].

### Outcomes

We measured the occurrence of adverse outcomes in the time period between the date of the first claim for an opioid/NSAID and 30 days after hospital discharge. Our primary outcomes were (1) death; (2) healthcare utilization, defined as any emergency department (ED) visit or rehospitalization (including inpatient and observation status); and (3) a composite of any known adverse effect of opioids or NSAIDs, including falls/fractures (grouped), delirium, nausea/vomiting (grouped), slowed colonic motility (constipation/ileus/impaction/obstruction, grouped), acute renal failure, and gastritis/duodenitis (including inflammation/ulcer/bleeding, grouped), all defined using the International Classification of Diseases (ICD) 9 or 10 codes occurring in any position in either the inpatient or outpatient setting, plus related medication claims where noted (see S2 Table for ICD codes and medications). We examined the incidence of each prespecified adverse effect as secondary outcomes.

### Covariates

We included covariates hypothesized to be associated with our exposures and outcomes of interest. These included (1) demographics (age, gender, race, and Medicaid eligibility); (2) diagnoses over the year prior to hospital discharge (including any primary or secondary discharge diagnoses from the index hospitalization), including a combination of Elixhauser comorbidities operationalized using the algorithm from Quan and colleagues [11] and the Centers for Medicare and Medicaid Services (CMS) Chronic Conditions Warehouse (CCW), with the addition of factors reflecting history of dementia, falls/fractures, delirium, nausea/vomiting, slowed colonic motility, acute renal failure, and gastritis/duodenitis, using the same diagnosis codes and medications as specified for our outcome variables (see S2 Table); (3) frailty/function over the year prior to hospital discharge, including hospital discharge diagnoses and claims within 2 days of discharge, including the claims-based frailty index [12,13], home healthcare claims, SNF claims, and/or mobility impairment, operationalized using the CMS CCW; (4) hospitalization characteristics; (5) primary discharge diagnosis, grouped using the Healthcare Cost and Utilization Project multilevel diagnosis and procedure Clinical Classification System [14,15]; (6) number of prior hospitalizations; and (7) prior and concurrent medication use (see S3 Table for a full list of covariates).

### Statistical analysis

We performed propensity score matching to control for differences between patients discharged with opioids and patients discharged with NSAIDS only, as follows. First, we ran a multivariable logistic regression model with a Firth correction factor, wherein opioid versus NSAID exposure was the dependent variable, and all of the covariates described above were independent variables. The fitted probabilities from this model reflect the propensity for each beneficiary to have been exposed to an opioid. Beneficiaries in the 2 exposure groups were then matched on their propensity score using a greedy matching technique, without replacement, using a caliper width of 0.2 times the standard deviation of the logit of the propensity score [16]. We required an exact match on days from discharge to first medication claim to avoid differences in the observation period between groups. Because the number of opioid-exposed beneficiaries was far greater than the number of NSAID-exposed beneficiaries, we matched each NSAID-exposed beneficiary with up to 3 opioid-exposed beneficiaries. Characteristics were compared across the matched groups using standardized mean differences (SMDs), with an SMD >0.1 indicating a clinically important difference. We calculated the absolute difference in incidence of each outcome between the matched opioid and NSAID groups, and for outcomes with a significant difference, we calculated the number needed to harm (NNH) as the inverse of the absolute difference. We used a generalized linear model with a binomial distribution and a log link to generate the relative risk of each outcome in the opioid versus NSAID group. We accounted for the fact that not all NSAID-exposed beneficiaries could be matched to 3 opioid-exposed beneficiaries by weighting all matches by 1 divided by the number of opioid users able to be matched for that particular cluster.

All analyses were carried out using version 9.4 of SAS software (SAS Institute, Cary, North Carolina, US).

### Subgroup analyses

We reran our propensity model among 5 prespecified subgroups of beneficiaries: (1) those without any opioid or NSAID claims in the 90 days prior to hospitalization (NSAID claims exclusion was added during the peer review process); (2) those without a history of opioid use disorder or long-term high-dose opioid use (added during the peer review process); (3) those with a medical reason for hospitalization (based on the diagnosis-related group [DRG]); (4) those with a surgical reason for hospitalization; and (5) those without a diagnosis of cancer, defined by presence of any of the Elixhauser comorbidities of lymphoma, metastatic cancer, or solid tumor without metastasis [10].

### Sensitivity analyses

Because our main analysis allowed for NSAID exposure in the opioid group, we performed a sensitivity analysis in which beneficiaries with claims for both opioids and NSAIDs within 7 days of discharge were excluded, creating mutually exclusive comparator groups. We then reran our propensity model using the same covariates as in the primary analysis.

Additionally, because propensity matching results in loss of beneficiaries for analysis (the maximum number matched is limited by the smaller of the exposure groups), we ran a secondary analysis in the full (pre-match) opioid and NSAID cohorts, including all covariates as independent variables, plus a variable representing opioid versus NSAID exposure. Because of nonconvergence of the log-binomial model in the setting of many predictors [17], we instead used a logistic regression model with Firth correction, which estimates odds ratios instead of relative risks. To facilitate direct comparisons, we reanalyzed the propensity-matched cohorts using a logit link.

Finally, using the E-value [18,19], we estimated the strength of association between an unmeasured confounder and both opioid use and death that would be needed to explain away the observed risk of death from opioid use.

## Results

**Fig 1:**
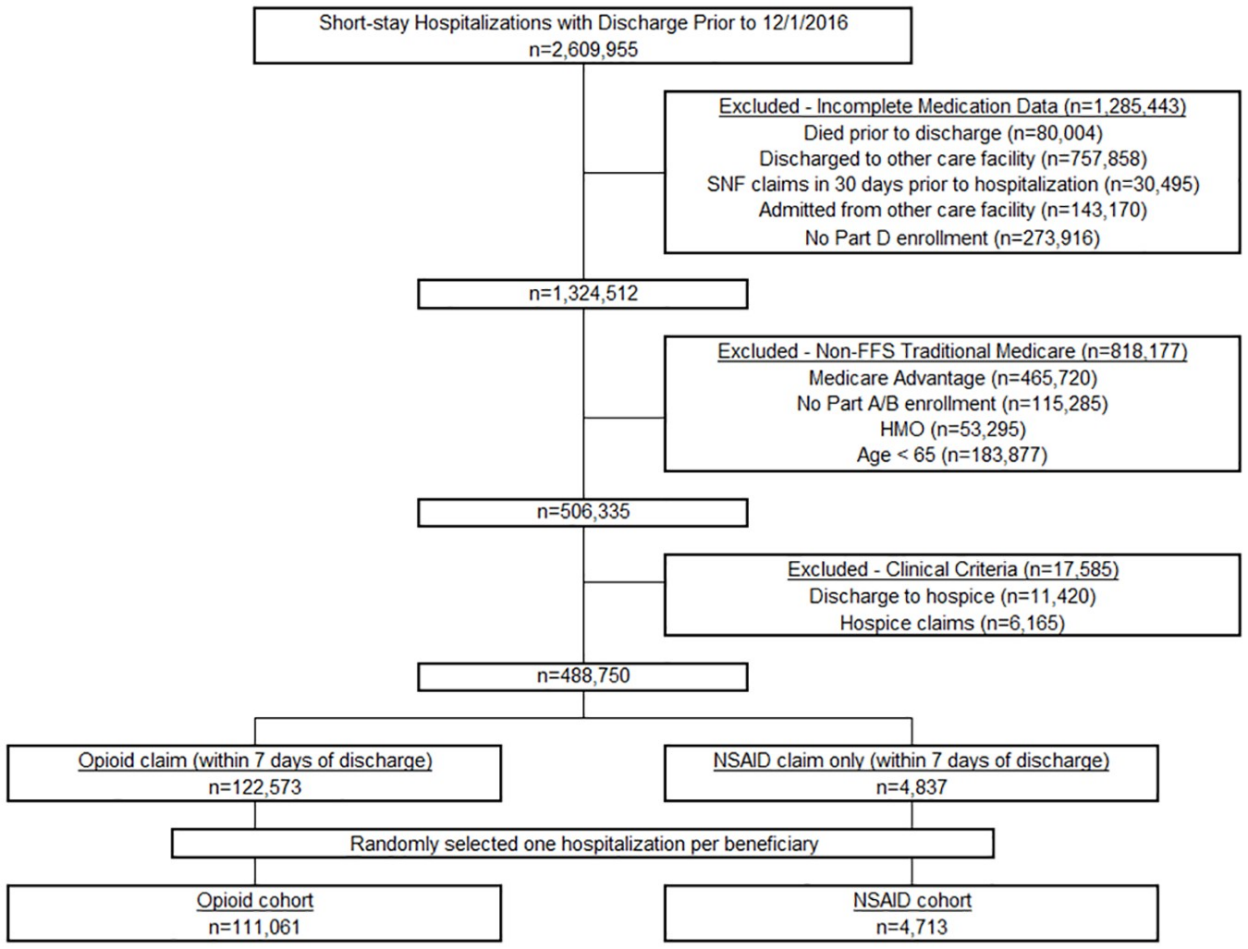
Study consort diagram. CMS, Centers for Medicare and Medicaid Services; FFS, fee for service; HMO, health maintenance organization; NSAID, nonsteroidal anti-inflammatory drug; SNF, skilled nursing facility.

Fig 1 shows the study flow diagram. After applying exclusion criteria, there were 488,750 hospitalizations remaining, of which 122,573 (25.1%) had a claim for an opioid within 7 days of discharge, and 4,837 had an NSAID claim only. After randomly selecting 1 hospitalization per beneficiary, there were 115,774 hospitalizations/beneficiaries in our final analytic sample: 111,061 with an opioid claim within 7 days of discharge (opioid cohort) and 4,713 with an NSAID claim only (NSAID cohort). Select beneficiary characteristics are shown in Table 1 (see S3 Table for the full list of characteristics and S1 Fig for SMDs).

**Table 1:** Characteristics of study population, before and after propensity matching (see S1 Fig for SMDs; all were <0.1 after the match).

	Before propensity matching				After propensity matching			
	Opioid		NSAID		Opioid		NSAID	
Characteristic—*n* % unless otherwise noted	*n* = 111,061		*n* = 4,713		*n* = 13,385		*n* = 4,677	
Age in years—mean, SD	74.3	6.5	75.9	7.5	75.8	7.5	75.9	7.5
Male	47,414	42.7	1,703	36.1	4,840	36.2	1,689	36.1
Race								
Black	8,483	7.6	494	10.5	1,467	11.0	489	10.5
White	96,776	87.1	3,742	79.4	10,646	79.5	3,719	79.5
Other	5,802	5.2	477	10.1	1,272	9.5	469	10.0
Medicaid dual eligible	21,186	19.1	1,694	35.9	4,636	34.6	1,666	35.6
Prior diagnoses								
Congestive heart failure	23,398	21.1	1,165	24.7	3,481	26.0	1,158	24.8
Liver disease	6,903	6.2	266	5.6	791	5.9	263	5.6
Lymphoma	2,155	1.9	73	1.5	221	1.7	73	1.6
Metastatic cancer	6,793	6.1	195	4.1	636	4.8	195	4.2
Solid tumor without metastasis	22,971	20.7	714	15.1	2,127	15.9	711	15.2
Psychoses	1,398	1.3	211	4.5	479	3.6	195	4.2
Depression	25,269	22.8	1,312	27.8	3,718	27.8	1,297	27.7
Bipolar disorder	1,989	1.8	151	3.2	416	3.1	148	3.2
Anxiety disorder	21,766	19.6	1,098	23.3	3,101	23.2	1,086	23.2
Opioid use disorder	10,301	9.3	414	8.8	1,287	9.6	410	8.8
Drug use disorder	2,081	1.9	135	2.9	380	2.8	134	2.9
Falls/fractures	56	0.1	-*	-*	16	0.1	-*	-*
Delirium	6,020	5.4	412	8.7	1,178	8.8	405	8.7
Nausea/vomiting	24,102	21.7	985	20.9	2,868	21.4	978	20.9
Slowed colonic motility^†^	25,871	23.3	1,062	22.5	3,116	23.3	1,051	22.5
Acute renal failure	17,516	15.8	816	17.3	2,465	18.4	810	17.3
Gastritis/duodenitis^‡^	9,655	8.7	393	8.3	1,222	9.1	392	8.4
Frailty/function								
Frailty index—mean, SD	0.2	0.1	0.2	0.1	0.2	0.1	0.2	0.1
Home healthcare claims	20,848	18.8	1,239	26.3	3,593	26.8	1,225	26.2
SNF claims	6,491	5.8	315	6.7	963	7.2	312	6.7
Mobility impairment	2,832	2.5	167	3.5	469	3.5	166	3.5
Hospitalization characteristics								
Length of stay—mean, SD	3.7	3.7	3.7	4.4	3.7	3.4	3.7	4.3
Any time in intensive care	22,629	20.4	1,045	22.2	2,945	22.0	1,036	22.2
Diagnosis-related group								
Medical	36,789	33.1	3,438	73.0	9,665	72.2	3,402	72.7
Surgical	74,272	66.9	1,275	27.1	3,720	27.8	1,275	27.3
Most common primary discharge diagnoses								
Diseases of the circulatory system	16,604	15.0	963	20.4	2,678	20.0	958	20.5
Diseases of the musculoskeletal system and connective tissue	36,243	32.6	621	13.2	1,734	13.0	621	13.3
Diseases of the respiratory system	5,726	5.2	625	13.3	1,740	13.0	618	13.2
Diseases of the digestive system	12,708	11.4	498	10.6	1,458	10.9	496	10.6
Most common primary discharge procedures								
Operations on the musculoskeletal system	38,892	35.0	617	13.1	1,724	12.9	617	13.2
Operations on the cardiovascular system	13,415	12.1	446	9.5	1,314	9.8	445	9.5
Miscellaneous diagnostic and therapeutic procedures	4,951	4.5	454	9.6	1,249	9.3	447	9.6
Operations on the digestive system	14,884	13.4	365	7.7	1,107	8.3	364	7.8
Number of prior hospitalizations—mean, SD	0.7	1.5	0.8	1.5	0.9	1.6	0.8	1.5
Medication use in prior 90 d								
Number of claims—mean, SD	12.2	9.8	16.5	12.8	16.1	12.6	16.4	12.6
Medication use within 7 d of discharge								
Number of claims—mean, SD	3.3	2.4	4.6	3.4	4.4	3.2	4.5	3.4

* Cell suppressed owing to small cell size, in accordance with CMS policy.

^†^ Includes constipation, ileus, impaction, and obstruction.

^‡^ Includes gastric or duodenal inflammation or ulcer or upper gastrointestinal bleeding.

CMS, Centers for Medicare and Medicaid Services; d, days; NSAID, nonsteroidal anti-inflammatory drug; SD, standard deviation; SMD, standardized mean difference; SNF, skilled nursing facility.

### Propensity-matched analysis

We matched 4,677 (99.2%) beneficiaries in the NSAID cohort to at least 1 beneficiary in the opioid cohort: 4,239 (90.6%) were matched 1:3, 230 (4.9%) 1:2, and 208 (4.5%) 1:1. The propensity score distributions by group, before and after the match, are shown in S2 Fig. After the match, the opioid and NSAID cohorts were well balanced on all characteristics in Table 1 (see S3 Table for the full list of characteristics and S1 Fig for SMDs, all of which were <0.1). The most commonly prescribed opioids were hydrocodone, oxycodone, and tramadol; the most commonly prescribed NSAIDs were meloxicam, ibuprofen, and celecoxib (S1 Table).

Table 2 shows the outcome incidence, absolute risk difference, and relative risk of each primary outcome for opioids compared to NSAIDs in the propensity-matched cohorts. The relative risks of death (1.7, 95% CI 1.3 to 2.3, *p* < 0.001; NNH 125), healthcare utilization (1.1, 95% CI 1.02 to 1.2, *p* = 0.02; NNH 59), and any potential adverse effect (1.2, 95% CI 1.1 to 1.3, *p* < 0.001; NNH 26) were significantly higher in the opioid versus the NSAID cohort.

**Table 2:** Outcome incidence, absolute risk difference, and relative risk for opioids compared to NSAIDs in the propensity-matched cohorts (*n* = 18,062).

	Opioid		NSAID					
	*n* = 13,385		*n* = 4,677		Absolute risk difference (%)	Relative risk	*p*-value	NNH*
Primary outcomes^†^	*n*	%	*n*	%	[95% CI]	[95% CI]		
Death	246	1.8	50	1.1	0.8 [0.4 to 1.1]	1.7 [1.3 to 2.3]	<0.001	125
Healthcare utilization	2,549	19.0	813	17.4	1.7 [0.3 to 3.1]	1.1 [1.02 to 1.2]	0.02	59
Any potential adverse effect^‡^	3,374	25.2	995	21.3	3.9 [2.4 to 5.5]	1.2 [1.1 to 1.3]	<0.001	26

* NNH calculated only for outcomes demonstrating significant difference between opioid and NSAID exposed.

^†^ Measured from date of the medication claim to 30 days after hospital discharge.

^‡^ Any of the following: fall/fracture, delirium, nausea/vomiting, slowed colonic motility (constipation, ileus, impaction, and obstruction), acute renal failure, and gastritis/duodenitis.

CI, confidence interval; NNH, number needed to harm; NSAID, nonsteroidal anti-inflammatory drug.

With respect to the secondary outcomes of the individual adverse effects (Table 3), those filling an opioid claim had significantly higher relative risk of fall/fracture (1.3, 95% CI 1.1 to 1.6, *p* = 0.002; NNH 91), nausea/vomiting (1.3, 95% CI 1.1 to 1.4, *p* < 0.001; NNH 53), and slowed colonic motility (1.3, 95% CI 1.1 to 1.4, *p* < 0.001; NNH 59). The risks of delirium, acute renal failure, and gastritis/duodenitis were not significantly different between the groups.

**Table 3:** Incidence, absolute risk difference, and relative risk of individual adverse effects for opioids compared to NSAIDs in the propensity-matched cohorts (*n* = 18,062).

	Opioid		NSAID					
	*n* = 13,385		*n* = 4,677		Absolute risk difference (%)	Relative risk	*p*-value	NNH*
Individual adverse effects^†^	*n*	%	*n*	%	[95% CI]	[95% CI]		
Fall/fracture	604	4.5	158	3.4	1.1 [0.5 to 1.8]	1.3 [1.1 to 1.6]	0.002	91
Delirium	335	2.5	101	2.2	0.3 [−0.2 to 0.8]	1.2 [0.98 to 1.5]	0.07	-
Nausea/vomiting	1,225	9.2	339	7.3	1.9 [1.0 to 2.8]	1.3 [1.1 to 1.4]	<0.001	53
Slowed colonic motility^‡^	1,068	8.0	292	6.2	1.7 [0.9 to 2.6]	1.3 [1.1 to 1.4]	<0.001	59
Acute renal failure	657	4.9	220	4.7	0.2 [−0.5 to 0.9]	1.0 [0.9 to 1.2]	0.74	-
Gastritis/duodenitis^§^	558	4.2	207	4.4	−0.3 [−1.0 to 0.4]	0.9 [0.8 to 1.1]	0.28	-

* NNH calculated only for outcomes demonstrating significant difference between opioid and NSAID exposed.

^†^ Measured from date of the medication claim to 30 days after hospital discharge.

^‡^ Includes constipation, ileus, impaction, and obstruction.

^§^ Includes gastric or duodenal inflammation or ulcer or upper gastrointestinal bleeding.

CI, confidence interval; NNH, number needed to harm; NSAID, nonsteroidal anti-inflammatory drug.

### Subgroup analyses

Allowing for differences in sample size and associated precision, point estimates were similar across our 5 prespecified patient subgroups: (1) those without any opioid or NSAID claims in the 90 days prior to hospitalization; (2) those without a history of opioid use disorder or long-term high-dose opioid use; (3) those with a medical reason for hospitalization; (4) those with a surgical reason for hospitalization; and (5) those without a diagnosis of cancer (Table 4, S4–S7 Tables, S1 and S2 Figs).

**Table 4:** Relative risk for opioids compared to NSAIDs in the propensity-matched cohorts of prespecified subgroups.

	Mutually exclusive exposure groups	Analgesic-naive beneficiaries*	Beneficiaries without OUD or long-term high-dose opioid use	Medical hospitalizations	Surgical hospitalizations	Beneficiaries without cancer
	*n* = 17,884	*n* = 5,582	*n* = 16,160	*n* = 12,873	*n* = 5,047	*n* = 14,947
	Relative risk	Relative risk	Relative risk	Relative risk	Relative risk	Relative risk
Outcome	[95% CI], *p*-value	[95% CI], *p*-value	[95% CI], *p*-value	[95% CI], *p*-value	[95% CI], *p*-value	[95% CI], *p*-value
Death	1.8 [1.3 to 2.4], <0.001	1.5 [0.9 to 2.5], 0.11	1.6 [1.2 to 2.2], 0.003	1.6 [1.2 to 2.2], 0.003	1.7 [0.6 to 4.3], 0.30	1.6 [1.1 to 2.4], 0.02
Healthcare utilization	1.1 [1.02 to 1.2], 0.01	1.1 [0.97 to 1.3], 0.13	1.1 [0.99 to 1.2], 0.06	1.1 [0.99 to 1.2], 0.10	1.2 [1.02 to 1.5], 0.03	1.1 [1.00 to 1.2], 0.06
Any potential adverse effect	1.2 [1.1 to 1.3], <0.001	1.3 [1.2 to 1.5], <0.001	1.2 [1.1 to 1.3], <0.001	1.2 [1.1 to 1.2], <0.001	1.3 [1.1 to 1.5], 0.002	1.2 [1.1 to 1.2], <0.001
Fall/fracture	1.3 [1.1 to 1.5], 0.003	1.6 [1.2 to 2.2], 0.005	1.2 [0.9 to 1.5], 0.15	1.4 [1.1 to 1.7], 0.001	1.1 [0.8 to 1.5], 0.58	1.3 [1.1 to 1.6], 0.006
Delirium	1.2 [0.99 to 1.5], 0.06	1.3 [0.8 to 2.0], 0.27	1.1 [0.9 to 1.5], 0.28	1.4 [1.1 to 1.7], 0.009	1.7 [0.7 to 3.7], 0.22	1.3 [1.01 to 1.6], 0.04
Nausea/vomiting	1.3 [1.2 to 1.5], <0.001	1.6 [1.3 to 2.1], <0.001	1.3 [1.2 to 1.5], <0.001	1.2 [1.1 to 1.4], 0.002	1.4 [1.1 to 1.8], 0.01	1.2 [1.1 to 1.4], 0.002
Slowed colonic motility^†^	1.3 [1.1 to 1.5], <0.001	1.4 [1.1 to 1.8], 0.006	1.4 [1.2 to 1.5], <0.001	1.2 [1.1 to 1.4], 0.008	1.7 [1.2 to 2.3], 0.002	1.2 [1.1 to 1.4], 0.007
Acute renal failure	1.1 [0.9 to 1.2], 0.54	1.2 [0.9 to 1.6], 0.26	1.0 [0.8 to 1.1], 0.70	1.0 [0.9 to 1.2], 0.99	1.0 [0.7 to 1.5], 0.89	1.0 [0.8 to 1.1], 0.64
Gastritis/duodenitis^‡^	0.9 [0.8 to 1.1], 0.41	1.1 [0.8 to 1.4], 0.69	1.0 [0.8 to 1.1], 0.77	0.9 [0.8 to 1.1], 0.50	1.0 [0.7 to 1.6], 0.98	0.9 [0.7 to 1.04], 0.14

* Defined as having no opioid or NSAID claims in the 90 days prior to hospital admission.

^†^ Includes constipation, ileus, impaction, and obstruction.

^‡^ Includes gastric or duodenal inflammation or ulcer or upper gastrointestinal bleeding.

CI, confidence interval; NSAID, nonsteroidal anti-inflammatory drug; OUD, opioid use disorder.

### Sensitivity analyses

Among the 111,061 hospitalizations with an opioid claim within 7 days of hospital discharge, we found that 6,355 (5.7%) also had claims for NSAIDs within 7 days of discharge. After excluding these hospitalizations and rerunning our 3:1 propensity match using the same covariates, our results were almost identical to the primary analysis (Table 4, S8 Table, S1 and S2 Figs). Examining each outcome in the full cohort (i.e., before propensity matching), with adjustment for all covariates (S9 Table), also yielded similar results to the primary analyses.

We determined that the observed relative risk of death of 1.7 could be explained by an unmeasured confounder that was associated with both opioid use and death by a relative risk of at least 2.8, above and beyond the measured confounders; the confidence interval could be moved to include the null by an unmeasured confounder that was associated with both treatment and outcome by a relative risk of 1.9.

## Discussion

In a national sample of Medicare beneficiaries, we found that those who filled an opioid prescription in the week after hospital discharge were at higher risk for post-discharge adverse outcomes compared to beneficiaries who filled an NSAID prescription. Specifically, they had higher risk of death, healthcare utilization, falls/fractures, nausea/vomiting, and complications related to slowed colonic motility. We found no difference in risk of acute renal failure or upper gastrointestinal complications. These results were robust to multiple sensitivity analyses and across multiple patient subgroups.

Recommendations promoting the use of nonopioid analgesics instead of opioid analgesics whenever possible [6,7] are based on trials suggesting that for many common painful conditions, compared to NSAIDs, opioids are generally no more efficacious [20–23], are more likely to lead to adverse events [21,23,24], and may be associated with worse functional outcomes [23,25]. However, the evidence in many of these trials was of low quality and at high risk of both selection and publication bias [22,23]. Key outcomes were often not measured. For example, in a systematic review of 20 randomized trials in patients with acute renal colic, the authors note that rates of gastrointestinal bleeding and renal impairment were not reported [21]. Additionally, results of trials, most of which were in highly select surgical patient populations, may fail to reflect the true incidence of adverse events in real-world clinical practice, including medically complex, nonsurgical patients. Furthermore, none specifically examined older adults in the post-hospitalization time period—a highly vulnerable patient population and time period for adverse drug events [9]. Because of these knowledge gaps, national guidelines have called for additional research on the comparative effectiveness and safety of opioid therapy relative to nonopioid alternatives [7]. Our results help to fill this knowledge gap, providing estimates of the risks associated with opioids and NSAIDs among older adults in the post-hospitalization period.

Concerns regarding use of NSAIDs often relate to potential renal adverse effects. Notably, our analysis did not find higher rates of acute renal failure among patients with an NSAID claim in the week after hospital discharge, relative to those with an opioid claim. Although we adjusted for several predictors of renal failure, including preexisting chronic kidney disease (CKD), prior episodes of acute renal failure, and use of angiotensin converting enzyme inhibitors and receptor blockers, it is still possible that our results reflect confounding by the fact that physicians tend to avoid NSAIDs in patients at high risk for adverse renal effects. However, a recent analysis of older adults did not find increased risk of kidney dysfunction or injury among those who reported taking NSAIDs [26]. Another recent analysis of patients with CKD found that opioid use had a stronger association with renal adverse events than NSAIDs [27]. Although opioids are not known to have direct effects on the kidneys, they may indirectly promote renal injury through other known adverse effects, including poor oral intake related to nausea/vomiting, slowed colonic motility, and hospitalization.

Another potential explanation for the null findings with respect to both renal failure and upper gastrointestinal complications could be exposure misclassification resulting from patients using NSAIDs purchased without a prescription (i.e., “over-the-counter”). We believe such exposure misclassification is unlikely to have explained our results because empiric evidence, based on our mutually exclusive exposure subgroup analysis, demonstrated that exclusion of known NSAID exposures from the opioid group had no effect on the relative risk of acute renal failure or upper gastrointestinal complications for opioids compared to NSAIDs. We would thus expect that further exclusion of beneficiaries using NSAIDs without a prescription would similarly not affect the relative risk.

We are unable to comment on the overall risk to benefit ratio for opioids and NSAIDs in this setting since we lack data on effectiveness. It is possible that the increased risk of adverse events observed with opioid use in this analysis could be counterbalanced by improved pain control in select patients. That said, a growing body of literature in patients with post-operative pain, acute soft tissue injury, and acute renal colic, demonstrates that for the average patient, opioids are no more effective than NSAIDs [20–23].

With over 12 million discharges of patients age 65 and older from US hospitals each year [28], combined with our finding that 25% had an opioid claim in the week after hospital discharge, an estimated 3 million older adults fill an opioid prescription after hospital discharge each year in the US. Our data suggest that if these older adults were able to be discharged on NSAID therapy instead, more than 115,000 adverse events (NNH 26) and 24,000 deaths (NNH 125) could be potentially prevented each year. Since not all patients can safely receive NSAIDs, these estimates should be considered upper bounds.

There are several limitations to our analysis. First, use of claims data could have resulted in underestimation of adverse events. Use of medication claims to supplement some of the outcome events likely increased capture, but conditions like delirium are known to be under-captured by billing data. Second, although we performed extensive adjustment for possible confounders, with over 100 variables in our propensity match, due to the observational nature of our data, residual confounding, including confounding by indication and confounding by severity of illness, is an important potential source of bias in our analysis. For example, we could not account for pain severity, which could increase the risk of opioid receipt and various outcomes. We note, however, that with a relative risk of death of 1.7 for opioids compared to NSAIDs, a rather large relative risk of at least 1.9 between an unmeasured confounder and both opioid use and death, would be necessary to move the confidence interval to include the null. Although a randomized trial would be less susceptible to confounding, the infrequency of these adverse events makes it unlikely that a randomized trial would ever be sufficiently powered to provide robust incidence and effect estimates. Third, our analysis did not take into account the duration of use or dosage, both of which could be related to risk of adverse outcomes; however, our results should be reflective of the average opioid and NSAID prescription dosage and duration. Fourth, because our dataset did not include long-term follow-up, we could not investigate other downstream adverse events, such as development of opioid use disorder. Finally, our results may not be generalizable beyond older adults with Medicare coverage.

In conclusion, in a large, national cohort of Medicare beneficiaries, we found that older adults who filled an opioid prescription in the week after hospital discharge were at higher risk for various post-discharge adverse outcomes, including death, compared to older adults who filled an NSAID prescription. Risk of acute renal failure and upper gastrointestinal complications did not differ between the groups. Additional research on the comparative effectiveness of these medications among older adults hospitalized with various conditions is necessary to inform the overall risk to benefit ratio.

## Supporting information

S1 Table Opioid and NSAID exposures in the propensity-matched cohorts. NSAID, nonsteroidal anti-inflammatory drug.
(DOCX)

S2 Table Outcome and covariate operationalization. (DOCX)

S3 Table Characteristics of study population, before and after propensity matching. (DOCX)

S4 Table Subgroup analysis in beneficiaries without opioid claims in the 90 days prior to hospitalization. Characteristics of study population, before and after propensity matching.
(DOCX)

S5 Table Subgroup analysis in medical hospitalizations. Characteristics of study population, before and after propensity matching.
(DOCX)

S6 Table Subgroup analysis in surgical hospitalizations. Characteristics of study population, before and after propensity matching.
(DOCX)

S7 Table Subgroup analysis in beneficiaries without cancer. Characteristics of study population, before and after propensity matching.
(DOCX)

S8 Table Subgroup analysis using mutually exclusive exposure groups (i.e., after excluding beneficiaries with claims for both opioids and NSAIDs within 7 days of discharge, *n* = 6,355). Characteristics of study population, before and after propensity matching. NSAID, nonsteroidal anti-inflammatory drug.
(DOCX)

S9 Table Relative risks and odds ratios of outcomes for opioids compared to NSAIDs in specified sensitivity analyses. NSAID, nonsteroidal anti-inflammatory drug.
(DOCX)

S1 Fig SMDs, before and after each propensity match. SMD, standardized mean difference.
(DOCX)

S2 Fig Propensity score distributions by group, before and after the match. Density reflects the proportion of hospitalizations with a given propensity score.
(DOCX)

S1 Checklist Location in the manuscript of each STROBE guideline component. STROBE, Strengthening the Reporting of Observational Studies in Epidemiology.
(DOCX)

S1 Protocol This document describes the basic study protocol, as specified prior to conducting any analyses. (DOCX)

## Abbreviations

CCW: Chronic Conditions Warehouse
CDC: Centers for Disease Control
CKD: chronic kidney disease
CMS: Centers for Medicare and Medicaid Services
COX-2: cyclooxygenase-2
DRG: diagnosis-related group
ED: emergency department
ICD: International Classification of Diseases
NNH: number needed to harm
NSAID: nonsteroidal anti-inflammatory drug
RR: relative risk
SMD: standardized mean difference
SNF: skilled nursing facility
STROBE: Strengthening the Reporting of Observational Studies in Epidemiology
