# Supplementary material for: Relative risks of adverse events among older adults receiving opioids versus NSAIDs after hospital discharge: A nationwide cohort study
Source: PLoS Med. 2021 Sep 27;18(9):e1003804. doi: 10.1371/journal.pmed.1003804 (PMC8504723; doi:10.1371/journal.pmed.1003804)
Supplement: S1 Table — NSAID, nonsteroidal anti-inflammatory drug. (DOCX) [file pmed.1003804.s001.docx]

| **S1 Table. Opioid and NSAID exposures in the propensity-matched cohorts.** | | | |
| --- | --- | --- | --- |
| **Opioid Cohort (n=13,385)** | | **NSAID Cohort (n=4,677)** | |
| Drug | n (%) | Drug | n (%) |
| Hydrocodone | 5404 (40.4) | Meloxicam | 1296 (27.7) |
| Oxycodone | 4101 (30.6) | Ibuprofen | 1144 (24.5) |
| Tramadol | 2935 (21.9) | Celecoxib | 907 (19.4) |
| Codeine | 743 (5.6) | Naproxen | 600 (12.8) |
| Morphine | 401 (3.0) | Diclofenac | 344 (7.4) |
| Hydromorphone | 309 (2.3) | Indomethacin | 138 (3.0) |
| Fentanyl | 306 (2.3) | Ketorolac | 109 (2.3) |
| Methadone | 72 (0.5) | Nabumetone | 73 (1.6) |
| Buprenorphine | 39 (0.3) | Etodolac | 50 (1.1) |
| Tapentadol | 26 (0.2) | Sulindac | 30 (0.6) |
| Oxymorphone | 18 (0.1) | Other | 23 (0.5) |
| Other | 11 (0.1) |  |  |
