# Supplementary material for: Relative risks of adverse events among older adults receiving opioids versus NSAIDs after hospital discharge: A nationwide cohort study
Source: PLoS Med. 2021 Sep 27;18(9):e1003804. doi: 10.1371/journal.pmed.1003804 (PMC8504723; doi:10.1371/journal.pmed.1003804)
Supplement: S2 Table — (DOCX) [file pmed.1003804.s002.docx]

| **S2 Table. Outcome and Covariate Operationalization*** | | | |
| --- | --- | --- | --- |
| ***ICD*9** | **Description** | ***ICD*10** | **Description** |
| **Falls/Fractures** | | | |
| E8800 | Escalator | V00111A | Fall from in-line roller-skates, initial encounter |
| E8801 | Fall on or from sidewalk curb | V00111D | Fall from in-line roller-skates, subsequent encounter |
| E8809 | Other stairs or steps | V00111S | Fall from in-line roller-skates, sequela |
| E8810 | Fall from ladder | V00121A | Fall from non-in-line roller-skates, initial encounter |
| E8811 | Fall from scaffolding | V00121D | Fall from non-in-line roller-skates, subsequent encounter |
| E882 | Fall from or out of building or other structure | V00131A | Fall from skateboard, initial encounter |
| E8831 | Accidental fall into well | V00131D | Fall from skateboard, subsequent encounter |
| E8832 | Accidental fall into storm drain or manhole | V00131S | Fall from skateboard, sequela |
| E8839 | Fall into other hold or other opening in surface | V00141A | Fall from scooter (nonmotorized), initial encounter |
| E8840 | Fall from playground equipment | V00141D | Fall from scooter (nonmotorized), subsequent encounter |
| E8841 | Fall from cliff | V00141S | Fall from scooter (nonmotorized), sequela |
| E8842 | Fall from chair | V00151A | Fall from heelies, initial encounter |
| E8843 | Fall from wheelchair | V00151D | Fall from heelies, subsequent encounter |
| E8844 | Fall from bed | V00151S | Fall from heelies, sequela |
| E8845 | Fall from other furniture | V00181A | Fall from oth rolling-type pedestrian conveyance, init |
| E8846 | Fall from commode | V00181D | Fall from oth rolling-type pedestrian conveyance, subs |
| E8849 | Other fall from one level to another | V00181S | Fall from other rolling-type pedestrian conveyance, sequela |
| E885 | Fall on same level from slipping, tripping, or stumbling | V00211A | Fall from ice-skates, initial encounter |
| E8850 | Fall from scooter | V00211D | Fall from ice-skates, subsequent encounter |
| E8851 | Fall from roller skates | V00211S | Fall from ice-skates, sequela |
| E8852 | Fall from skateboard | V00221A | Fall from sled, initial encounter |
| E8853 | Fall from skis | V00221D | Fall from sled, subsequent encounter |
| E8854 | Falls from snowboard | V00221S | Fall from sled, sequela |
| E8859 | Fall from other slipping, tripping, or stumbling | V00281A | Fall from gliding-type pedestrian conveyance, init encntr |
| E8860 | Fall on same level from collision, pushing, or shoving, by or with other person in sports | V00281D | Fall from gliding-type pedestrian conveyance, subs encntr |
| E8869 | Fall on same level from collision, pushing, or shoving, by or with other person, other and unspecified | V00281S | Fall from other gliding-type pedestrian conveyance, sequela |
| E888 | Other and unspecified fall | V00311A | Fall from snowboard, initial encounter |
| E8880 | Fall resulting in striking against sharp object | V00311D | Fall from snowboard, subsequent encounter |
| E8881 | Fall resulting in striking against other object | V00311S | Fall from snowboard, sequela |
| E8888 | Other fall | V00321A | Fall from snow-skis, initial encounter |
| E8889 | Unspecified fall | V00321D | Fall from snow-skis, subsequent encounter |
| E9870 | Falling from high place, undetermined whether accidentally or purposely inflicted: residential premises | V00321S | Fall from snow-skis, sequela |
| E9871 | Falling from high place, undetermined whether accidentally or purposely inflicted: other man-made structures | V00381A | Fall from flat-bottomed pedestrian conveyance, init encntr |
| E9872 | Falling from high place, undetermined whether accidentally or purposely inflicted: Natural sites | V00381D | Fall from flat-bottomed pedestrian conveyance, subs encntr |
| E9879 | Falling from high place, undetermined whether accidentally or purposely inflicted: Unspecified site | V00381S | Fall from other flat-bottomed pedestrian conveyance, sequela |
| 808xx | Fracture of pelvis | V00811A | Fall from moving wheelchair (powered), initial encounter |
| 812xx | Fracture of humerus | V00811D | Fall from moving wheelchair (powered), subsequent encounter |
| 813xx | Fracture of radius and ulna | V00811S | Fall from moving wheelchair (powered), sequela |
| 814xx | Fracture of carpal bones | V00821A | Fall from baby stroller, initial encounter |
| 820xx | Fracture of neck of femur | V00821D | Fall from baby stroller, subsequent encounter |
| 821xx | Fracture of other and unspecified parts of femur | V00821S | Fall from baby stroller, sequela |
| 822xx | Fracture of patella | V00831A | Fall from motorized mobility scooter, initial encounter |
| 823xx | Fracture of tibia and fibula | V00831D | Fall from motorized mobility scooter, subsequent encounter |
| 824xx | Fracture of ankle | V00831S | Fall from motorized mobility scooter, sequela |
|  |  | V00891A | Fall from other pedestrian conveyance, initial encounter |
|  |  | V00891D | Fall from other pedestrian conveyance, subsequent encounter |
|  |  | V00891S | Fall from other pedestrian conveyance, sequela |
|  |  | W000XXA | Fall on same level due to ice and snow, initial encounter |
|  |  | W000XXD | Fall on same level due to ice and snow, subsequent encounter |
|  |  | W000XXS | Fall on same level due to ice and snow, sequela |
|  |  | W001XXA | Fall from stairs and steps due to ice and snow, init encntr |
|  |  | W001XXD | Fall from stairs and steps due to ice and snow, subs encntr |
|  |  | W001XXS | Fall from stairs and steps due to ice and snow, sequela |
|  |  | W002XXA | Oth fall from one level to another due to ice and snow, init |
|  |  | W002XXD | Oth fall from one level to another due to ice and snow, subs |
|  |  | W002XXS | Oth fall from one level to another due to ice and snow, sqla |
|  |  | W009XXA | Unspecified fall due to ice and snow, initial encounter |
|  |  | W009XXD | Unspecified fall due to ice and snow, subsequent encounter |
|  |  | W009XXS | Unspecified fall due to ice and snow, sequela |
|  |  | W010XXA | Fall same lev from slip/trip w/o strike against object, init |
|  |  | W010XXD | Fall same lev from slip/trip w/o strike against object, subs |
|  |  | W010XXS | Fall same lev from slip/trip w/o strike agnst object, sqla |
|  |  | W0110XA | Fall same lev from slip/trip w strike agnst unsp obj, init |
|  |  | W0110XD | Fall same lev from slip/trip w strike agnst unsp obj, subs |
|  |  | W0110XS | Fall same lev from slip/trip w strike agnst unsp obj, sqla |
|  |  | W01110A | Fall same lev from slip/trip w strk agnst sharp glass, init |
|  |  | W01110D | Fall same lev from slip/trip w strk agnst sharp glass, subs |
|  |  | W01110S | Fall same lev from slip/trip w strk agnst sharp glass, sqla |
|  |  | W01111A | Fall same lev from slip/trip w strk agnst pwr tl/machn, init |
|  |  | W01111D | Fall same lev from slip/trip w strk agnst pwr tl/machn, subs |
|  |  | W01111S | Fall same lev from slip/trip w strk agnst pwr tl/machn, sqla |
|  |  | W01118A | Fall same lev fr slip/trip w strk agnst oth sharp obj, init |
|  |  | W01118D | Fall same lev fr slip/trip w strk agnst oth sharp obj, subs |
|  |  | W01118S | Fall same lev fr slip/trip w strk agnst oth sharp obj, sqla |
|  |  | W01119A | Fall same lev fr slip/trip w strk agnst unsp sharp obj, init |
|  |  | W01119D | Fall same lev fr slip/trip w strk agnst unsp sharp obj, subs |
|  |  | W01119S | Fall same lev fr slip/trip w strk agnst unsp sharp obj, sqla |
|  |  | W01190A | Fall same lev from slip/trip w strike agnst furniture, init |
|  |  | W01190D | Fall same lev from slip/trip w strike agnst furniture, subs |
|  |  | W01190S | Fall same lev from slip/trip w strike agnst furniture, sqla |
|  |  | W01198A | Fall same lev from slip/trip w strike agnst oth object, init |
|  |  | W01198D | Fall same lev from slip/trip w strike agnst oth object, subs |
|  |  | W01198S | Fall same lev from slip/trip w strike agnst oth object, sqla |
|  |  | W03XXXA | Oth fall same lev due to collision w another person, init |
|  |  | W03XXXD | Oth fall same lev due to collision w another person, subs |
|  |  | W03XXXS | Oth fall same lev due to collision w another person, sequela |
|  |  | W04XXXA | Fall while being carried or supported by oth persons, init |
|  |  | W04XXXD | Fall while being carried or supported by oth persons, subs |
|  |  | W04XXXS | Fall while being carried or supported by oth persons, sqla |
|  |  | W050XXA | Fall from non-moving wheelchair, initial encounter |
|  |  | W050XXD | Fall from non-moving wheelchair, subsequent encounter |
|  |  | W050XXS | Fall from non-moving wheelchair, sequela |
|  |  | W051XXA | Fall from non-moving nonmotorized scooter, initial encounter |
|  |  | W051XXD | Fall from non-moving nonmotorized scooter, subs encntr |
|  |  | W051XXS | Fall from non-moving nonmotorized scooter, sequela |
|  |  | W052XXA | Fall from non-moving motorized mobility scooter, init encntr |
|  |  | W052XXD | Fall from non-moving motorized mobility scooter, subs encntr |
|  |  | W052XXS | Fall from non-moving motorized mobility scooter, sequela |
|  |  | W06XXXA | Fall from bed, initial encounter |
|  |  | W06XXXD | Fall from bed, subsequent encounter |
|  |  | W06XXXS | Fall from bed, sequela |
|  |  | W07XXXA | Fall from chair, initial encounter |
|  |  | W07XXXD | Fall from chair, subsequent encounter |
|  |  | W07XXXS | Fall from chair, sequela |
|  |  | W08XXXA | Fall from other furniture, initial encounter |
|  |  | W08XXXD | Fall from other furniture, subsequent encounter |
|  |  | W08XXXS | Fall from other furniture, sequela |
|  |  | W090XXA | Fall on or from playground slide, initial encounter |
|  |  | W090XXD | Fall on or from playground slide, subsequent encounter |
|  |  | W090XXS | Fall on or from playground slide, sequela |
|  |  | W091XXA | Fall from playground swing, initial encounter |
|  |  | W091XXD | Fall from playground swing, subsequent encounter |
|  |  | W091XXS | Fall from playground swing, sequela |
|  |  | W092XXA | Fall on or from jungle gym, initial encounter |
|  |  | W092XXD | Fall on or from jungle gym, subsequent encounter |
|  |  | W092XXS | Fall on or from jungle gym, sequela |
|  |  | W098XXA | Fall on or from other playground equipment, init encntr |
|  |  | W098XXD | Fall on or from other playground equipment, subs encntr |
|  |  | W098XXS | Fall on or from other playground equipment, sequela |
|  |  | W100XXA | Fall (on)(from) escalator, initial encounter |
|  |  | W100XXD | Fall (on)(from) escalator, subsequent encounter |
|  |  | W100XXS | Fall (on)(from) escalator, sequela |
|  |  | W101XXA | Fall (on)(from) sidewalk curb, initial encounter |
|  |  | W101XXD | Fall (on)(from) sidewalk curb, subsequent encounter |
|  |  | W101XXS | Fall (on)(from) sidewalk curb, sequela |
|  |  | W102XXA | Fall (on)(from) incline, initial encounter |
|  |  | W102XXD | Fall (on)(from) incline, subsequent encounter |
|  |  | W102XXS | Fall (on)(from) incline, sequela |
|  |  | W108XXA | Fall (on) (from) other stairs and steps, initial encounter |
|  |  | W108XXD | Fall (on) (from) other stairs and steps, subs encntr |
|  |  | W108XXS | Fall (on) (from) other stairs and steps, sequela |
|  |  | W109XXA | Fall (on) (from) unspecified stairs and steps, init encntr |
|  |  | W109XXD | Fall (on) (from) unspecified stairs and steps, subs encntr |
|  |  | W109XXS | Fall (on) (from) unspecified stairs and steps, sequela |
|  |  | W11XXXA | Fall on and from ladder, initial encounter |
|  |  | W11XXXD | Fall on and from ladder, subsequent encounter |
|  |  | W11XXXS | Fall on and from ladder, sequela |
|  |  | W12XXXA | Fall on and from scaffolding, initial encounter |
|  |  | W12XXXD | Fall on and from scaffolding, subsequent encounter |
|  |  | W12XXXS | Fall on and from scaffolding, sequela |
|  |  | W130XXA | Fall from, out of or through balcony, initial encounter |
|  |  | W130XXD | Fall from, out of or through balcony, subsequent encounter |
|  |  | W130XXS | Fall from, out of or through balcony, sequela |
|  |  | W131XXA | Fall from, out of or through bridge, initial encounter |
|  |  | W131XXD | Fall from, out of or through bridge, subsequent encounter |
|  |  | W131XXS | Fall from, out of or through bridge, sequela |
|  |  | W132XXA | Fall from, out of or through roof, initial encounter |
|  |  | W132XXD | Fall from, out of or through roof, subsequent encounter |
|  |  | W132XXS | Fall from, out of or through roof, sequela |
|  |  | W133XXA | Fall through floor, initial encounter |
|  |  | W133XXD | Fall through floor, subsequent encounter |
|  |  | W133XXS | Fall through floor, sequela |
|  |  | W134XXA | Fall from, out of or through window, initial encounter |
|  |  | W134XXD | Fall from, out of or through window, subsequent encounter |
|  |  | W134XXS | Fall from, out of or through window, sequela |
|  |  | W138XXA | Fall from, out of or through oth building or structure, init |
|  |  | W138XXD | Fall from, out of or through oth building or structure, subs |
|  |  | W138XXS | Fall from, out of or through oth bldg, sequela |
|  |  | W139XXA | Fall from, out of or through bldg, not otherwise spcf, init |
|  |  | W139XXD | Fall from, out of or through bldg, not otherwise spcf, subs |
|  |  | W139XXS | Fall from, out of or through bldg, not otherwise spcf, sqla |
|  |  | W14XXXA | Fall from tree, initial encounter |
|  |  | W14XXXD | Fall from tree, subsequent encounter |
|  |  | W14XXXS | Fall from tree, sequela |
|  |  | W15XXXA | Fall from cliff, initial encounter |
|  |  | W15XXXD | Fall from cliff, subsequent encounter |
|  |  | W15XXXS | Fall from cliff, sequela |
|  |  | W16012A | Fall into swimming pool strk surfc causing oth injury, init |
|  |  | W16012D | Fall into swimming pool strk surfc causing oth injury, subs |
|  |  | W16012S | Fall into swim pool strk surfc causing oth injury, sequela |
|  |  | W16022A | Fall into swimming pool strk bottom causing oth injury, init |
|  |  | W16022D | Fall into swimming pool strk bottom causing oth injury, subs |
|  |  | W16022S | Fall into swim pool strk bottom causing oth injury, sequela |
|  |  | W16032A | Fall into swimming pool strk wall causing oth injury, init |
|  |  | W16032D | Fall into swimming pool strk wall causing oth injury, subs |
|  |  | W16032S | Fall into swim pool strk wall causing oth injury, sequela |
|  |  | W16112A | Fall into natrl body of water strk surfc cause oth inj, init |
|  |  | W16112D | Fall into natrl body of water strk surfc cause oth inj, subs |
|  |  | W16112S | Fall into natrl body of water strk surfc cause oth inj, sqla |
|  |  | W16122A | Fall into natrl body of water strk botm cause oth inj, init |
|  |  | W16122D | Fall into natrl body of water strk botm cause oth inj, subs |
|  |  | W16122S | Fall into natrl body of water strk botm cause oth inj, sqla |
|  |  | W16132A | Fall into natrl body of water strk side cause oth inj, init |
|  |  | W16132D | Fall into natrl body of water strk side cause oth inj, subs |
|  |  | W16132S | Fall into natrl body of water strk side cause oth inj, sqla |
|  |  | W16212A | Fall in (into) filled bathtub causing oth injury, init |
|  |  | W16212D | Fall in (into) filled bathtub causing oth injury, subs |
|  |  | W16212S | Fall in (into) filled bathtub causing other injury, sequela |
|  |  | W16222A | Fall in (into) bucket of water causing oth injury, init |
|  |  | W16222D | Fall in (into) bucket of water causing oth injury, subs |
|  |  | W16222S | Fall in (into) bucket of water causing other injury, sequela |
|  |  | W16312A | Fall into oth water striking surfc causing oth injury, init |
|  |  | W16312D | Fall into oth water striking surfc causing oth injury, subs |
|  |  | W16312S | Fall into oth water strk surfc causing oth injury, sequela |
|  |  | W16322A | Fall into oth water striking bottom causing oth injury, init |
|  |  | W16322D | Fall into oth water striking bottom causing oth injury, subs |
|  |  | W16322S | Fall into oth water strk bottom causing oth injury, sequela |
|  |  | W16332A | Fall into oth water striking wall causing oth injury, init |
|  |  | W16332D | Fall into oth water striking wall causing oth injury, subs |
|  |  | W16332S | Fall into oth water strk wall causing oth injury, sequela |
|  |  | W1642XA | Fall into unsp water causing other injury, init encntr |
|  |  | W1642XD | Fall into unsp water causing other injury, subs encntr |
|  |  | W1642XS | Fall into unspecified water causing other injury, sequela |
|  |  | W170XXA | Fall into well, initial encounter |
|  |  | W170XXD | Fall into well, subsequent encounter |
|  |  | W170XXS | Fall into well, sequela |
|  |  | W171XXA | Fall into storm drain or manhole, initial encounter |
|  |  | W171XXD | Fall into storm drain or manhole, subsequent encounter |
|  |  | W171XXS | Fall into storm drain or manhole, sequela |
|  |  | W172XXA | Fall into hole, initial encounter |
|  |  | W172XXD | Fall into hole, subsequent encounter |
|  |  | W172XXS | Fall into hole, sequela |
|  |  | W173XXA | Fall into empty swimming pool, initial encounter |
|  |  | W173XXD | Fall into empty swimming pool, subsequent encounter |
|  |  | W173XXS | Fall into empty swimming pool, sequela |
|  |  | W174XXA | Fall from dock, initial encounter |
|  |  | W174XXD | Fall from dock, subsequent encounter |
|  |  | W174XXS | Fall from dock, sequela |
|  |  | W1781XA | Fall down embankment (hill), initial encounter |
|  |  | W1781XD | Fall down embankment (hill), subsequent encounter |
|  |  | W1781XS | Fall down embankment (hill), sequela |
|  |  | W1782XA | Fall from (out of) grocery cart, initial encounter |
|  |  | W1782XD | Fall from (out of) grocery cart, subsequent encounter |
|  |  | W1782XS | Fall from (out of) grocery cart, sequela |
|  |  | W1789XA | Other fall from one level to another, initial encounter |
|  |  | W1789XD | Other fall from one level to another, subsequent encounter |
|  |  | W1789XS | Other fall from one level to another, sequela |
|  |  | W1811XA | Fall from or off toilet w/o strike against object, init |
|  |  | W1811XD | Fall from or off toilet w/o strike against object, subs |
|  |  | W1811XS | Fall from or off toilet w/o strike against object, sequela |
|  |  | W1812XA | Fall from or off toilet w strike against object, init |
|  |  | W1812XD | Fall from or off toilet w strike against object, subs |
|  |  | W1812XS | Fall from or off toilet w strike against object, sequela |
|  |  | W182XXA | Fall in (into) shower or empty bathtub, initial encounter |
|  |  | W182XXD | Fall in (into) shower or empty bathtub, subsequent encounter |
|  |  | W182XXS | Fall in (into) shower or empty bathtub, sequela |
|  |  | W1830XA | Fall on same level, unspecified, initial encounter |
|  |  | W1830XD | Fall on same level, unspecified, subsequent encounter |
|  |  | W1830XS | Fall on same level, unspecified, sequela |
|  |  | W1831XA | Fall on same level due to stepping on an object, init encntr |
|  |  | W1831XD | Fall on same level due to stepping on an object, subs encntr |
|  |  | W1831XS | Fall on same level due to stepping on an object, sequela |
|  |  | W1839XA | Other fall on same level, initial encounter |
|  |  | W1839XD | Other fall on same level, subsequent encounter |
|  |  | W1839XS | Other fall on same level, sequela |
|  |  | W19XXXA | Unspecified fall, initial encounter |
|  |  | W19XXXD | Unspecified fall, subsequent encounter |
|  |  | W19XXXS | Unspecified fall, sequela |
|  |  | Y30XXXA | Fall, jump or pushed from a high place, undet intent, init |
|  |  | Y30XXXD | Fall, jump or pushed from a high place, undet intent, subs |
|  |  | Y30XXXS | Fall, jump or pushed from a high place, undet intent, sqla |
|  |  | S720xx | Fracture of head and neck of femur |
|  |  | S721xx | Pertrochanteric fracture |
|  |  | S722xx | Subtrochanteric fracture |
|  |  | S723xx | Fracture of shaft of femur |
|  |  | S724xx | Fracture of lower end of femur |
|  |  | S728xx | Other fracture of femur |
|  |  | S729xx | Unspecified fracture of femur |
|  |  | S422xx | Fracture of upper end of humerus |
|  |  | S423xx | Fracture of shaft of humerus |
|  |  | S424xx | Fracture of lower end of humerus |
|  |  | S429xx | Fracture of shoulder girdle, part unspecified part unspecified, |
|  |  | S520xx | Fracture of upper end of ulna |
|  |  | S521xx | Fracture of upper end of radius |
|  |  | S522xx | Fracture of shaft of ulna |
|  |  | S523xx | Fracture of shaft of radius |
|  |  | S525xx | Fracture of lower end of radius |
|  |  | S526xx | Fracture of lower end of ulna |
|  |  | S529xx | Unspecified fracture of forearm |
|  |  | S620xx | Fracture of navicular [scaphoid] bone of wrist |
|  |  | S621xx | Fracture of other and unspecified carpal bone(s), triquetrum, lunate, captiate, hamate, pisiform trapezoid |
|  |  | S820xx | Fracture of patella |
|  |  | S821xx | Fracture of upper end of tibia |
|  |  | S822xx | Fracture of shaft of tibia |
|  |  | S823xx | Fracture of lower end of tibia |
|  |  | S824xx | Fracture of shaft of fibula |
|  |  | S825xx | Fracture of medial malleolus (ankle) |
|  |  | S826xx | Fracture of lateral malleolus |
|  |  | S828xx | Other fractures of lower leg |
|  |  | S829xx | Unspecified fracture of lower leg |
|  |  | S323xx | Fracture of ilium |
|  |  | S324xx | Fracture of acetabulum |
|  |  | S325xx | Fracture of pubis |
|  |  | S326xx | Fracture of ischium |
|  |  | S328xx | Fracture of other parts of pelvis, multiple fractures of pelvis |
|  |  | S329xx | Fracture of unspecified parts of lumbosacral spine and pelvis, NOS |
| **Delirium** | | | |
| 2903 | Senile dementia with delirium/acute confusional state | F05 | Delirium due to known physiological condition |
| 29011 | Presenile dementia with delirium/acute confusional state | F050 | Delirium not superimposed on dementia, so described |
| 29041 | Vascular dementia with delirium/acute confusional state | F051 | Delirium superimposed on dementia |
| 2930 | Delirium due to conditions classified elsewhere | F058 | Other delirium |
| 2931 | Subacute delirium | F059 | Delirium, unspecified |
| 3483 | Encephalopathy, not elsewhere classified | G934 | Other and unspecified encephalopathy |
| 34830 | Encephalopathy, unspecified | G9340 | Encephalopathy, unspecified |
| 34831 | Metabolic encephalopathy | G9341 | Metabolic encephalopathy |
| 34839 | Other encephalopathy | G9349 | Other encephalopathy |
| 34982 | Toxic encephalopathy | G92 | Toxic encephalopathy |
|  |  | R400 | Somnolence |
|  |  | R401 | Stupor |
| **Nausea/vomiting: Claim for antiemetics (based on AHFS classification) after date of analgesic claim, OR any of the following ICD codes** | | | |
| 7870 | Nausea and vomiting | R110 | Nausea |
| 78701 | Nausea with vomiting | R111 | Vomiting |
| 78702 | Nausea alone | R1110 | Vomiting, unspecified |
| 78703 | Vomiting alone | R1111 | Vomiting without nausea |
| 78704 | Bilious emesis | R1112 | Projectile vomiting |
|  |  | R1113 | Vomiting of fecal matter |
|  |  | R1114 | Bilious vomiting |
|  |  | R112 | Nausea with vomiting, unspecified |
| **Slowed colonic motility: Claim for cathartics/laxatives (based on AHFS classification) after date of analgesic claim, OR any of the following ICD codes** | | | |
| 5640 | Constipation | K590 | Constipation |
| 56400 | Constipation, unspecified | K5900 | Constipation unspecified |
| 56401 | Slow transit constipation | K5901 | Slow transit constipation |
| 56402 | Outlet dysfunction constipation | K5902 | Outlet dysfunction constipation |
|  |  | K5903 | Drug induced constipation |
|  |  | K5904 | Chronic idiopathic constipation |
| 56409 | Other constipation | K5909 | Other constipation |
| 56481 | Neurogenic bowel | K592 | Neurogenic bowel, not elsewhere classified |
| 5647 | Megacolon, other than Hirschsprung's (Dilatation of colon) | K5939 | Other megacolon |
| 5601 | Paralytic ileus | K560 | Paralytic ileus |
|  |  | K567 | Ileus, unspecified |
| 56030 | Impaction of colon, unspecified | K564 | Other impaction of intestine |
| 56032 | Fecal impaction | K5641 | Fecal impaction |
| 56039 | Other impaction: Concretion of intestine, enterolith, fecal impaction | K5649 | Other impaction of intestine |
|  |  | K566 | Other and unspecified intestinal obstruction |
|  |  | K5660 | Unspecified intestinal obstruction |
| 5609 | Unspecified intestinal obstruction | K56600 | Partial intestinal obstruction, unspecified as to cause |
|  |  | K56601 | Complete intestinal obstruction, unspecified as to cause |
|  |  | K56609 | unspecified as to partial versus complete obstruction |
| 56089 | Other specified intestinal obstruction | K5669 | Other intestinal obstruction |
|  |  | K56690 | Other partial intestinal obstruction |
|  |  | K56691 | Other complete intenstinal obstruction |
|  |  | K56699 | unspecified as to partial versus complete obstruction |
| **Acute Renal Failure** | | | |
| 584xx | Acute Renal Failure | N17xx | Acute Renal Failure |
| **Gastritis/duodenitis** | | | |
| 53110 | Acute Gastric Ulcer - w perforation wout obstruction | K251 | Acute gastric ulcer with perforation |
| 53111 | Acute Gastric Ulcer - w perforation w obstruction |  |  |
| 53130 | Acute Gastric Ulcer - wout hemorrhage wout perforation wout obstruction | K253 | Acute gastric ulcer without hemorrhage or perforation |
| 53131 | Acute Gastric Ulcer - wout hemorrhage wout perforation w obstruction |  |  |
| 53150 | Chronic or unspecified gastric ulcer with perforation wout obstruction | K255 | Chronic or unspecified gastric ulcer with perforation |
| 53151 | Chronic or unspecified gastric ulcer with perforation w obstruction |  |  |
| 53170 | Chronic wout hemorrhage wout perforation wout obstruction | K257 | Chronic gastric ulcer without hemorrhage or perforation |
| 53171 | Chronic wout hemorrhage wout perforation w obstruction |  |  |
| 53190 | Unspecified as acute or chronic wout hemorrhage wout perforation wout obstruction | K259 | Gastric ulcer, unsp as acute or chronic, w/o hemor or perf |
| 53191 | Unspecified as acute or chronic wout hemorrhage wout perforation w obstruction |  |  |
| 53210 | Acute Duodenal Ulcer - w perforation wout obstruction | K261 | Acute duodenal ulcer with perforation |
| 53211 | Acute Duodenal Ulcer - w perforation w obstruction |  |  |
| 53230 | Acute Duodenal Ulcer - wout hemorrhage wout perforation wout obstruction | K263 | Acute duodenal ulcer without hemorrhage or perforation |
| 53231 | Acute Duodenal Ulcer - wout hemorrhage wout perforation w obstruction |  |  |
| 53250 | Chronic or unspecified duodenal ulcer with perforation wout obstruction | K265 | Chronic or unspecified duodenal ulcer with perforation |
| 53251 | Chronic or unspecified duodenal ulcer with perforation w obstruction |  |  |
| 53270 | Chronic wout hemorrhage wout perforation wout obstruction | K267 | Chronic duodenal ulcer without hemorrhage or perforation |
| 53271 | Chronic wout hemorrhage wout perforation w obstruction |  |  |
| 53290 | Unspecified as acute or chronic wout hemorrhage wout perforation wout obstruction | K269 | Duodenal ulcer, unsp as acute or chronic, w/o hemor or perf |
| 53291 | Unspecified as acute or chronic wout hemorrhage wout perforation w obstruction |  |  |
| 53310 | Acute Peptic Ulcer - w perforation wout obstruction | K271 | Acute peptic ulcer, site unspecified, with perforation |
| 53311 | Acute Peptic Ulcer - w perforation w obstruction |  |  |
| 53330 | Acute Peptic Ulcer - wout hemorrhage wout perforation wout obstruction | K273 | Acute peptic ulcer, site unspecified, without hemorrhage or perforation |
| 53331 | Acute Peptic Ulcer - wout hemorrhage wout perforation w obstruction |  |  |
| 53350 | Chronic or unspecified peptic ulcer with perforation wout obstruction | K275 | Chronic or unsp peptic ulcer, site unsp, with perforation |
| 53351 | Chronic or unspecified peptic ulcer with perforation w obstruction |  |  |
| 53370 | Chronic wout hemorrhage wout perforation wout obstruction | K277 | Chronic peptic ulcer, site unsp, w/o hemorrhage or perf |
| 53371 | Chronic wout hemorrhage wout perforation w obstruction |  |  |
| 53390 | Unspecified as acute or chronic wout hemorrhage wout perforation wout obstruction | K279 | Peptic ulc, site unsp, unsp as ac or chr, w/o hemor or perf |
| 53391 | Unspecified as acute or chronic wout hemorrhage wout perforation w obstruction |  |  |
| 53410 | Acute Gastrojejunal Ulcer - w perforation wout obstruction | K281 | Acute gastrojejunal ulcer with perforation |
| 53411 | Acute Gastrojejunal Ulcer - w perforation w obstruction |  |  |
| 53430 | Acute Gastrojejunal Ulcer - wout hemorrhage wout perforation wout obstruction | K283 | Acute gastrojejunal ulcer without hemorrhage or perforation |
| 53431 | Acute Gastrojejunal Ulcer - wout hemorrhage wout perforation w obstruction |  |  |
| 53450 | Chronic or unspecified gastrojejunal ulcer with perforation wout obstruction | K285 | Chronic or unspecified gastrojejunal ulcer with perforation |
| 53451 | Chronic or unspecified gastrojejunal ulcer with perforation w obstruction |  |  |
| 53470 | Chronic wout hemorrhage wout perforation wout obstruction | K287 | Chronic gastrojejunal ulcer w/o hemorrhage or perforation |
| 53471 | Chronic wout hemorrhage wout perforation w obstruction |  |  |
| 53490 | Unspecified as acute or chronic wout hemorrhage wout perforation wout obstruction | K289 | Gastrojejunal ulcer, unsp as acute or chr, w/o hemor or perf |
| 53491 | Unspecified as acute or chronic wout hemorrhage wout perforation w obstruction |  |  |
| V1271 | Personal history of peptic ulcer disease | Z8711 | Personal history of peptic ulcer disease |
| 53100 | Acute Gastric Ulcer - w hemorrhage wout obstruction | K250 | Acute gastric ulcer with hemorrhage |
| 53101 | Acute Gastric Ulcer - w hemorrhage w obstruction |  |  |
| 53120 | Acute Gastric Ulcer - w hemorrhage w perforation wo obstruction | K252 | Acute gastric ulcer with both hemorrhage and perforation |
| 53121 | Acute Gastric Ulcer - w hemorrhage w perforation w obstruction |  |  |
| 53140 | Chronic or unspecified w hemorrhage wout obstruction | K254 | Chronic or unspecified gastric ulcer with hemorrhage |
| 53141 | Chronic or unspecified w hemorrhage w obstruction |  |  |
| 53160 | Chronic or unspecified w hemorrhage w performation wout obstruction | K256 | Chronic or unsp gastric ulcer w both hemorrhage and perf |
| 53161 | Chronic or unspecified w hemorrhage w performation w obstruction |  |  |
| 53200 | Acute Duodenal Ulcer - w hemorrhage wout obstruction | K260 | Acute duodenal ulcer with hemorrhage |
| 53201 | Acute Duodenal Ulcer - w hemorrhage w obstruction |  |  |
| 53220 | Acute Duodenal Ulcer - w hemorrhage w perforation wo obstruction | K262 | Acute duodenal ulcer with both hemorrhage and perforation |
| 53221 | Acute Duodenal Ulcer - w hemorrhage w perforation w obstruction |  |  |
| 53240 | Chronic or unspecified w hemorrhage wout obstruction | K264 | Chronic or unspecified duodenal ulcer with hemorrhage |
| 53241 | Chronic or unspecified w hemorrhage w obstruction |  |  |
| 53260 | Chronic or unspecified w hemorrhage w performation wout obstruction | K266 | Chronic or unsp duodenal ulcer w both hemorrhage and perf |
| 53261 | Chronic or unspecified w hemorrhage w performation w obstruction |  |  |
| 53300 | Acute Peptic Ulcer - w hemorrhage wout obstruction | K270 | Acute peptic ulcer, site unspecified, with hemorrhage |
| 53301 | Acute Peptic Ulcer - w hemorrhage w obstruction |  |  |
| 53320 | Acute Peptic Ulcer - w hemorrhage w perforation wo obstruction | K272 | Acute peptic ulcer, site unspecified, with both hemorrhage and perforation |
| 53321 | Acute Peptic Ulcer - w hemorrhage w perforation w obstruction |  |  |
| 53340 | Chronic or unspecified w hemorrhage wout obstruction | K274 | Chronic or unsp peptic ulcer, site unsp, with hemorrhage |
| 53341 | Chronic or unspecified w hemorrhage w obstruction |  |  |
| 53360 | Chronic or unspecified w hemorrhage w performation wout obstruction | K276 | Chr or unsp peptic ulcer, site unsp, w both hemor and perf |
| 53361 | Chronic or unspecified w hemorrhage w performation w obstruction |  |  |
| 53400 | Acute Gastrojejunal Ulcer - w hemorrhage wout obstruction | K280 | Acute gastrojejunal ulcer with hemorrhage |
| 53401 | Acute Gastrojejunal Ulcer - w hemorrhage w obstruction |  |  |
| 53420 | Acute Gastrojejunal Ulcer - w hemorrhage w perforation wo obstruction | K282 | Acute gastrojejunal ulcer with both hemorrhage and perforation |
| 53421 | Acute Gastrojejunal Ulcer - w hemorrhage w perforation w obstruction |  |  |
| 53440 | Chronic or unspecified w hemorrhage wout obstruction | K284 | Chronic or unspecified gastrojejunal ulcer with hemorrhage |
| 53441 | Chronic or unspecified w hemorrhage w obstruction |  |  |
| 53460 | Chronic or unspecified w hemorrhage w performation wout obstruction | K286 | Chronic or unsp gastrojejunal ulcer w both hemor and perf |
| 53461 | Chronic or unspecified w hemorrhage w performation w obstruction |  |  |
| 5780 | Hematemesis | K920 | Hematemesis |
| 5781 | Blood in stool - melena | K921 | Melena |
| 5789 | Hemorrhage of gastrointestinal tract, unspecified | K922 | Gastrointestinal hemorrhage, unspecified |
| 5350 | Acute gastritis |  |  |
| 53500 | Acute gastritis without hemorrhage | K2900 | Acute gastritis without bleeding |
| 53501 | Acute gastritis with hemorrhage | K2901 | Acute gastritis with bleeding |
|  |  | K2930 | Chronic superficial gastritis without bleeding |
|  |  | K2931 | Chronic superficial gastritis with bleeding |
|  |  | K2950 | Unspecified chronic gastritis without bleeding |
|  |  | K2951 | Unspecified chronic gastritis with bleeding |
|  |  | K2970 | Gastritis, unspecified, without bleeding |
|  |  | K2971 | Gastritis, unspecified, with bleeding |
|  |  | K2960 | Other gastritis without bleeding |
|  |  | K2961 | Other gastritis with bleeding |
| 5355 | Unspecified gastritis and gastroduodenitis |  |  |
| 53550 | Unspecified gastritis and gastroduodenitis wout hemorrhage | K2990 | Gastroduodenitis, unspecified, without bleeding |
| 53551 | Unspecified gastritis and gastroduodenitis w hemorrhage | K2991 | Gastroduodenitis, unspecified, with bleeding |
| 5356 | Duodenitis |  |  |
| 53560 | Duodenitis wout hemorrhage | K2980 | Duodenitis without bleeding |
| 53561 | Duodenitis w hemorrhage | K2981 | Duodenitis with bleeding |
| **Dementia** | | | |
| 2900 | Senile dementia, uncomplicated | F0151 | Vascular dementia |
| 2901 | Presenile dementia | F0280 | Dementia in other diseases classified elsewhere |
| 290.10 | Presenile uncomp | F0281 | Dementia in other diseases classified elsewhere |
| 290.11 | Presenile dementia with delirium | F0390 | Unspecified dementia |
| 290.12 | Presenile dementia with delusions | F0391 | Unspecified dementia |
| 290.13 | Presenile dementia with depression | F04 | Amnestic disorder due to known physiological condition |
| 290.2 | Senile dementia with delusions or depression | G300 | Alzheimer's disease |
| 290.20 | Senile dementia with delusions | G301 | Alzheimer's disease |
| 290.21 | Senile dementia with depression | G308 | Alzheimer's disease |
| 290.3 | Senile demetia with delirium | G309 | Alzheimer's disease |
| 290.4 | Vascular dementia | G3101 | Pick's disease |
| 290.40 | Vascular dementia uncomp | G3109 | Other frontotemporal dementia |
| 290.41 | Vascular dementia with delirium | G311 | Senile degeneration of brain, not elsewhere classified |
| 290.42 | Vascular dementia with delusions | F1027 | Alcohol dependence with alcohol induced persisting dementia |
| 290.43 | Vascular dementia with depression | F1026 | Alcohol dependence with alcohol induced persisting amnestic disorder |
| 294.0 | Amnestic disorder classified elsewhere (Korsakoffs) | G312 | Degeneration of nervous system due to alcohol |
| 294.1 | Dementia classified elsewhere (any specific etiology) | R4181 | Age-related cognitive decline |
| 294.10 | Dementia classified elsewhere (any specific etiology) without behavioral disturbance | G3183 | Dementia with Lewy Body |
| 294.11 | Dementia classified elsewhere (any specific etiology) with behavioral disturbance | G3184 | Mild Cognitive Impairment |
| 294.2 | Dementia unspecified |  |  |
| 294.20 | Dementia, Unspecified w/o behavioral disturbance |  |  |
| 294.21 | Dementia unspecified |  |  |
| 331.0 | Alzheimer's disease |  |  |
| 331.11 | Pick's Disease |  |  |
| 331.19 | Frontal dementia |  |  |
| 331.2 | Senile degen of brain |  |  |
| 331.7 | Cerebral degen classified elsewhere (etoh, beriberi, stroke etc) |  |  |
| 797 | senility without mention of psychosis |  |  |
| 331.82 | Dementia with lewy body |  |  |
| 331.83 | Mild cognitive impairment |  |  |
| 290.8 | Other non-specified senile psychoses |  |  |
| **ICD*9s and *ICD*10s do not reflect a crosswalk; they are listed side by side in no particular order, to conserve space. | | | |
