# Supplementary material for: Relative risks of adverse events among older adults receiving opioids versus NSAIDs after hospital discharge: A nationwide cohort study
Source: PLoS Med. 2021 Sep 27;18(9):e1003804. doi: 10.1371/journal.pmed.1003804 (PMC8504723; doi:10.1371/journal.pmed.1003804)
Supplement: S3 Table — (DOCX) [file pmed.1003804.s003.docx]

| **S3 Table. Characteristics of study population, before and after propensity matching (see Appendix Figure 1 for standardized mean differences; all were <0.1 after the match).** | | | | | | | | | | |
| --- | --- | --- | --- | --- | --- | --- | --- | --- | --- | --- |
|  | | | **Before Propensity Matching** | | | | **After Propensity Matching** | | | |
|  | | | **Opioid** | | **NSAID** | | **Opioid** | | **NSAID** | |
| **Characteristic – n % unless otherwise noted** | | | n=111,061 | | n=4,713 | | n=13,385 | | n=4,677 | |
| Age in years – mean, s.d. | | | 74.3 | 6.5 | 75.9 | 7.5 | 75.8 | 7.5 | 75.9 | 7.5 |
| Male | | | 47414 | 42.7 | 1703 | 36.1 | 4840 | 36.2 | 1689 | 36.1 |
| Race | | |  |  |  |  |  |  |  |  |
|  | Black | | 8483 | 7.6 | 494 | 10.5 | 1467 | 11.0 | 489 | 10.5 |
|  | White | | 96776 | 87.1 | 3742 | 79.4 | 10646 | 79.5 | 3719 | 79.5 |
|  | Other | | 5802 | 5.2 | 477 | 10.1 | 1272 | 9.5 | 469 | 10.0 |
| Original reason for entitlement | | |  |  |  |  |  |  |  |  |
|  | Age | | 91047 | 82.0 | 3649 | 77.4 | 10289 | 76.9 | 3623 | 77.5 |
|  | Disability/ESRD | | 20014 | 18.0 | 1064 | 22.6 | 3096 | 23.1 | 1054 | 22.5 |
| Medicaid dual eligible | | | 21186 | 19.1 | 1694 | 35.9 | 4636 | 34.6 | 1666 | 35.6 |
| Prior diagnoses | | |  |  |  |  |  |  |  |  |
|  | Congestive heart failure | | 23398 | 21.1 | 1165 | 24.7 | 3481 | 26.0 | 1158 | 24.8 |
|  | Cardiac arrhythmias | | 37261 | 33.6 | 1657 | 35.2 | 4846 | 36.2 | 1643 | 35.1 |
|  | Valvular disease | | 17746 | 16.0 | 752 | 16.0 | 2231 | 16.7 | 750 | 16.0 |
|  | Pulmonary circulation disorders | | 8724 | 7.9 | 374 | 7.9 | 1153 | 8.6 | 373 | 8.0 |
|  | Peripheral vascular disorders | | 24084 | 21.7 | 1048 | 22.2 | 3071 | 22.9 | 1042 | 22.3 |
|  | Hypertension, uncomplicated | | 92579 | 83.4 | 4046 | 85.8 | 11477 | 85.7 | 4016 | 85.9 |
|  | Hypertension, complicated | | 25411 | 22.9 | 1002 | 21.3 | 2986 | 22.3 | 1000 | 21.4 |
|  | Paralysis | | 1685 | 1.5 | 113 | 2.4 | 306 | 2.3 | 112 | 2.4 |
|  | Other neurological disorders | | 10326 | 9.3 | 708 | 15.0 | 1949 | 14.6 | 692 | 14.8 |
|  | Chronic pulmonary disease | | 36454 | 32.8 | 1826 | 38.7 | 5188 | 38.8 | 1813 | 38.8 |
|  | Diabetes, uncomplicated | | 36860 | 33.2 | 1737 | 36.9 | 4932 | 36.8 | 1719 | 36.8 |
|  | Diabetes, complicated | | 23289 | 21.0 | 1085 | 23.0 | 3110 | 23.2 | 1076 | 23.0 |
|  | Hypothyroidism | | 27075 | 24.4 | 1207 | 25.6 | 3424 | 25.6 | 1197 | 25.6 |
|  | Renal failure | | 24984 | 22.5 | 881 | 18.7 | 2670 | 19.9 | 881 | 18.8 |
|  | Liver disease | | 6903 | 6.2 | 266 | 5.6 | 791 | 5.9 | 263 | 5.6 |
|  |  | | **Before Propensity Matching** | | | | **After Propensity Matching** | | | |
|  |  | | **Opioid** | | **NSAID** | | **Opioid** | | **NSAID** | |
|  | AIDS/HIV | | 192 | 0.2 | -^a^ | -^a^ | 39 | 0.3 | -^a^ | -^a^ |
|  | Lymphoma | | 2155 | 1.9 | 73 | 1.5 | 221 | 1.7 | 73 | 1.6 |
|  | Metastatic cancer | | 6793 | 6.1 | 195 | 4.1 | 636 | 4.8 | 195 | 4.2 |
|  | Solid tumor without metastasis | | 22971 | 20.7 | 714 | 15.1 | 2127 | 15.9 | 711 | 15.2 |
|  | Rheumatoid arthritis/collagen vascular diseases | | 10749 | 9.7 | 551 | 11.7 | 1520 | 11.4 | 548 | 11.7 |
|  | Coagulopathy | | 9928 | 8.9 | 357 | 7.6 | 1090 | 8.1 | 357 | 7.6 |
|  | Obesity | | 26002 | 23.4 | 1014 | 21.5 | 2951 | 22.0 | 1007 | 21.5 |
|  | Weight loss | | 9084 | 8.2 | 374 | 7.9 | 1157 | 8.6 | 374 | 8.0 |
|  | Fluid and electrolyte disorders | | 35789 | 32.2 | 1868 | 39.6 | 5455 | 40.8 | 1853 | 39.6 |
|  | Blood loss anemia | | 3276 | 2.9 | 117 | 2.5 | 372 | 2.8 | 116 | 2.5 |
|  | Deficiency anemia | | 13771 | 12.4 | 590 | 12.5 | 1789 | 13.4 | 588 | 12.6 |
|  | Alcohol abuse | | 3446 | 3.1 | 189 | 4.0 | 510 | 3.8 | 187 | 4.0 |
|  | Psychoses | | 1398 | 1.3 | 211 | 4.5 | 479 | 3.6 | 195 | 4.2 |
|  | Depression | | 25269 | 22.8 | 1312 | 27.8 | 3718 | 27.8 | 1297 | 27.7 |
|  | Osteoporosis | | 9546 | 8.6 | 462 | 9.8 | 1380 | 10.3 | 459 | 9.8 |
|  | Migraine and chronic headache | | 2972 | 2.7 | 172 | 3.6 | 469 | 3.5 | 170 | 3.6 |
|  | Bipolar disorder | | 1989 | 1.8 | 151 | 3.2 | 416 | 3.1 | 148 | 3.2 |
|  | Anxiety disorder | | 21766 | 19.6 | 1098 | 23.3 | 3101 | 23.2 | 1086 | 23.2 |
|  | Opioid use disorder | | 10301 | 9.3 | 414 | 8.8 | 1287 | 9.6 | 410 | 8.8 |
|  | Drug use disorder | | 2081 | 1.9 | 135 | 2.9 | 380 | 2.8 | 134 | 2.9 |
|  | Dementia | | 5826 | 5.2 | 581 | 12.8 | 1524 | 11.4 | 571 | 12.2 |
|  | Falls/fractures | | 56 | 0.1 | -^a^ | -^a^ | 16 | 0.1 | -^a^ | -^a^ |
|  | Delirium | | 6020 | 5.4 | 412 | 8.7 | 1178 | 8.8 | 405 | 8.7 |
|  | Nausea/vomiting | | 24102 | 21.7 | 985 | 20.9 | 2868 | 21.4 | 978 | 20.9 |
|  | Slowed colonic motility^b^ | | 25871 | 23.3 | 1062 | 22.5 | 3116 | 23.3 | 1051 | 22.5 |
|  | Acute renal failure | | 17516 | 15.8 | 816 | 17.3 | 2465 | 18.4 | 810 | 17.3 |
|  | Gastritis/duodenitis^c^ | | 9655 | 8.7 | 393 | 8.3 | 1222 | 9.1 | 392 | 8.4 |
| Frailty/function | | |  |  |  |  |  |  |  |  |
|  | Frailty Index – mean, s.d. | | 0.2 | 0.1 | 0.2 | 0.1 | 0.2 | 0.1 | 0.2 | 0.1 |
|  |  | | **Before Propensity Matching** | | | | **After Propensity Matching** | | | |
|  |  | | **Opioid** | | **NSAID** | | **Opioid** | | **NSAID** | |
|  | Home healthcare claims | | 20848 | 18.8 | 1239 | 26.3 | 3593 | 26.8 | 1225 | 26.2 |
|  | Skilled nursing facility claims | | 6491 | 5.8 | 315 | 6.7 | 963 | 7.2 | 312 | 6.7 |
|  | Mobility impairment | | 2832 | 2.5 | 167 | 3.5 | 469 | 3.5 | 166 | 3.5 |
| Hospitalization characteristics | | |  |  |  |  |  |  |  |  |
|  | Length of stay – mean, s.d. | | 3.7 | 3.7 | 3.7 | 4.4 | 3.7 | 3.4 | 3.7 | 4.3 |
|  | Any time in intensive care | | 22629 | 20.4 | 1045 | 22.2 | 2945 | 22.0 | 1036 | 22.2 |
|  | Diagnosis-related group | |  |  |  |  |  |  |  |  |
|  |  | Medical | 36789 | 33.1 | 3438 | 73.0 | 9665 | 72.2 | 3402 | 72.7 |
|  |  | Surgical | 74272 | 66.9 | 1275 | 27.1 | 3720 | 27.8 | 1275 | 27.3 |
| Primary discharge diagnosis | | |  |  |  |  |  |  |  |  |
|  | Infectious and parasitic diseases | | 3423 | 3.1 | 275 | 5.8 | 782 | 5.8 | 272 | 5.8 |
|  | Neoplasms | | 11303 | 10.2 | 209 | 4.4 | 674 | 5.0 | 209 | 4.5 |
|  | Endocrine; nutritional; and metabolic diseases and immunity disorders | | 2315 | 2.1 | 191 | 4.1 | 541 | 4.0 | 190 | 4.1 |
|  | Diseases of the blood and blood-forming organs | | 605 | 0.5 | 48 | 1.0 | 145 | 1.1 | 48 | 1.0 |
|  | Mental illness | | 594 | 0.5 | 201 | 4.3 | 413 | 3.1 | 185 | 4.0 |
|  | Diseases of the nervous system and sense organs | | 1594 | 1.4 | 150 | 3.2 | 436 | 3.3 | 150 | 3.2 |
|  | Diseases of the circulatory system | | 16604 | 15.0 | 963 | 20.4 | 2678 | 20.0 | 958 | 20.5 |
|  | Diseases of the respiratory system | | 5726 | 5.2 | 625 | 13.3 | 1740 | 13.0 | 618 | 13.2 |
|  | Diseases of the digestive system | | 12708 | 11.4 | 498 | 10.6 | 1458 | 10.9 | 496 | 10.6 |
|  | Diseases of the genitourinary system | | 5022 | 4.5 | 350 | 7.4 | 1034 | 7.7 | 348 | 7.4 |
|  | Diseases of the skin and subcutaneous tissue | | 1800 | 1.6 | 138 | 2.9 | 428 | 3.2 | 138 | 3.0 |
|  | Diseases of the musculoskeletal system and connective tissue | | 36243 | 32.6 | 621 | 13.2 | 1734 | 13.0 | 621 | 13.3 |
|  | Injury and poisoning | | 11350 | 10.2 | 336 | 7.1 | 1009 | 7.5 | 336 | 7.2 |
|  | Symptoms; signs; and ill-defined conditions and factors influencing health status | | 1430 | 1.3 | 93 | 2.0 | 261 | 1.9 | 93 | 2.0 |
|  | Residual codes; unclassified; all E codes | | 161 | 0.1 | 15 | 0.3 | 49 | 0.4 | 15 | 0.3 |
| Primary discharge procedure | | |  |  |  |  |  |  |  |  |
|  | Operations on the nervous system | | 3225 | 2.9 | 65 | 1.4 | 200 | 1.4 | 65 | 1.4 |
|  |  | | **Before Propensity Matching** | | | | **After Propensity Matching** | | | |
|  |  | | **Opioid** | | **NSAID** | | **Opioid** | | **NSAID** | |
|  | Operations on the endocrine system | | 330 | 0.3 | -^a^ | -^a^ | 20 | 0.2 | -^a^ | -^a^ |
|  | Operations on the eye | | 37 | 0.0 | -^a^ | -^a^ | -^a^ | -^a^ | -^a^ | -^a^ |
|  | Operations on the ear | | 85 | 0.1 | -^a^ | -^a^ | -^a^ | -^a^ | -^a^ | -^a^ |
|  | Operations on the nose, mouth, and pharynx | | 349 | 0.3 | -^a^ | -^a^ | 30 | 0.2 | -^a^ | -^a^ |
|  | Operations on the respiratory system | | 3701 | 3.3 | 110 | 2.3 | 310 | 2.3 | 110 | 2.4 |
|  | Operations on the cardiovascular system | | 13415 | 12.1 | 446 | 9.5 | 1314 | 9.8 | 445 | 9.5 |
|  | Operations on the hemic and lymphatic system | | 686 | 0.6 | -^a^ | -^a^ | 35 | 0.3 | -^a^ | -^a^ |
|  | Operations on the digestive system | | 14884 | 13.4 | 365 | 7.7 | 1107 | 8.3 | 364 | 7.8 |
|  | Operations on the urinary system | | 3080 | 2.8 | 53 | 1.1 | 164 | 1.2 | 53 | 1.1 |
|  | Operations on the male genital organs | | 1511 | 1.4 | 17 | 0.4 | 63 | 0.5 | 17 | 0.4 |
|  | Operations on the female genital organs | | 1259 | 1.1 | 99 | 2.1 | 330 | 2.5 | 99 | 2.1 |
|  | Operations on the musculoskeletal system | | 38892 | 35.0 | 617 | 13.1 | 1724 | 12.9 | 617 | 13.2 |
|  | Operations on the integumentary system | | 2172 | 2.0 | 77 | 1.6 | 226 | 1.7 | 77 | 1.6 |
|  | Miscellaneous diagnostic and therapeutic procedures | | 4951 | 4.5 | 454 | 9.6 | 1249 | 9.3 | 447 | 9.6 |
| Number of prior hospitalizations – mean, s.d. | | | 0.7 | 1.5 | 0.8 | 1.5 | 0.9 | 1.6 | 0.8 | 1.5 |
| Medication use in prior 90d | | |  |  |  |  |  |  |  |  |
|  | Number of claims – mean, s.d. | | 12.2 | 9.8 | 16.5 | 12.8 | 16.1 | 12.6 | 16.4 | 12.6 |
|  | Benzodiazepines | | 20378 | 18.3 | 1015 | 21.5 | 2832 | 21.2 | 1003 | 21.4 |
|  | Muscle relaxants | | 6642 | 6.0 | 366 | 7.8 | 1050 | 7.8 | 364 | 7.8 |
|  | Stimulants | | 615 | 0.6 | 26 | 0.6 | 68 | 0.5 | 26 | 0.6 |
|  | Zolpidem | | 5141 | 4.6 | 237 | 5.0 | 716 | 5.3 | 233 | 5.0 |
|  | Antidepressants | | 32301 | 29.1 | 1662 | 35.3 | 4579 | 34.2 | 1634 | 34.9 |
|  | Antipsychotics | | 4131 | 3.7 | 369 | 7.8 | 950 | 7.1 | 353 | 7.5 |
|  | Diuretics | | 41623 | 37.5 | 1885 | 40.0 | 5400 | 40.3 | 1866 | 39.9 |
|  | ACE-I/ARBs | | 47265 | 42.6 | 2155 | 45.7 | 5971 | 44.6 | 2134 | 45.6 |
|  | Acid-suppressive medications | | 36959 | 33.3 | 1968 | 41.8 | 5483 | 41.0 | 1943 | 41.5 |
| Medication use within 7d of discharge | | |  |  |  |  |  |  |  |  |
|  | Number of claims – mean, s.d. | | 3.3 | 2.4 | 4.6 | 3.4 | 4.4 | 3.2 | 4.5 | 3.4 |
|  | Benzodiazepines | | 7715 | 6.9 | 384 | 8.1 | 1090 | 8.1 | 378 | 8.1 |
|  |  | | **Before Propensity Matching** | | | | **After Propensity Matching** | | | |
|  |  | | **Opioid** | | **NSAID** | | **Opioid** | | **NSAID** | |
|  | Muscle relaxants | | 3587 | 3.2 | 138 | 2.9 | 370 | 2.8 | 138 | 3.0 |
|  | Stimulants | | 140 | 0.1 | -^a^ | -^a^ | 16 | 0.1 | -^a^ | -^a^ |
|  | Zolpidem | | 1317 | 1.2 | 62 | 1.3 | 168 | 1.3 | 61 | 1.3 |
|  | Antidepressants | | 7254 | 6.5 | 776 | 16.5 | 1927 | 14.4 | 746 | 16.0 |
|  | Antipsychotics | | 1638 | 1.5 | 235 | 5.0 | 527 | 3.9 | 219 | 4.7 |
|  | Diuretics | | 11716 | 10.5 | 812 | 17.2 | 2164 | 16.2 | 798 | 17.1 |
|  | ACE-I/ARBs | | 9302 | 8.4 | 866 | 18.4 | 2277 | 17.0 | 845 | 18.1 |
|  | Acid-suppressive medications | | 11728 | 10.6 | 991 | 21.0 | 2610 | 19.5 | 962 | 20.6 |
| Prior high-dose long-term opioid use^d^ | | | 3617 | 3.3 | 113 | 2.4 | 417 | 3.1 | 113 | 2.4 |
| Abbreviations: ACE-I/ARB = angiotensin converting enzyme inhibitor/angiotensin receptor blocker; d = days; ESRD = end-stage renal disease; HIV/AIDS = human immunodeficiency virus/acquired immunodeficiency virus; NSAID = non-steroidal anti-inflammatory drug; s.d. = standard deviation | | | | | | | | | | |
| ^a^ Cell suppressed owing to small cell size, in accordance with CMS policy | | | | | | | | | | |
| ^b^ Includes constipation, ileus, impaction, obstruction | | | | | | | | | | |
| ^c^ Includes gastric or duodenal inflammation or ulcer, or upper-gastrointestinal bleeding | | | | | | | | | | |
| ^d^ Defined as average daily morphine equivalents greater than 120 mg for any 90-day period and at least 90 days of opioid use in the year prior to hospitalization | | | | | | | | | | |
