# Supplementary material for: Relative risks of adverse events among older adults receiving opioids versus NSAIDs after hospital discharge: A nationwide cohort study
Source: PLoS Med. 2021 Sep 27;18(9):e1003804. doi: 10.1371/journal.pmed.1003804 (PMC8504723; doi:10.1371/journal.pmed.1003804)
Supplement: S4 Table — Characteristics of study population, before and after propensity matching. (DOCX) [file pmed.1003804.s004.docx]

| **S4 Table. Subgroup analysis in beneficiaries without opioid or NSAID claims in the 90 days prior to hospitalization. Characteristics of study population, before and after propensity matching (see Appendix Figure 1 for standardized mean differences; all <0.1 after the match).** | | | | | | | | | | |
| --- | --- | --- | --- | --- | --- | --- | --- | --- | --- | --- |
|  | | | **Before Propensity Matching** | | | | **After Propensity Matching** | | | |
|  | | | **Opioid** | | **NSAID** | | **Opioid** | | **NSAID** | |
| **Characteristic – n % unless otherwise noted** | | | n=51,964 | | n=1,454 | | n=4,151 | | n=1,431 | |
| Age in years – mean, s.d. | | | 74.6 | 6.4 | 76.3 | 7.5 | 76.5 | 7.6 | 76.3 | 7.5 |
| Male | | | 24413 | 47.0 | 569 | 39.1 | 1637 | 39.4 | 558 | 39.0 |
| Race | | |  |  |  |  |  |  |  |  |
|  | Black | | 3214 | 6.2 | 144 | 9.9 | 416 | 10.0 | 136 | 9.5 |
|  | White | | 45796 | 88.1 | 1164 | 80.1 | 3336 | 80.4 | 1151 | 80.4 |
|  | Other | | 2954 | 5.7 | 146 | 10.0 | 399 | 9.6 | 144 | 10.1 |
| Original reason for entitlement | | |  |  |  |  |  |  |  |  |
|  | Age | | 46045 | 88.6 | 1232 | 84.7 | 3554 | 85.6 | 1215 | 84.9 |
|  | Disability/ESRD | | 5919 | 11.4 | 222 | 15.3 | 597 | 14.4 | 216 | 15.1 |
| Medicaid dual eligible | | | 6665 | 12.8 | 389 | 26.8 | 1022 | 24.6 | 378 | 26.4 |
| Prior diagnoses | | |  |  |  |  |  |  |  |  |
|  | Congestive heart failure | | 8979 | 17.3 | 290 | 19.9 | 867 | 20.9 | 284 | 19.8 |
|  | Cardiac arrhythmias | | 16797 | 32.3 | 497 | 34.2 | 1480 | 35.7 | 491 | 34.3 |
|  | Valvular disease | | 8528 | 16.4 | 232 | 16.0 | 705 | 17.0 | 228 | 15.9 |
|  | Pulmonary circulation disorders | | 3357 | 6.5 | 87 | 6.0 | 262 | 6.3 | 85 | 5.9 |
|  | Peripheral vascular disorders | | 10175 | 19.6 | 300 | 20.6 | 887 | 21.4 | 299 | 20.9 |
|  | Hypertension, uncomplicated | | 41938 | 80.7 | 1189 | 81.8 | 3374 | 81.3 | 1170 | 81.8 |
|  | Hypertension, complicated | | 10375 | 20.0 | 245 | 16.9 | 741 | 17.9 | 244 | 17.1 |
|  | Paralysis | | 658 | 1.3 | 24 | 1.7 | 68 | 1.6 | 24 | 1.7 |
|  | Other neurological disorders | | 3572 | 6.9 | 173 | 11.9 | 492 | 11.9 | 167 | 11.7 |
|  | Chronic pulmonary disease | | 13740 | 26.4 | 460 | 31.6 | 1295 | 31.2 | 455 | 31.8 |
|  | Diabetes, uncomplicated | | 15564 | 30.0 | 451 | 31.0 | 1277 | 30.8 | 444 | 31.0 |
|  | Diabetes, complicated | | 9274 | 17.8 | 272 | 18.7 | 783 | 18.9 | 270 | 18.9 |
|  | Hypothyroidism | | 11765 | 22.6 | 336 | 23.1 | 947 | 22.8 | 333 | 23.3 |
|  | Renal failure | | 10323 | 19.9 | 224 | 15.4 | 702 | 16.9 | 223 | 15.6 |
|  |  | | **Before Propensity Matching** | | | | **After Propensity Matching** | | | |
|  |  | | **Opioid** | | **NSAID** | | **Opioid** | | **NSAID** | |
|  | Liver disease | | 2836 | 5.5 | 89 | 6.1 | 256 | 6.2 | 85 | 5.9 |
|  | AIDS/HIV | | 51 | 0.1 | - ^a^ | - ^a^ | 10 | 0.2 | - ^a^ | - ^a^ |
|  | Lymphoma | | 879 | 1.7 | 17 | 1.2 | 60 | 1.4 | 17 | 1.2 |
|  | Metastatic cancer | | 2815 | 5.4 | 72 | 5.0 | 245 | 5.9 | 72 | 5.0 |
|  | Solid tumor without metastasis | | 11504 | 22.1 | 252 | 17.3 | 786 | 18.9 | 249 | 17.4 |
|  | Rheumatoid arthritis/collagen vascular diseases | | 3310 | 6.4 | 110 | 7.6 | 313 | 7.5 | 107 | 7.5 |
|  | Coagulopathy | | 4472 | 8.6 | 100 | 6.9 | 309 | 7.4 | 98 | 6.8 |
|  | Obesity | | 10823 | 20.8 | 244 | 16.8 | 720 | 17.3 | 240 | 16.8 |
|  | Weight loss | | 3193 | 6.1 | 102 | 7.0 | 331 | 8.0 | 100 | 7.0 |
|  | Fluid and electrolyte disorders | | 13775 | 26.5 | 478 | 32.9 | 1364 | 32.9 | 467 | 32.6 |
|  | Blood loss anemia | | 1326 | 2.6 | 40 | 2.8 | 128 | 3.1 | 40 | 2.8 |
|  | Deficiency anemia | | 5179 | 10.0 | 145 | 10.0 | 438 | 10.6 | 143 | 10.0 |
|  | Alcohol abuse | | 1309 | 2.5 | 56 | 3.9 | 142 | 3.4 | 48 | 3.4 |
|  | Psychoses | | 401 | 0.8 | 47 | 3.2 | 87 | 2.1 | 39 | 2.7 |
|  | Depression | | 8357 | 16.1 | 301 | 20.7 | 834 | 20.1 | 286 | 20.0 |
|  | Osteoporosis | | 3754 | 7.2 | 128 | 8.8 | 375 | 9.0 | 127 | 8.9 |
|  | Migraine and chronic headache | | 955 | 1.8 | 43 | 3.0 | 131 | 3.2 | 42 | 2.9 |
|  | Bipolar disorder | | 559 | 1.1 | 33 | 2.3 | 82 | 2.0 | 29 | 2.0 |
|  | Anxiety disorder | | 7031 | 13.5 | 262 | 18.0 | 742 | 17.9 | 251 | 17.5 |
|  | Opioid use disorder | | 3367 | 6.5 | 88 | 6.1 | 276 | 6.6 | 85 | 5.9 |
|  | Drug use disorder | | 508 | 1.0 | 34 | 2.3 | 85 | 2.0 | 25 | 1.7 |
|  | Dementia | | 2150 | 4.1 | 153 | 10.5 | 400 | 9.6 | 147 | 10.3 |
|  | Falls/fractures | | 22 | 0.0 | - ^a^ | - ^a^ | - ^a^ | - ^a^ | - ^a^ | - ^a^ |
|  | Delirium | | 1637 | 3.2 | 86 | 5.9 | 238 | 5.7 | 82 | 5.7 |
|  | Nausea/vomiting | | 8786 | 16.9 | 234 | 16.1 | 711 | 17.1 | 231 | 16.1 |
|  | Constipation/ileus/impaction/obstruction | | 10509 | 20.2 | 273 | 18.8 | 764 | 18.4 | 270 | 18.9 |
|  | Acute renal failure | | 6569 | 12.6 | 177 | 12.2 | 556 | 13.4 | 175 | 12.2 |
|  | Upper gastrointestinal inflammation/ulcer/bleeding | | 3477 | 6.7 | 91 | 6.3 | 297 | 7.2 | 91 | 6.4 |
| Frailty/function | | |  |  |  |  |  |  |  |  |
|  |  | | **Before Propensity Matching** | | | | **After Propensity Matching** | | | |
|  |  | | **Opioid** | | **NSAID** | | **Opioid** | | **NSAID** | |
|  | Frailty Index – mean, s.d. | | 0.2 | 0.1 | 0.2 | 0.1 | 0.2 | 0.1 | 0.2 | 0.1 |
|  | Home healthcare claims | | 6611 | 12.7 | 269 | 18.5 | 771 | 18.6 | 265 | 18.5 |
|  | Skilled nursing facility claims | | 1905 | 3.7 | 78 | 5.4 | 242 | 5.8 | 75 | 5.2 |
|  | Mobility impairment | | 1094 | 2.1 | 37 | 2.5 | 115 | 2.8 | 37 | 2.6 |
| Hospitalization characteristics | | |  |  |  |  |  |  |  |  |
|  | Length of stay – mean, s.d. | | 3.7 | 3.6 | 3.7 | 5.8 | 3.8 | 4.5 | 3.6 | 5.5 |
|  | Any time in intensive care | | 11342 | 21.8 | 315 | 21.7 | 910 | 21.9 | 313 | 21.9 |
|  | Diagnosis-related group | |  |  |  |  |  |  |  |  |
|  |  | Medical | 11749 | 22.6 | 946 | 65.1 | 2668 | 64.3 | 923 | 64.5 |
|  |  | Surgical | 40215 | 77.4 | 508 | 34.9 | 1483 | 35.7 | 508 | 35.5 |
| Primary discharge diagnosis | | |  |  |  |  |  |  |  |  |
|  | Infectious and parasitic diseases | | 1048 | 2.0 | 59 | 4.1 | 183 | 4.4 | 59 | 4.1 |
|  | Neoplasms | | 6963 | 13.4 | 112 | 7.7 | 359 | 8.6 | 112 | 7.8 |
|  | Endocrine; nutritional; and metabolic diseases and immunity disorders | | 839 | 1.6 | 59 | 4.1 | 160 | 3.9 | 58 | 4.1 |
|  | Diseases of the blood and blood-forming organs | | 182 | 0.4 | 15 | 1.0 | 38 | 0.9 | 15 | 1.0 |
|  | Mental illness | | 103 | 0.2 | 50 | 3.4 | 73 | 1.8 | 33 | 2.3 |
|  | Diseases of the nervous system and sense organs | | 548 | 1.1 | 46 | 3.2 | 133 | 3.2 | 44 | 3.1 |
|  | Diseases of the circulatory system | | 8595 | 16.5 | 318 | 21.9 | 875 | 21.1 | 316 | 22.1 |
|  | Diseases of the respiratory system | | 1578 | 3.0 | 149 | 10.2 | 419 | 10.1 | 148 | 10.3 |
|  | Diseases of the digestive system | | 6520 | 12.5 | 141 | 9.7 | 406 | 9.8 | 141 | 9.9 |
|  | Diseases of the genitourinary system | | 2091 | 4.0 | 105 | 7.2 | 333 | 8.0 | 105 | 7.3 |
|  | Diseases of the skin and subcutaneous tissue | | 672 | 1.3 | 35 | 2.4 | 111 | 2.7 | 35 | 2.4 |
|  | Diseases of the musculoskeletal system and connective tissue | | 16887 | 32.5 | 231 | 15.9 | 615 | 14.8 | 231 | 16.1 |
|  | Injury and poisoning | | 5290 | 10.2 | 91 | 6.3 | 314 | 7.6 | 91 | 6.4 |
|  | Symptoms; signs; and ill-defined conditions and factors influencing health status | | 497 | 1.0 | 39 | 2.7 | 113 | 2.7 | 39 | 2.7 |
|  | Residual codes; unclassified; all E codes | | 35 | 0.1 | - ^a^ | - ^a^ | 18 | 0.4 | - ^a^ | - ^a^ |
| Primary discharge procedure | | |  |  |  |  |  |  |  |  |
|  |  | | **Before Propensity Matching** | | | | **After Propensity Matching** | | | |
|  |  | | **Opioid** | | **NSAID** | | **Opioid** | | **NSAID** | |
|  | Operations on the nervous system | | 1390 | 2.7 | 22 | 1.5 | 71 | 1.7 | 21 | 1.5 |
|  | Operations on the endocrine system | | 214 | 0.4 | - ^a^ | - ^a^ | 12 | 0.3 | - ^a^ | - ^a^ |
|  | Operations on the eye | | 18 | 0.0 | - ^a^ | - ^a^ | - ^a^ | - ^a^ | - ^a^ | - ^a^ |
|  | Operations on the ear | | 45 | 0.1 | - ^a^ | - ^a^ | - ^a^ | - ^a^ | - ^a^ | - ^a^ |
|  | Operations on the nose, mouth, and pharynx | | 159 | 0.3 | - ^a^ | - ^a^ | 19 | 0.5 | - ^a^ | - ^a^ |
|  | Operations on the respiratory system | | 2157 | 4.2 | 37 | 2.5 | 108 | 2.6 | 37 | 2.6 |
|  | Operations on the cardiovascular system | | 7782 | 15.0 | 159 | 10.9 | 442 | 10.6 | 158 | 11.0 |
|  | Operations on the hemic and lymphatic system | | 367 | 0.7 | - ^a^ | - ^a^ | 12 | 0.3 | - ^a^ | - ^a^ |
|  | Operations on the digestive system | | 8462 | 16.3 | 133 | 9.1 | 405 | 9.8 | 133 | 9.3 |
|  | Operations on the urinary system | | 1716 | 3.3 | 12 | 0.8 | 48 | 1.2 | 12 | 0.8 |
|  | Operations on the male genital organs | | 1088 | 2.1 | - ^a^ | - ^a^ | 21 | 0.5 | - ^a^ | - ^a^ |
|  | Operations on the female genital organs | | 781 | 1.5 | 69 | 4.7 | 223 | 5.4 | 69 | 4.8 |
|  | Operations on the musculoskeletal system | | 18388 | 35.4 | 223 | 15.3 | 616 | 14.8 | 222 | 15.5 |
|  | Operations on the integumentary system | | 969 | 1.9 | 31 | 2.1 | 96 | 2.3 | 31 | 2.2 |
|  | Miscellaneous diagnostic and therapeutic procedures | | 1593 | 3.1 | 126 | 8.7 | 360 | 8.7 | 119 | 8.3 |
| Number of prior hospitalizations – mean, s.d. | | | 0.5 | 1.1 | 0.7 | 1.4 | 0.6 | 1.3 | 0.6 | 1.3 |
| Medication use in prior 90d | | |  |  |  |  |  |  |  |  |
|  | Number of claims – mean, s.d. | | 8.3 | 7.0 | 9.2 | 8.4 | 9.3 | 8.1 | 9.2 | 8.4 |
|  | Benzodiazepines | | 5780 | 11.1 | 181 | 12.4 | 497 | 12.0 | 176 | 12.3 |
|  | Muscle relaxants | | 1005 | 1.9 | 31 | 2.1 | 93 | 2.2 | 31 | 2.2 |
|  | Stimulants | | 173 | 0.3 | - ^a^ | - ^a^ | 16 | 0.4 | - ^a^ | - ^a^ |
|  | Zolpidem | | 1493 | 2.9 | 33 | 2.3 | 118 | 2.8 | 33 | 2.3 |
|  | Antidepressants | | 10762 | 20.7 | 310 | 21.3 | 906 | 21.8 | 300 | 21.0 |
|  | Antipsychotics | | 1067 | 2.1 | 79 | 5.4 | 183 | 4.4 | 73 | 5.1 |
|  | Diuretics | | 17481 | 33.6 | 450 | 30.9 | 1295 | 31.2 | 448 | 31.3 |
|  | ACE-I/ARBs | | 21730 | 41.8 | 577 | 39.7 | 1658 | 39.9 | 569 | 39.8 |
|  | Acid-suppressive medications | | 13476 | 25.9 | 425 | 29.2 | 1182 | 28.5 | 419 | 29.3 |
| Medication use within 7d of discharge | | |  |  |  |  |  |  |  |  |
|  | Number of claims – mean, s.d. | | 3.0 | 2.0 | 3.6 | 2.6 | 3.5 | 2.4 | 3.5 | 2.5 |
|  |  | | **Before Propensity Matching** | | | | **After Propensity Matching** | | | |
|  |  | | **Opioid** | | **NSAID** | | **Opioid** | | **NSAID** | |
|  | Benzodiazepines | | 2176 | 4.2 | 81 | 5.6 | 226 | 5.4 | 77 | 5.4 |
|  | Muscle relaxants | | 1203 | 2.3 | 20 | 1.4 | 64 | 1.5 | 20 | 1.4 |
|  | Stimulants | | 30 | 0.1 | - ^a^ | - ^a^ | - ^a^ | - ^a^ | - ^a^ | - ^a^ |
|  | Zolpidem | | 392 | 0.8 | 19 | 1.3 | 50 | 1.2 | 17 | 1.2 |
|  | Antidepressants | | 2101 | 4.0 | 121 | 8.3 | 300 | 7.2 | 107 | 7.5 |
|  | Antipsychotics | | 508 | 1.0 | 46 | 3.2 | 87 | 2.1 | 39 | 2.7 |
|  | Diuretics | | 5246 | 10.1 | 164 | 11.3 | 494 | 11.9 | 160 | 11.2 |
|  | ACE-I/ARBs | | 4069 | 7.8 | 195 | 13.4 | 518 | 12.5 | 189 | 13.2 |
|  | Acid-suppressive medications | | 4791 | 9.2 | 211 | 14.5 | 589 | 14.2 | 204 | 14.3 |
| Prior high-dose long-term opioid use | | | 33 | 0.1 | - ^a^ | - ^a^ | - ^a^ | - ^a^ | - ^a^ | - ^a^ |
| Abbreviations: ACE-I/ARB = angiotensin converting enzyme inhibitor/angiotensin receptor blocker; d = days; ESRD = end-stage renal disease; HIV/AIDS = human immunodeficiency virus/acquired immunodeficiency virus; NSAID = non-steroidal anti-inflammatory drug; s.d. = standard deviation | | | | | | | | | | |
| ^a^ Cell suppressed owing to small cell size, in accordance with CMS policy | | | | | | | | | | |
