# Supplementary material for: Relative risks of adverse events among older adults receiving opioids versus NSAIDs after hospital discharge: A nationwide cohort study
Source: PLoS Med. 2021 Sep 27;18(9):e1003804. doi: 10.1371/journal.pmed.1003804 (PMC8504723; doi:10.1371/journal.pmed.1003804)
Supplement: S5 Table — Characteristics of study population, before and after propensity matching. (DOCX) [file pmed.1003804.s005.docx]

| **S5 Table. Subgroup analysis in medical hospitalizations. Characteristics of study population, before and after propensity matching (see Appendix Figure 1 for standardized mean differences; all <0.1 after the match).** | | | | | | | | | |
| --- | --- | --- | --- | --- | --- | --- | --- | --- | --- |
|  | | **Before Propensity Matching** | | | | **After Propensity Matching** | | | |
|  | | **Opioid** | | **NSAID** | | **Opioid** | | **NSAID** | |
| **Characteristic – n % unless otherwise noted** | | n=36,789 | | n=3,438 | | n=9,475 | | n=3,398 | |
| Age in years – mean, s.d. | | 75.8 | 7.5 | 76.4 | 7.9 | 76.4 | 7.8 | 76.4 | 7.9 |
| Male | | 13924 | 37.9 | 1214 | 35.3 | 3342 | 35.3 | 1202 | 35.4 |
| Race | |  |  |  |  |  |  |  |  |
|  | Black | 4284 | 11.6 | 424 | 12.3 | 1212 | 12.8 | 418 | 12.3 |
|  | White | 30385 | 82.6 | 2634 | 76.6 | 7334 | 77.4 | 2610 | 76.8 |
|  | Other | 2120 | 5.8 | 380 | 11.1 | 929 | 9.8 | 370 | 10.9 |
| Original reason for entitlement | |  |  |  |  |  |  |  |  |
|  | Age | 26789 | 72.8 | 2564 | 74.6 | 7060 | 74.5 | 2536 | 74.6 |
|  | Disability/ESRD | 10000 | 27.2 | 874 | 25.4 | 2415 | 25.5 | 862 | 25.4 |
| Medicaid dual eligible | | 11985 | 32.6 | 1447 | 42.1 | 3808 | 40.2 | 1415 | 41.6 |
| Prior diagnoses | |  |  |  |  |  |  |  |  |
|  | Congestive heart failure | 13273 | 36.1 | 986 | 28.7 | 2840 | 30.0 | 980 | 28.8 |
|  | Cardiac arrhythmias | 16440 | 44.7 | 1282 | 37.3 | 3663 | 38.7 | 1272 | 37.4 |
|  | Valvular disease | 7391 | 20.1 | 560 | 16.3 | 1627 | 17.2 | 555 | 16.3 |
|  | Pulmonary circulation disorders | 5152 | 14.0 | 305 | 8.9 | 905 | 9.6 | 303 | 8.9 |
|  | Peripheral vascular disorders | 10616 | 28.9 | 834 | 24.3 | 2328 | 24.6 | 827 | 24.3 |
|  | Hypertension, uncomplicated | 32326 | 87.9 | 3026 | 88.0 | 8344 | 88.1 | 2989 | 88.0 |
|  | Hypertension, complicated | 13018 | 35.4 | 827 | 24.1 | 2355 | 24.9 | 827 | 24.3 |
|  | Paralysis | 1024 | 2.8 | 97 | 2.8 | 262 | 2.8 | 96 | 2.8 |
|  | Other neurological disorders | 6251 | 17.0 | 632 | 18.4 | 1676 | 17.7 | 619 | 18.2 |
|  | Chronic pulmonary disease | 17387 | 47.3 | 1518 | 44.2 | 4217 | 44.5 | 1499 | 44.1 |
|  | Diabetes, uncomplicated | 15262 | 41.5 | 1385 | 40.3 | 3780 | 39.9 | 1372 | 40.4 |
|  | Diabetes, complicated | 11065 | 30.1 | 904 | 26.3 | 2478 | 26.2 | 899 | 26.5 |
|  | Hypothyroidism | 10134 | 27.5 | 903 | 26.3 | 2524 | 26.6 | 896 | 26.4 |
|  | Renal failure | 12744 | 34.6 | 719 | 20.9 | 2101 | 22.2 | 719 | 21.2 |
|  |  | **Before Propensity Matching** | | | | **After Propensity Matching** | | | |
|  |  | **Opioid** | | **NSAID** | | **Opioid** | | **NSAID** | |
|  | Liver disease | 3373 | 9.2 | 209 | 6.1 | 593 | 6.3 | 209 | 6.2 |
|  | AIDS/HIV | 96 | 0.3 | -^a^ | -^a^ | 19 | 0.2 | -^a^ | -^a^ |
|  | Lymphoma | 1098 | 3.0 | 64 | 1.9 | 182 | 1.9 | 64 | 1.9 |
|  | Metastatic cancer | 3327 | 9.0 | 119 | 3.5 | 365 | 3.9 | 119 | 3.5 |
|  | Solid tumor without metastasis | 7553 | 20.5 | 432 | 12.6 | 1242 | 13.1 | 431 | 12.7 |
|  | Rheumatoid arthritis/collagen vascular diseases | 4420 | 12.0 | 416 | 12.1 | 1205 | 12.7 | 412 | 12.1 |
|  | Coagulopathy | 4646 | 12.6 | 293 | 8.5 | 868 | 9.2 | 293 | 8.6 |
|  | Obesity | 8819 | 24.0 | 743 | 21.6 | 2081 | 22.0 | 735 | 21.6 |
|  | Weight loss | 5527 | 15.0 | 318 | 9.2 | 925 | 9.8 | 316 | 9.3 |
|  | Fluid and electrolyte disorders | 19587 | 53.2 | 1602 | 46.6 | 4526 | 47.8 | 1587 | 46.7 |
|  | Blood loss anemia | 1644 | 4.5 | 89 | 2.6 | 280 | 3.0 | 89 | 2.6 |
|  | Deficiency anemia | 7297 | 19.8 | 502 | 14.6 | 1476 | 15.6 | 501 | 14.7 |
|  | Alcohol abuse | 1737 | 4.7 | 178 | 5.2 | 451 | 4.8 | 175 | 5.2 |
|  | Psychoses | 954 | 2.6 | 200 | 5.8 | 407 | 4.3 | 179 | 5.3 |
|  | Depression | 11699 | 31.8 | 1078 | 31.4 | 2923 | 30.8 | 1064 | 31.3 |
|  | Osteoporosis | 4158 | 11.3 | 358 | 10.4 | 1006 | 10.6 | 355 | 10.4 |
|  | Migraine and chronic headache | 1303 | 3.5 | 135 | 3.9 | 371 | 3.9 | 132 | 3.9 |
|  | Bipolar disorder | 1140 | 3.1 | 135 | 3.9 | 337 | 3.6 | 127 | 3.7 |
|  | Anxiety disorder | 10375 | 28.2 | 899 | 26.1 | 2466 | 26.0 | 885 | 26.0 |
|  | Opioid use disorder | 5544 | 15.1 | 319 | 9.3 | 932 | 9.8 | 317 | 9.3 |
|  | Drug use disorder | 1311 | 3.6 | 125 | 3.6 | 325 | 3.4 | 124 | 3.6 |
|  | Dementia | 4009 | 10.9 | 525 | 15.3 | 1324 | 14.0 | 513 | 15.1 |
|  | Falls/fractures | 19 | 0.1 | -^a^ | -^a^ | 14 | 0.1 | -^a^ | -^a^ |
|  | Delirium | 4216 | 11.5 | 380 | 11.1 | 1005 | 10.6 | 369 | 10.9 |
|  | Nausea/vomiting | 10621 | 28.9 | 757 | 22.0 | 2220 | 23.4 | 751 | 22.1 |
|  | Constipation/ileus/impaction/obstruction | 11263 | 30.6 | 835 | 24.3 | 2324 | 24.5 | 829 | 24.4 |
|  | Acute renal failure | 10855 | 29.5 | 702 | 20.4 | 2091 | 22.1 | 699 | 20.6 |
|  | Upper gastrointestinal inflammation/ulcer/bleeding | 5557 | 15.1 | 333 | 9.7 | 964 | 10.2 | 332 | 9.8 |
| Frailty/function | |  |  |  |  |  |  |  |  |
|  |  | **Before Propensity Matching** | | | | **After Propensity Matching** | | | |
|  |  | **Opioid** | | **NSAID** | | **Opioid** | | **NSAID** | |
|  | Frailty Index – mean, s.d. | 0.3 | 0.1 | 0.3 | 0.1 | 0.3 | 0.1 | 0.3 | 0.1 |
|  | Home healthcare claims | 11724 | 31.9 | 1017 | 29.6 | 2811 | 29.7 | 1004 | 29.5 |
|  | Skilled nursing facility claims | 4078 | 11.1 | 280 | 8.1 | 796 | 8.4 | 277 | 8.2 |
|  | Mobility impairment | 1740 | 4.7 | 142 | 4.1 | 396 | 4.2 | 141 | 4.1 |
| Hospitalization characteristics | |  |  |  |  |  |  |  |  |
|  | Length of stay – mean, s.d. | 4.3 | 3.9 | 3.8 | 4.7 | 3.8 | 3.4 | 3.8 | 4.7 |
|  | Any time in intensive care | 8302 | 22.6 | 789 | 22.9 | 2200 | 23.2 | 782 | 23.0 |
| Primary discharge diagnosis | |  |  |  |  |  |  |  |  |
|  | Infectious and parasitic diseases | 2722 | 7.4 | 257 | 7.5 | 738 | 7.8 | 255 | 7.5 |
|  | Neoplasms | 1811 | 4.9 | 48 | 1.4 | 154 | 1.6 | 48 | 1.4 |
|  | Endocrine; nutritional; and metabolic diseases and immunity disorders | 1400 | 3.8 | 177 | 5.1 | 481 | 5.1 | 175 | 5.2 |
|  | Diseases of the blood and blood-forming organs | 548 | 1.5 | 47 | 1.4 | 137 | 1.4 | 47 | 1.4 |
|  | Mental illness | 582 | 1.6 | 201 | 5.8 | 397 | 4.2 | 183 | 5.4 |
|  | Diseases of the nervous system and sense organs | 1121 | 3.0 | 135 | 3.9 | 357 | 3.8 | 135 | 4.0 |
|  | Diseases of the circulatory system | 6667 | 18.1 | 752 | 21.9 | 2096 | 22.1 | 747 | 22.0 |
|  | Diseases of the respiratory system | 5016 | 13.6 | 605 | 17.6 | 1622 | 17.1 | 595 | 17.5 |
|  | Diseases of the digestive system | 5052 | 13.7 | 378 | 11.0 | 1096 | 11.6 | 378 | 11.1 |
|  | Diseases of the genitourinary system | 3137 | 8.5 | 272 | 7.9 | 783 | 8.3 | 269 | 7.9 |
|  | Diseases of the skin and subcutaneous tissue | 1371 | 3.7 | 128 | 3.7 | 369 | 3.9 | 128 | 3.8 |
|  | Diseases of the musculoskeletal system and connective tissue | 1636 | 4.4 | 136 | 4.0 | 366 | 3.9 | 136 | 4.0 |
|  | Injury and poisoning | 4427 | 12.0 | 199 | 5.8 | 565 | 6.0 | 199 | 5.9 |
|  | Symptoms; signs; and ill-defined conditions and factors influencing health status | 1137 | 3.1 | 88 | 2.6 | 261 | 2.8 | 88 | 2.6 |
|  | Residual codes; unclassified; all E codes | 144 | 0.4 | 15 | 0.4 | 48 | 0.5 | 15 | 0.4 |
| Primary discharge procedure | |  |  |  |  |  |  |  |  |
|  | Operations on the nervous system | 374 | 1.0 | 38 | 1.1 | 100 | 1.1 | 38 | 1.1 |
|  | Operations on the endocrine system | 17 | 0.0 | -^a^ | -^a^ | -^a^ | -^a^ | -^a^ | -^a^ |
|  | Operations on the eye | -^a^ | -^a^ | -^a^ | -^a^ | -^a^ | -^a^ | -^a^ | -^a^ |
|  |  | **Before Propensity Matching** | | | | **After Propensity Matching** | | | |
|  |  | **Opioid** | | **NSAID** | | **Opioid** | | **NSAID** | |
|  | Operations on the ear | 26 | 0.1 | -^a^ | -^a^ | -^a^ | -^a^ | -^a^ | -^a^ |
|  | Operations on the nose, mouth, and pharynx | 118 | 0.3 | -^a^ | -^a^ | -^a^ | -^a^ | -^a^ | -^a^ |
|  | Operations on the respiratory system | 1188 | 3.2 | 65 | 1.9 | 171 | 1.8 | 65 | 1.9 |
|  | Operations on the cardiovascular system | 2553 | 6.9 | 208 | 6.1 | 620 | 6.5 | 208 | 6.1 |
|  | Operations on the hemic and lymphatic system | 117 | 0.3 | -^a^ | -^a^ | 11 | 0.1 | -^a^ | -^a^ |
|  | Operations on the digestive system | 3390 | 9.2 | 185 | 5.4 | 550 | 5.8 | 185 | 5.4 |
|  | Operations on the urinary system | 768 | 2.1 | 26 | 0.8 | 73 | 0.8 | 26 | 0.8 |
|  | Operations on the male genital organs | 28 | 0.1 | -^a^ | -^a^ | -^a^ | -^a^ | -^a^ | -^a^ |
|  | Operations on the female genital organs | -^a^ | -^a^ | -^a^ | -^a^ | -^a^ | -^a^ | -^a^ | -^a^ |
|  | Operations on the musculoskeletal system | 512 | 1.4 | 49 | 1.4 | 137 | 1.4 | 49 | 1.4 |
|  | Operations on the integumentary system | 668 | 1.8 | 36 | 1.0 | 95 | 1.0 | 36 | 1.1 |
|  | Miscellaneous diagnostic and therapeutic procedures | 4534 | 12.3 | 444 | 12.9 | 1254 | 13.2 | 436 | 12.8 |
| Number of prior hospitalizations – mean, s.d. | | 1.4 | 2.1 | 1.0 | 1.7 | 1.0 | 1.8 | 1.0 | 1.7 |
| Medication use in prior 90d | |  |  |  |  |  |  |  |  |
|  | Number of claims – mean, s.d. | 16.7 | 11.7 | 18.3 | 13.3 | 17.7 | 12.9 | 18.2 | 13.2 |
|  | Benzodiazepines | 9631 | 26.2 | 822 | 23.9 | 2334 | 24.6 | 809 | 23.8 |
|  | Muscle relaxants | 3170 | 8.6 | 304 | 8.8 | 836 | 8.8 | 302 | 8.9 |
|  | Stimulants | 247 | 0.7 | 16 | 0.5 | 58 | 0.6 | 15 | 0.4 |
|  | Zolpidem | 2065 | 5.6 | 182 | 5.3 | 520 | 5.5 | 178 | 5.2 |
|  | Antidepressants | 13680 | 37.2 | 1334 | 38.8 | 3570 | 37.7 | 1310 | 38.6 |
|  | Antipsychotics | 2585 | 7.0 | 339 | 9.9 | 802 | 8.5 | 318 | 9.4 |
|  | Diuretics | 15830 | 43.0 | 1441 | 41.9 | 4003 | 42.2 | 1425 | 41.9 |
|  | ACE-I/ARBs | 15239 | 41.4 | 1599 | 46.5 | 4254 | 44.9 | 1579 | 46.5 |
|  | Acid-suppressive medications | 15545 | 42.3 | 1545 | 44.9 | 4146 | 43.8 | 1519 | 44.7 |
| Medication use within 7d of discharge | |  |  |  |  |  |  |  |  |
|  | Number of claims – mean, s.d. | 4.2 | 2.8 | 5.1 | 3.5 | 4.8 | 3.3 | 5.0 | 3.4 |
|  | Benzodiazepines | 4170 | 11.3 | 318 | 9.2 | 865 | 9.1 | 313 | 9.2 |
|  | Muscle relaxants | 1094 | 3.0 | 110 | 3.2 | 301 | 3.2 | 110 | 3.2 |
|  | Stimulants | 90 | 0.2 | -^a^ | -^a^ | 11 | 0.1 | -^a^ | -^a^ |
|  |  | **Before Propensity Matching** | | | | **After Propensity Matching** | | | |
|  |  | **Opioid** | | **NSAID** | | **Opioid** | | **NSAID** | |
|  | Zolpidem | 624 | 1.7 | 53 | 1.5 | 150 | 1.6 | 52 | 1.5 |
|  | Antidepressants | 4141 | 11.3 | 677 | 19.7 | 1649 | 17.4 | 648 | 19.1 |
|  | Antipsychotics | 1077 | 2.9 | 221 | 6.4 | 454 | 4.8 | 199 | 5.9 |
|  | Diuretics | 5487 | 14.9 | 680 | 19.8 | 1792 | 18.9 | 667 | 19.6 |
|  | ACE-I/ARBs | 4165 | 11.3 | 697 | 20.3 | 1760 | 18.6 | 678 | 20.0 |
|  | Acid-suppressive medications | 5585 | 15.2 | 818 | 23.8 | 2001 | 21.1 | 788 | 23.2 |
| Prior high-dose long-term opioid use | | 2546 | 6.9 | 91 | 2.6 | 308 | 3.3 | 90 | 2.6 |
| Abbreviations: ACE-I/ARB = angiotensin converting enzyme inhibitor/angiotensin receptor blocker; d = days; ESRD = end-stage renal disease; HIV/AIDS = human immunodeficiency virus/acquired immunodeficiency virus; NSAID = non-steroidal anti-inflammatory drug; s.d. = standard deviation | | | | | | | | | |
| ^a^ Cell suppressed owing to small cell size, in accordance with CMS policy | | | | | | | | | |
