# Supplementary material for: Relative risks of adverse events among older adults receiving opioids versus NSAIDs after hospital discharge: A nationwide cohort study
Source: PLoS Med. 2021 Sep 27;18(9):e1003804. doi: 10.1371/journal.pmed.1003804 (PMC8504723; doi:10.1371/journal.pmed.1003804)
Supplement: S6 Table — Characteristics of study population, before and after propensity matching. (DOCX) [file pmed.1003804.s006.docx]

| **S6 Table. Subgroup analysis in surgical hospitalizations. Characteristics of study population, before and after propensity matching (see Appendix Figure 1 for standardized mean differences; all <0.1 after the match).** | | | | | | | | | |
| --- | --- | --- | --- | --- | --- | --- | --- | --- | --- |
|  | | **Before Propensity Matching** | | | | **After Propensity Matching** | | | |
|  | | **Opioid** | | **NSAID** | | **Opioid** | | **NSAID** | |
| **Characteristic – n % unless otherwise noted** | | n=74,272 | | n=1,275 | | n=3,777 | | n=1,270 | |
| Age in years – mean, s.d. | | 73.5 | 5.7 | 74.5 | 6.3 | 74.5 | 6.3 | 74.4 | 6.3 |
| Male | | 33490 | 45.1 | 489 | 38.4 | 1473 | 39.0 | 489 | 38.5 |
| Race | |  |  |  |  |  |  |  |  |
|  | Black | 4199 | 5.7 | 70 | 5.5 | 196 | 5.2 | 69 | 5.4 |
|  | White | 66391 | 89.4 | 1108 | 86.9 | 3298 | 87.3 | 1105 | 87.0 |
|  | Other | 3682 | 5.0 | 97 | 7.6 | 283 | 7.5 | 96 | 7.6 |
| Original reason for entitlement | |  |  |  |  |  |  |  |  |
|  | Age | 64258 | 86.5 | 1085 | 85.1 | 3211 | 85.0 | 1081 | 85.1 |
|  | Disability/ESRD | 10014 | 13.5 | 190 | 14.9 | 566 | 15.0 | 189 | 14.9 |
| Medicaid dual eligible | | 9201 | 12.4 | 247 | 19.4 | 709 | 18.8 | 244 | 19.2 |
| Prior diagnoses | |  |  |  |  |  |  |  |  |
|  | Congestive heart failure | 10125 | 13.6 | 179 | 14.0 | 559 | 14.8 | 179 | 14.1 |
|  | Cardiac arrhythmias | 20821 | 28.0 | 375 | 29.4 | 1129 | 29.9 | 373 | 29.4 |
|  | Valvular disease | 10355 | 13.9 | 192 | 15.1 | 575 | 15.2 | 191 | 15.0 |
|  | Pulmonary circulation disorders | 3572 | 4.8 | 69 | 5.4 | 197 | 5.2 | 69 | 5.4 |
|  | Peripheral vascular disorders | 13468 | 18.1 | 214 | 16.8 | 623 | 16.5 | 213 | 16.8 |
|  | Hypertension, uncomplicated | 60253 | 81.1 | 1020 | 80.0 | 3039 | 80.5 | 1015 | 79.9 |
|  | Hypertension, complicated | 12393 | 16.7 | 175 | 13.7 | 514 | 13.6 | 172 | 13.5 |
|  | Paralysis | 661 | 0.9 | 16 | 1.3 | 46 | 1.2 | 16 | 1.3 |
|  | Other neurological disorders | 4075 | 5.5 | 76 | 6.0 | 227 | 6.0 | 74 | 5.8 |
|  | Chronic pulmonary disease | 19067 | 25.7 | 308 | 24.2 | 944 | 25.0 | 308 | 24.3 |
|  | Diabetes, uncomplicated | 21598 | 29.1 | 352 | 27.6 | 1023 | 27.1 | 351 | 27.6 |
|  | Diabetes, complicated | 12224 | 16.5 | 181 | 14.2 | 517 | 13.7 | 180 | 14.2 |
|  | Hypothyroidism | 16941 | 22.8 | 304 | 23.8 | 945 | 25.0 | 303 | 23.9 |
|  | Renal failure | 12240 | 16.5 | 162 | 12.7 | 476 | 12.6 | 162 | 12.8 |
|  |  | **Before Propensity Matching** | | | | **After Propensity Matching** | | | |
|  |  | **Opioid** | | **NSAID** | | **Opioid** | | **NSAID** | |
|  | Liver disease | 3530 | 4.8 | 57 | 4.5 | 168 | 4.4 | 56 | 4.4 |
|  | AIDS/HIV | 96 | 0.1 | -^a^ | -^a^ | -^a^ | -^a^ | -^a^ | -^a^ |
|  | Lymphoma | 1057 | 1.4 | -^a^ | -^a^ | 24 | 0.6 | -^a^ | -^a^ |
|  | Metastatic cancer | 3466 | 4.7 | 76 | 6.0 | 239 | 6.3 | 76 | 6.0 |
|  | Solid tumor without metastasis | 15418 | 20.8 | 282 | 22.1 | 881 | 23.3 | 282 | 22.2 |
|  | Rheumatoid arthritis/collagen vascular diseases | 6329 | 8.5 | 135 | 10.6 | 402 | 10.6 | 134 | 10.6 |
|  | Coagulopathy | 5282 | 7.1 | 64 | 5.0 | 216 | 5.7 | 64 | 5.0 |
|  | Obesity | 17183 | 23.1 | 271 | 21.3 | 810 | 21.4 | 271 | 21.3 |
|  | Weight loss | 3557 | 4.8 | 56 | 4.4 | 160 | 4.2 | 56 | 4.4 |
|  | Fluid and electrolyte disorders | 16202 | 21.8 | 266 | 20.9 | 779 | 20.6 | 266 | 20.9 |
|  | Blood loss anemia | 1632 | 2.2 | 28 | 2.2 | 85 | 2.3 | 27 | 2.1 |
|  | Deficiency anemia | 6474 | 8.7 | 88 | 6.9 | 288 | 7.6 | 88 | 6.9 |
|  | Alcohol abuse | 1709 | 2.3 | 11 | 0.9 | 40 | 1.1 | 11 | 0.9 |
|  | Psychoses | 444 | 0.6 | 11 | 0.9 | 30 | 0.8 | 11 | 0.9 |
|  | Depression | 13570 | 18.3 | 234 | 18.4 | 732 | 19.4 | 234 | 18.4 |
|  | Osteoporosis | 5388 | 7.3 | 104 | 8.2 | 321 | 8.5 | 103 | 8.1 |
|  | Migraine and chronic headache | 1669 | 2.2 | 37 | 2.9 | 119 | 3.2 | 36 | 2.8 |
|  | Bipolar disorder | 849 | 1.1 | 16 | 1.3 | 50 | 1.3 | 16 | 1.3 |
|  | Anxiety disorder | 11391 | 15.3 | 199 | 15.6 | 633 | 16.8 | 198 | 15.6 |
|  | Opioid use disorder | 4757 | 6.4 | 95 | 7.5 | 295 | 7.8 | 95 | 7.5 |
|  | Drug use disorder | 770 | 1.0 | -^a^ | -^a^ | 38 | 1.0 | -^a^ | -^a^ |
|  | Dementia | 1817 | 2.4 | 56 | 4.4 | 177 | 4.7 | 54 | 4.3 |
|  | Falls/fractures | 37 | 0.0 | -^a^ | -^a^ | -^a^ | -^a^ | -^a^ | -^a^ |
|  | Delirium | 1804 | 2.4 | 32 | 2.5 | 101 | 2.7 | 31 | 2.4 |
|  | Nausea/vomiting | 13481 | 18.2 | 228 | 17.9 | 673 | 17.8 | 226 | 17.8 |
|  | Constipation/ileus/impaction/obstruction | 14608 | 19.7 | 227 | 17.8 | 687 | 18.2 | 227 | 17.9 |
|  | Acute renal failure | 6661 | 9.0 | 114 | 8.9 | 337 | 8.9 | 114 | 9.0 |
|  | Upper gastrointestinal inflammation/ulcer/bleeding | 4098 | 5.5 | 60 | 4.7 | 177 | 4.7 | 60 | 4.7 |
| Frailty/function | |  |  |  |  |  |  |  |  |
|  |  | **Before Propensity Matching** | | | | **After Propensity Matching** | | | |
|  |  | **Opioid** | | **NSAID** | | **Opioid** | | **NSAID** | |
|  | Frailty Index – mean, s.d. | 0.2 | 0.1 | 0.2 | 0.1 | 0.2 | 0.1 | 0.2 | 0.1 |
|  | Home healthcare claims | 9124 | 12.3 | 222 | 17.4 | 715 | 18.9 | 218 | 17.2 |
|  | Skilled nursing facility claims | 2413 | 3.2 | 35 | 2.7 | 102 | 2.7 | 35 | 2.8 |
|  | Mobility impairment | 1105 | 1.5 | 23 | 1.8 | 80 | 2.1 | 25 | 2.0 |
| Hospitalization characteristics | |  |  |  |  |  |  |  |  |
|  | Length of stay – mean, s.d. | 3.5 | 3.5 | 3.4 | 3.2 | 3.4 | 3.1 | 3.4 | 3.3 |
|  | Any time in intensive care | 14327 | 19.3 | 256 | 20.1 | 764 | 20.2 | 254 | 20.0 |
| Primary discharge diagnosis | |  |  |  |  |  |  |  |  |
|  | Infectious and parasitic diseases | 701 | 0.9 | 18 | 1.4 | 51 | 1.4 | 18 | 1.4 |
|  | Neoplasms | 9492 | 12.8 | 161 | 12.6 | 502 | 13.3 | 160 | 12.6 |
|  | Endocrine; nutritional; and metabolic diseases and immunity disorders | 915 | 1.2 | 14 | 1.1 | 44 | 1.2 | 14 | 1.1 |
|  | Diseases of the blood and blood-forming organs | 57 | 0.1 | -^a^ | -^a^ | -^a^ | -^a^ | -^a^ | -^a^ |
|  | Mental illness | 12 | 0.0 | -^a^ | -^a^ | -^a^ | -^a^ | -^a^ | -^a^ |
|  | Diseases of the nervous system and sense organs | 473 | 0.6 | 15 | 1.2 | 36 | 1.0 | 15 | 1.2 |
|  | Diseases of the circulatory system | 9937 | 13.4 | 211 | 16.5 | 610 | 16.2 | 208 | 16.4 |
|  | Diseases of the respiratory system | 710 | 1.0 | 20 | 1.6 | 71 | 1.9 | 20 | 1.6 |
|  | Diseases of the digestive system | 7656 | 10.3 | 120 | 9.4 | 369 | 9.8 | 120 | 9.4 |
|  | Diseases of the genitourinary system | 1885 | 2.5 | 78 | 6.1 | 220 | 5.8 | 77 | 6.1 |
|  | Diseases of the skin and subcutaneous tissue | 429 | 0.6 | -^a^ | -^a^ | 34 | 0.9 | -^a^ | -^a^ |
|  | Diseases of the musculoskeletal system and connective tissue | 34607 | 46.6 | 485 | 38.0 | 1413 | 37.4 | 485 | 38.2 |
|  | Injury and poisoning | 6923 | 9.3 | 137 | 10.7 | 399 | 10.6 | 137 | 10.8 |
|  | Symptoms; signs; and ill-defined conditions and factors influencing health status | 293 | 0.4 | -^a^ | -^a^ | 15 | 0.4 | -^a^ | -^a^ |
|  | Residual codes; unclassified; all E codes | 17 | 0.0 | -^a^ | -^a^ | -^a^ | -^a^ | -^a^ | -^a^ |
| Primary discharge procedure | |  |  |  |  |  |  |  |  |
|  | Operations on the nervous system | 2851 | 3.8 | 27 | 2.1 | 95 | 2.5 | 27 | 2.1 |
|  | Operations on the endocrine system | 313 | 0.4 | -^a^ | -^a^ | 14 | 0.4 | -^a^ | -^a^ |
|  | Operations on the eye | 29 | 0.0 | -^a^ | -^a^ | -^a^ | -^a^ | -^a^ | -^a^ |
|  |  | **Before Propensity Matching** | | | | **After Propensity Matching** | | | |
|  |  | **Opioid** | | **NSAID** | | **Opioid** | | **NSAID** | |
|  | Operations on the ear | 59 | 0.1 | -^a^ | -^a^ | -^a^ | -^a^ | -^a^ | -^a^ |
|  | Operations on the nose, mouth, and pharynx | 231 | 0.3 | -^a^ | -^a^ | 23 | 0.6 | -^a^ | -^a^ |
|  | Operations on the respiratory system | 2513 | 3.4 | 45 | 3.5 | 132 | 3.5 | 45 | 3.5 |
|  | Operations on the cardiovascular system | 10862 | 14.6 | 238 | 18.7 | 701 | 18.6 | 235 | 18.5 |
|  | Operations on the hemic and lymphatic system | 569 | 0.8 | -^a^ | -^a^ | 13 | 0.3 | -^a^ | -^a^ |
|  | Operations on the digestive system | 11494 | 15.5 | 180 | 14.1 | 540 | 14.3 | 180 | 14.2 |
|  | Operations on the urinary system | 2312 | 3.1 | 27 | 2.1 | 85 | 2.3 | 27 | 2.1 |
|  | Operations on the male genital organs | 1483 | 2.0 | 17 | 1.3 | 63 | 1.7 | 17 | 1.3 |
|  | Operations on the female genital organs | 1255 | 1.7 | 99 | 7.8 | 301 | 8.0 | 98 | 7.7 |
|  | Operations on the musculoskeletal system | 38380 | 51.7 | 568 | 44.5 | 1642 | 43.5 | 567 | 44.6 |
|  | Operations on the integumentary system | 1504 | 2.0 | 41 | 3.2 | 128 | 3.4 | 41 | 3.2 |
|  | Miscellaneous diagnostic and therapeutic procedures | 417 | 0.6 | -^a^ | -^a^ | 29 | 0.8 | -^a^ | -^a^ |
| Number of prior hospitalizations – mean, s.d. | | 0.4 | 1.0 | 0.5 | 0.9 | 0.5 | 1.0 | 0.5 | 0.9 |
| Medication use in prior 90d | |  |  |  |  |  |  |  |  |
|  | Number of claims – mean, s.d. | 10.0 | 7.8 | 11.6 | 9.8 | 11.6 | 9.6 | 11.6 | 9.7 |
|  | Benzodiazepines | 10747 | 14.5 | 193 | 15.1 | 583 | 15.4 | 193 | 15.2 |
|  | Muscle relaxants | 3472 | 4.7 | 62 | 4.9 | 197 | 5.2 | 62 | 4.9 |
|  | Stimulants | 368 | 0.5 | -^a^ | -^a^ | 35 | 0.9 | -^a^ | -^a^ |
|  | Zolpidem | 3076 | 4.1 | 55 | 4.3 | 176 | 4.7 | 55 | 4.3 |
|  | Antidepressants | 18621 | 25.1 | 328 | 25.7 | 974 | 25.8 | 325 | 25.6 |
|  | Antipsychotics | 1546 | 2.1 | 30 | 2.4 | 86 | 2.3 | 29 | 2.3 |
|  | Diuretics | 25793 | 34.7 | 444 | 34.8 | 1283 | 34.0 | 441 | 34.7 |
|  | ACE-I/ARBs | 32026 | 43.1 | 556 | 43.6 | 1626 | 43.1 | 553 | 43.5 |
|  | Acid-suppressive medications | 21414 | 28.8 | 423 | 33.2 | 1224 | 32.4 | 420 | 33.1 |
| Medication use within 7d of discharge | |  |  |  |  |  |  |  |  |
|  | Number of claims – mean, s.d. | 2.8 | 1.9 | 3.2 | 2.8 | 3.2 | 2.5 | 3.2 | 2.8 |
|  | Benzodiazepines | 3545 | 4.8 | 66 | 5.2 | 215 | 5.7 | 66 | 5.2 |
|  | Muscle relaxants | 2493 | 3.4 | 28 | 2.2 | 77 | 2.0 | 28 | 2.2 |
|  | Stimulants | 50 | 0.1 | -^a^ | -^a^ | -^a^ | -^a^ | -^a^ | -^a^ |
|  |  | **Before Propensity Matching** | | | | **After Propensity Matching** | | | |
|  |  | **Opioid** | | **NSAID** | | **Opioid** | | **NSAID** | |
|  | Zolpidem | 693 | 0.9 | -^a^ | -^a^ | 30 | 0.8 | -^a^ | -^a^ |
|  | Antidepressants | 3113 | 4.2 | 99 | 7.8 | 279 | 7.4 | 97 | 7.6 |
|  | Antipsychotics | 561 | 0.8 | 14 | 1.1 | 39 | 1.0 | 13 | 1.0 |
|  | Diuretics | 6229 | 8.4 | 132 | 10.4 | 376 | 10.0 | 130 | 10.2 |
|  | ACE-I/ARBs | 5137 | 6.9 | 169 | 13.3 | 476 | 12.6 | 165 | 13.0 |
|  | Acid-suppressive medications | 6143 | 8.3 | 173 | 13.6 | 469 | 12.4 | 170 | 13.4 |
| Prior high-dose long-term opioid use | | 1071 | 1.4 | 22 | 1.7 | 75 | 2.0 | 22 | 1.7 |
| Abbreviations: ACE-I/ARB = angiotensin converting enzyme inhibitor/angiotensin receptor blocker; d = days; ESRD = end-stage renal disease; HIV/AIDS = human immunodeficiency virus/acquired immunodeficiency virus; NSAID = non-steroidal anti-inflammatory drug; s.d. = standard deviation | | | | | | | | | |
| ^a^ Cell suppressed owing to small cell size, in accordance with CMS policy | | | | | | | | | |
