# Supplementary material for: Relative risks of adverse events among older adults receiving opioids versus NSAIDs after hospital discharge: A nationwide cohort study
Source: PLoS Med. 2021 Sep 27;18(9):e1003804. doi: 10.1371/journal.pmed.1003804 (PMC8504723; doi:10.1371/journal.pmed.1003804)
Supplement: S7 Table — Characteristics of study population, before and after propensity matching. (DOCX) [file pmed.1003804.s007.docx]

| **S7 Table. Subgroup analysis in beneficiaries without cancer. Characteristics of study population, before and after propensity matching (see Appendix Figure 1 for standardized mean differences; all <0.1 after the match).** | | | | | | | | | | |
| --- | --- | --- | --- | --- | --- | --- | --- | --- | --- | --- |
|  | | | **Before Propensity Matching** | | | | **After Propensity Matching** | | | |
|  | | | **Opioid** | | **NSAID** | | **Opioid** | | **NSAID** | |
| **Characteristic – n % unless otherwise noted** | | | n=86,194 | | n=3,936 | | n=11,049 | | n=3,898 | |
| Age in years – mean, s.d. | | | 74.3 | 6.5 | 75.9 | 7.6 | 75.9 | 7.6 | 75.9 | 7.6 |
| Male | | | 34759 | 40.3 | 1350 | 34.3 | 3811 | 34.5 | 1340 | 34.4 |
| Race | | |  |  |  |  |  |  |  |  |
|  | Black | | 6325 | 7.3 | 408 | 10.4 | 1165 | 10.5 | 405 | 10.4 |
|  | White | | 75393 | 87.5 | 3119 | 79.2 | 8844 | 80.0 | 3088 | 79.2 |
|  | Other | | 4476 | 5.2 | 409 | 10.4 | 1040 | 9.4 | 405 | 10.4 |
| Original reason for entitlement | | |  |  |  |  |  |  |  |  |
|  | Age | | 70021 | 81.2 | 3010 | 76.5 | 8343 | 75.5 | 2985 | 76.6 |
|  | Disability/ESRD | | 16173 | 18.8 | 926 | 23.5 | 2706 | 24.5 | 913 | 23.4 |
| Medicaid dual eligible | | | 16806 | 19.5 | 1479 | 37.6 | 3959 | 35.8 | 1450 | 37.2 |
| Prior diagnoses | | |  |  |  |  |  |  |  |  |
|  | Congestive heart failure | | 18717 | 21.7 | 1005 | 25.5 | 2949 | 26.7 | 998 | 25.6 |
|  | Cardiac arrhythmias | | 28696 | 33.3 | 1391 | 35.3 | 4028 | 36.5 | 1375 | 35.3 |
|  | Valvular disease | | 14059 | 16.3 | 617 | 15.7 | 1789 | 16.2 | 613 | 15.7 |
|  | Pulmonary circulation disorders | | 6662 | 7.7 | 308 | 7.8 | 963 | 8.7 | 306 | 7.9 |
|  | Peripheral vascular disorders | | 18836 | 21.9 | 876 | 22.3 | 2578 | 23.3 | 872 | 22.4 |
|  | Hypertension, uncomplicated | | 72266 | 83.8 | 3390 | 86.1 | 9480 | 85.8 | 3355 | 86.1 |
|  | Hypertension, complicated | | 19837 | 23.0 | 863 | 21.9 | 2579 | 23.3 | 860 | 22.1 |
|  | Paralysis | | 1361 | 1.6 | 94 | 2.4 | 265 | 2.4 | 92 | 2.4 |
|  | Other neurological disorders | | 8088 | 9.4 | 632 | 16.1 | 1713 | 15.5 | 617 | 15.8 |
|  | Chronic pulmonary disease | | 27662 | 32.1 | 1517 | 38.5 | 4340 | 39.3 | 1497 | 38.4 |
|  | Diabetes, uncomplicated | | 28698 | 33.3 | 1475 | 37.5 | 4144 | 37.5 | 1460 | 37.5 |
|  | Diabetes, complicated | | 18411 | 21.4 | 936 | 23.8 | 2663 | 24.1 | 927 | 23.8 |
|  | Hypothyroidism | | 21442 | 24.9 | 1017 | 25.8 | 2909 | 26.3 | 1006 | 25.8 |
|  | Renal failure | | 19252 | 22.3 | 747 | 19.0 | 2255 | 20.4 | 746 | 19.1 |
|  |  | | **Before Propensity Matching** | | | | **After Propensity Matching** | | | |
|  |  | | **Opioid** | | **NSAID** | | **Opioid** | | **NSAID** | |
|  | Liver disease | | 4529 | 5.3 | 202 | 5.1 | 580 | 5.2 | 202 | 5.2 |
|  | AIDS/HIV | | 149 | 0.2 | -^a^ | -^a^ | 28 | 0.3 | -^a^ | -^a^ |
|  | Rheumatoid arthritis/collagen vascular diseases | | 8920 | 10.3 | 475 | 12.1 | 1389 | 12.6 | 474 | 12.2 |
|  | Coagulopathy | | 6958 | 8.1 | 270 | 6.9 | 841 | 7.6 | 270 | 6.9 |
|  | Obesity | | 21167 | 24.6 | 886 | 22.5 | 2494 | 22.6 | 879 | 22.6 |
|  | Weight loss | | 5524 | 6.4 | 286 | 7.3 | 855 | 7.7 | 286 | 7.3 |
|  | Fluid and electrolyte disorders | | 26420 | 30.7 | 1548 | 39.3 | 4449 | 40.3 | 1532 | 39.3 |
|  | Blood loss anemia | | 2199 | 2.6 | 96 | 2.4 | 290 | 2.6 | 96 | 2.5 |
|  | Deficiency anemia | | 9967 | 11.6 | 463 | 11.8 | 1371 | 12.4 | 461 | 11.8 |
|  | Alcohol abuse | | 2610 | 3.0 | 161 | 4.1 | 421 | 3.8 | 159 | 4.1 |
|  | Psychoses | | 1142 | 1.3 | 196 | 5.0 | 417 | 3.8 | 175 | 4.5 |
|  | Depression | | 20164 | 23.4 | 1102 | 28.0 | 3102 | 28.1 | 1090 | 28.0 |
|  | Osteoporosis | | 7656 | 8.9 | 399 | 10.1 | 1166 | 10.6 | 396 | 10.2 |
|  | Migraine and chronic headache | | 2466 | 2.9 | 150 | 3.8 | 406 | 3.7 | 147 | 3.8 |
|  | Bipolar disorder | | 1635 | 1.9 | 131 | 3.3 | 341 | 3.1 | 125 | 3.2 |
|  | Anxiety disorder | | 17157 | 19.9 | 922 | 23.4 | 2595 | 23.5 | 911 | 23.4 |
|  | Opioid use disorder | | 8465 | 9.8 | 365 | 9.3 | 1112 | 10.1 | 363 | 9.3 |
|  | Drug use disorder | | 1634 | 1.9 | 118 | 3.0 | 314 | 2.8 | 116 | 3.0 |
|  | Dementia | | 4833 | 5.6 | 528 | 13.4 | 1386 | 12.5 | 515 | 13.2 |
|  | Falls/fractures | | 45 | 0.1 | -^a^ | -^a^ | -^a^ | -^a^ | -^a^ | -^a^ |
|  | Delirium | | 4700 | 5.5 | 362 | 9.2 | 1020 | 9.2 | 354 | 9.1 |
|  | Nausea/vomiting | | 16817 | 19.5 | 759 | 19.3 | 2169 | 19.6 | 750 | 19.2 |
|  | Constipation/ileus/impaction/obstruction | | 18356 | 21.3 | 850 | 21.6 | 2477 | 22.4 | 842 | 21.6 |
|  | Acute renal failure | | 13033 | 15.1 | 694 | 17.6 | 2099 | 19.0 | 688 | 17.7 |
|  | Upper gastrointestinal inflammation/ulcer/bleeding | | 7169 | 8.3 | 324 | 8.2 | 999 | 9.0 | 323 | 8.3 |
| Frailty/function | | |  |  |  |  |  |  |  |  |
|  | Frailty Index – mean, s.d. | | 0.2 | 0.1 | 0.3 | 0.1 | 0.3 | 0.1 | 0.3 | 0.1 |
|  | Home healthcare claims | | 16030 | 18.6 | 1050 | 26.7 | 2965 | 26.8 | 1034 | 26.5 |
|  | Skilled nursing facility claims | | 5092 | 5.9 | 256 | 6.5 | 796 | 7.2 | 255 | 6.5 |
|  |  | | **Before Propensity Matching** | | | | **After Propensity Matching** | | | |
|  |  | | **Opioid** | | **NSAID** | | **Opioid** | | **NSAID** | |
|  | Mobility impairment | | 2324 | 2.7 | 143 | 3.6 | 420 | 3.8 | 141 | 3.6 |
| Hospitalization characteristics | | |  |  |  |  |  |  |  |  |
|  | Length of stay – mean, s.d. | | 3.6 | 3.5 | 3.7 | 4.4 | 3.7 | 3.6 | 3.6 | 4.4 |
|  | Any time in intensive care | | 16878 | 19.6 | 894 | 22.7 | 2534 | 22.9 | 887 | 22.8 |
|  | Diagnosis-related group | |  |  |  |  |  |  |  |  |
|  |  | Medical | 28246 | 32.8 | 2954 | 75.1 | 8228 | 74.5 | 2916 | 74.8 |
|  |  | Surgical | 57948 | 67.2 | 982 | 25.0 | 2821 | 25.5 | 982 | 25.2 |
| Primary discharge diagnosis | | |  |  |  |  |  |  |  |  |
|  | Infectious and parasitic diseases | | 2574 | 3.0 | 222 | 5.6 | 643 | 5.8 | 220 | 5.6 |
|  | Neoplasms | | 1246 | 1.4 | 34 | 0.9 | 107 | 1.0 | 34 | 0.9 |
|  | Endocrine; nutritional; and metabolic diseases and immunity disorders | | 1873 | 2.2 | 159 | 4.0 | 432 | 3.9 | 158 | 4.1 |
|  | Diseases of the blood and blood-forming organs | | 386 | 0.4 | 36 | 0.9 | 100 | 0.9 | 36 | 0.9 |
|  | Mental illness | | 526 | 0.6 | 183 | 4.6 | 373 | 3.4 | 168 | 4.3 |
|  | Diseases of the nervous system and sense organs | | 1203 | 1.4 | 134 | 3.4 | 362 | 3.3 | 133 | 3.4 |
|  | Diseases of the circulatory system | | 14323 | 16.6 | 854 | 21.7 | 2377 | 21.5 | 846 | 21.7 |
|  | Diseases of the respiratory system | | 4526 | 5.3 | 548 | 13.9 | 1493 | 13.5 | 540 | 13.9 |
|  | Diseases of the digestive system | | 10336 | 12.0 | 423 | 10.7 | 1247 | 11.3 | 422 | 10.8 |
|  | Diseases of the genitourinary system | | 3964 | 4.6 | 294 | 7.5 | 899 | 8.1 | 292 | 7.5 |
|  | Diseases of the skin and subcutaneous tissue | | 1536 | 1.8 | 122 | 3.1 | 333 | 3.0 | 122 | 3.1 |
|  | Diseases of the musculoskeletal system and connective tissue | | 32629 | 37.9 | 550 | 14.0 | 1546 | 14.0 | 550 | 14.1 |
|  | Injury and poisoning | | 9655 | 11.2 | 286 | 7.3 | 889 | 8.0 | 286 | 7.3 |
|  | Symptoms; signs; and ill-defined conditions and factors influencing health status | | 1128 | 1.3 | 80 | 2.0 | 213 | 1.9 | 80 | 2.1 |
|  | Residual codes; unclassified; all E codes | | 125 | 0.1 | 11 | 0.3 | 30 | 0.3 | 11 | 0.3 |
| Primary discharge procedure | | |  |  |  |  |  |  |  |  |
|  | Operations on the nervous system | | 2616 | 3.0 | 54 | 1.4 | 162 | 1.5 | 54 | 1.4 |
|  | Operations on the endocrine system | | 191 | 0.2 | -^a^ | -^a^ | 16 | 0.1 | -^a^ | -^a^ |
|  | Operations on the eye | | 22 | 0.0 | -^a^ | -^a^ | -^a^ | -^a^ | -^a^ | -^a^ |
|  |  | | **Before Propensity Matching** | | | | **After Propensity Matching** | | | |
|  |  | | **Opioid** | | **NSAID** | | **Opioid** | | **NSAID** | |
|  | Operations on the ear | | 61 | 0.1 | -^a^ | -^a^ | -^a^ | -^a^ | -^a^ | -^a^ |
|  | Operations on the nose, mouth, and pharynx | | 145 | 0.2 | -^a^ | -^a^ | 20 | 0.2 | -^a^ | -^a^ |
|  | Operations on the respiratory system | | 1264 | 1.5 | 54 | 1.4 | 169 | 1.5 | 54 | 1.4 |
|  | Operations on the cardiovascular system | | 11600 | 13.5 | 391 | 9.9 | 1108 | 10.0 | 390 | 10.0 |
|  | Operations on the hemic and lymphatic system | | 146 | 0.2 | -^a^ | -^a^ | 15 | 0.1 | -^a^ | -^a^ |
|  | Operations on the digestive system | | 9842 | 11.4 | 267 | 6.8 | 799 | 7.2 | 266 | 6.8 |
|  | Operations on the urinary system | | 1407 | 1.6 | 34 | 0.9 | 116 | 1.0 | 34 | 0.9 |
|  | Operations on the male genital organs | | 372 | 0.4 | -^a^ | -^a^ | 12 | 0.1 | -^a^ | -^a^ |
|  | Operations on the female genital organs | | 576 | 0.7 | 65 | 1.7 | 221 | 2.0 | 65 | 1.7 |
|  | Operations on the musculoskeletal system | | 34895 | 40.5 | 539 | 13.7 | 1527 | 13.8 | 539 | 13.8 |
|  | Operations on the integumentary system | | 1399 | 1.6 | 52 | 1.3 | 160 | 1.4 | 52 | 1.3 |
|  | Miscellaneous diagnostic and therapeutic procedures | | 3601 | 4.2 | 372 | 9.5 | 1008 | 9.1 | 367 | 9.4 |
| Number of prior hospitalizations – mean, s.d. | | | 0.7 | 1.5 | 0.8 | 1.5 | 0.8 | 1.5 | 0.8 | 1.5 |
| Medication use in prior 90d | | |  |  |  |  |  |  |  |  |
|  | Number of claims – mean, s.d. | | 12.3 | 10.0 | 16.8 | 12.8 | 16.2 | 12.8 | 16.6 | 12.5 |
|  | Benzodiazepines | | 15516 | 18.0 | 841 | 21.4 | 2364 | 21.4 | 831 | 21.3 |
|  | Muscle relaxants | | 5585 | 6.5 | 315 | 8.0 | 890 | 8.1 | 312 | 8.0 |
|  | Stimulants | | 482 | 0.6 | 17 | 0.4 | 58 | 0.5 | 17 | 0.4 |
|  | Zolpidem | | 3994 | 4.6 | 192 | 4.9 | 539 | 4.9 | 187 | 4.8 |
|  | Antidepressants | | 25981 | 30.1 | 1438 | 36.5 | 3926 | 35.5 | 1407 | 36.1 |
|  | Antipsychotics | | 2676 | 3.1 | 309 | 7.9 | 701 | 6.3 | 288 | 7.4 |
|  | Diuretics | | 33260 | 38.6 | 1609 | 40.9 | 4439 | 40.2 | 1590 | 40.8 |
|  | ACE-I/ARBs | | 37482 | 43.5 | 1823 | 46.3 | 5014 | 45.4 | 1803 | 46.3 |
|  | Acid-suppressive medications | | 28658 | 33.2 | 1681 | 42.7 | 4591 | 41.6 | 1653 | 42.4 |
| Medication use within 7d of discharge | | |  |  |  |  |  |  |  |  |
|  | Number of claims – mean, s.d. | | 3.3 | 2.4 | 4.7 | 3.5 | 4.5 | 3.3 | 4.6 | 3.4 |
|  | Benzodiazepines | | 6081 | 7.1 | 332 | 8.4 | 982 | 8.9 | 325 | 8.3 |
|  | Muscle relaxants | | 3108 | 3.6 | 121 | 3.1 | 354 | 3.2 | 121 | 3.1 |
|  | Stimulants | | 104 | 0.1 | -^a^ | -^a^ | 16 | 0.1 | -^a^ | -^a^ |
|  |  | | **Before Propensity Matching** | | | | **After Propensity Matching** | | | |
|  |  | | **Opioid** | | **NSAID** | | **Opioid** | | **NSAID** | |
|  | Zolpidem | | 1034 | 1.2 | 53 | 1.3 | 158 | 1.4 | 51 | 1.3 |
|  | Antidepressants | | 5842 | 6.8 | 671 | 17.0 | 1670 | 15.1 | 638 | 16.4 |
|  | Antipsychotics | | 1124 | 1.3 | 216 | 5.5 | 431 | 3.9 | 195 | 5.0 |
|  | Diuretics | | 9409 | 10.9 | 704 | 17.9 | 1799 | 16.3 | 687 | 17.6 |
|  | ACE-I/ARBs | | 7538 | 8.7 | 758 | 19.3 | 1943 | 17.6 | 735 | 18.9 |
|  | Acid-suppressive medications | | 9069 | 10.5 | 854 | 21.7 | 2186 | 19.8 | 824 | 21.1 |
| Prior high-dose long-term opioid use | | | 2753 | 3.2 | 93 | 2.4 | 337 | 3.1 | 93 | 2.4 |
| Abbreviations: ACE-I/ARB = angiotensin converting enzyme inhibitor/angiotensin receptor blocker; d = days; ESRD = end-stage renal disease; HIV/AIDS = human immunodeficiency virus/acquired immunodeficiency virus; NSAID = non-steroidal anti-inflammatory drug; s.d. = standard deviation | | | | | | | | | | |
| ^a^ Cell suppressed owing to small cell size, in accordance with CMS policy | | | | | | | | | | |
