# Supplementary material for: Relative risks of adverse events among older adults receiving opioids versus NSAIDs after hospital discharge: A nationwide cohort study
Source: PLoS Med. 2021 Sep 27;18(9):e1003804. doi: 10.1371/journal.pmed.1003804 (PMC8504723; doi:10.1371/journal.pmed.1003804)
Supplement: S8 Table — Characteristics of study population, before and after propensity matching. NSAID, nonsteroidal anti-inflammatory drug. (DOCX) [file pmed.1003804.s008.docx]

| **S8 Table. Subgroup analysis using mutually exclusive exposure groups (i.e., after excluding beneficiaries with claims for both opioids and NSAIDs within 7 days of discharge, n=6,355). Characteristics of study population, before and after propensity matching (see Appendix Figure 1 for standardized mean differences; all <0.1 after the match).** | | | | | | | | | | |
| --- | --- | --- | --- | --- | --- | --- | --- | --- | --- | --- |
|  | | | **Before Propensity Matching** | | | | **After Propensity Matching** | | | |
|  | | | **Opioid** | | **NSAID** | | **Opioid** | | **NSAID** | |
| **Characteristic – n % unless otherwise noted** | | | n=104,706 | | n=4,713 | | n=13,237 | | n=4,647 | |
| Age in years – mean, s.d. | | | 74.3 | 6.5 | 75.9 | 7.5 | 75.9 | 7.5 | 75.9 | 7.5 |
| Male | | | 45076 | 43.1 | 1703 | 36.1 | 4785 | 36.2 | 1679 | 36.1 |
| Race | | |  |  |  |  |  |  |  |  |
|  | Black | | 8134 | 7.8 | 494 | 10.5 | 1403 | 10.6 | 488 | 10.5 |
|  | White | | 91116 | 87.0 | 3742 | 79.4 | 10623 | 80.3 | 3698 | 79.6 |
|  | Other | | 5456 | 5.2 | 477 | 10.1 | 1211 | 9.2 | 461 | 9.9 |
| Original reason for entitlement | | |  |  |  |  |  |  |  |  |
|  | Age | | 85520 | 81.7 | 3649 | 77.4 | 10349 | 78.2 | 3603 | 77.5 |
|  | Disability/ESRD | | 19186 | 18.3 | 1064 | 22.58 | 2888 | 21.8 | 1044 | 22.5 |
| Medicaid dual eligible | | | 20205 | 19.3 | 1694 | 35.94 | 4456 | 33.7 | 1642 | 35.3 |
| Prior diagnoses | | |  |  |  |  |  |  |  |  |
|  | Congestive heart failure | | 22797 | 21.8 | 1165 | 24.7 | 3382 | 25.5 | 1153 | 24.8 |
|  | Cardiac arrhythmias | | 35907 | 34.3 | 1657 | 35.2 | 4813 | 36.4 | 1640 | 35.3 |
|  | Valvular disease | | 17112 | 16.3 | 752 | 16.0 | 2228 | 16.8 | 745 | 16.0 |
|  | Pulmonary circulation disorders | | 8527 | 8.1 | 374 | 7.9 | 1070 | 8.1 | 372 | 8.0 |
|  | Peripheral vascular disorders | | 23316 | 22.3 | 1048 | 22.2 | 3001 | 22.7 | 1038 | 22.3 |
|  | Hypertension, uncomplicated | | 87624 | 83.7 | 4046 | 85.8 | 11396 | 86.1 | 3987 | 85.8 |
|  | Hypertension, complicated | | 24737 | 23.6 | 1002 | 21.3 | 3022 | 22.8 | 994 | 21.4 |
|  | Paralysis | | 1628 | 1.6 | 113 | 2.4 | 322 | 2.4 | 111 | 2.4 |
|  | Other neurological disorders | | 9922 | 9.5 | 708 | 15.0 | 1893 | 14.3 | 691 | 14.9 |
|  | Chronic pulmonary disease | | 34958 | 33.4 | 1826 | 38.7 | 5178 | 39.1 | 1796 | 38.6 |
|  | Diabetes, uncomplicated | | 35299 | 33.7 | 1737 | 36.9 | 4819 | 36.4 | 1711 | 36.8 |
|  | Diabetes, complicated | | 22489 | 21.5 | 1085 | 23.0 | 3025 | 22.9 | 1072 | 23.1 |
|  | Hypothyroidism | | 25542 | 24.4 | 1207 | 25.6 | 3340 | 25.2 | 1192 | 25.7 |
|  |  | | **Before Propensity Matching** | | | | **After Propensity Matching** | | | |
|  |  | | **Opioid** | | **NSAID** | | **Opioid** | | **NSAID** | |
|  | Renal failure | | 24351 | 23.3 | 881 | 18.7 | 2709 | 20.5 | 877 | 18.9 |
|  | Liver disease | | 6664 | 6.4 | 266 | 5.6 | 794 | 6.0 | 263 | 5.7 |
|  | AIDS/HIV | | 189 | 0.2 | -^a^ | -^a^ | 25 | 0.2 | -^a^ | -^a^ |
|  | Lymphoma | | 2098 | 2.0 | 73 | 1.5 | 253 | 1.9 | 73 | 1.6 |
|  | Metastatic cancer | | 6532 | 6.2 | 195 | 4.1 | 636 | 4.8 | 195 | 4.2 |
|  | Solid tumor without metastasis | | 21914 | 20.9 | 714 | 15.1 | 2130 | 16.1 | 707 | 15.2 |
|  | Rheumatoid arthritis/collagen vascular diseases | | 10181 | 9.7 | 551 | 11.7 | 1564 | 11.8 | 545 | 11.7 |
|  | Coagulopathy | | 9604 | 9.2 | 357 | 7.6 | 1059 | 8.0 | 357 | 7.7 |
|  | Obesity | | 24488 | 23.4 | 1014 | 21.5 | 2889 | 21.8 | 1003 | 21.6 |
|  | Weight loss | | 8870 | 8.5 | 374 | 7.9 | 1126 | 8.5 | 374 | 8.0 |
|  | Fluid and electrolyte disorders | | 34596 | 33.0 | 1868 | 39.6 | 5394 | 40.7 | 1846 | 39.7 |
|  | Blood loss anemia | | 3183 | 3.0 | 117 | 2.5 | 358 | 2.7 | 117 | 2.5 |
|  | Deficiency anemia | | 13345 | 12.7 | 590 | 12.5 | 1742 | 13.2 | 585 | 12.6 |
|  | Alcohol abuse | | 3303 | 3.2 | 189 | 4.0 | 524 | 4.0 | 186 | 4.0 |
|  | Psychoses | | 1342 | 1.3 | 211 | 4.5 | 443 | 3.3 | 183 | 3.9 |
|  | Depression | | 23924 | 22.8 | 1312 | 27.8 | 3665 | 27.7 | 1289 | 27.7 |
|  | Osteoporosis | | 9038 | 8.6 | 462 | 9.8 | 1312 | 9.9 | 456 | 9.8 |
|  | Migraine and chronic headache | | 2794 | 2.7 | 172 | 3.6 | 494 | 3.7 | 167 | 3.6 |
|  | Bipolar disorder | | 1885 | 1.8 | 151 | 3.2 | 396 | 3.0 | 140 | 3.0 |
|  | Anxiety disorder | | 20633 | 19.7 | 1098 | 23.3 | 3092 | 23.4 | 1072 | 23.1 |
|  | Opioid use disorder | | 9854 | 9.4 | 414 | 8.8 | 1237 | 9.3 | 412 | 8.9 |
|  | Drug use disorder | | 1993 | 1.9 | 135 | 2.9 | 370 | 2.8 | 134 | 2.9 |
|  | Dementia | | 5609 | 5.4 | 581 | 12.3 | 1506 | 11.4 | 559 | 12.0 |
|  | Falls/fractures | | 51 | 0.0 | -^a^ | -^a^ | 13 | 0.1 | -^a^ | -^a^ |
|  | Delirium | | 5847 | 5.6 | 412 | 8.7 | 1145 | 8.6 | 401 | 8.6 |
|  | Nausea/vomiting | | 22835 | 21.8 | 985 | 20.9 | 2826 | 21.3 | 968 | 20.8 |
|  | Constipation/ileus/impaction/obstruction | | 24650 | 23.5 | 1062 | 22.5 | 3083 | 23.3 | 1042 | 22.4 |
|  | Acute renal failure | | 17149 | 16.4 | 816 | 17.3 | 2379 | 18.0 | 809 | 17.4 |
|  | Upper gastrointestinal inflammation/ulcer/bleeding | | 9375 | 9.0 | 393 | 8.3 | 1157 | 8.7 | 390 | 8.4 |
|  | | | **Before Propensity Matching** | | | | **After Propensity Matching** | | | |
|  | | | **Opioid** | | **NSAID** | | **Opioid** | | **NSAID** | |
| Frailty/function | | |  |  |  |  |  |  |  |  |
|  | Frailty Index – mean, s.d. | | 0.2 | 0.1 | 0.2 | 0.1 | 0.2 | 0.1 | 0.2 | 0.1 |
|  | Home healthcare claims | | 20007 | 19.1 | 1239 | 26.3 | 3428 | 25.9 | 1214 | 26.1 |
|  | Skilled nursing facility claims | | 6301 | 6.0 | 315 | 6.7 | 947 | 7.2 | 309 | 6.6 |
|  | Mobility impairment | | 2732 | 2.6 | 167 | 3.5 | 494 | 3.7 | 165 | 3.6 |
| Hospitalization characteristics | | |  |  |  |  |  |  |  |  |
|  | Length of stay – mean, s.d. | | 3.8 | 3.7 | 3.7 | 4.3 | 3.7 | 3.6 | 3.7 | 4.3 |
|  | Any time in intensive care | | 22060 | 21.1 | 1045 | 22.2 | 2962 | 22.4 | 1031 | 22.2 |
|  | Diagnosis-related group | |  |  |  |  |  |  |  |  |
|  |  | Medical | 35577 | 34.0 | 3438 | 73.0 | 9503 | 71.8 | 3372 | 72.6 |
|  |  | Surgical | 69129 | 66.0 | 1275 | 27.1 | 3734 | 28.2 | 1275 | 27.4 |
| Primary discharge diagnosis | | |  |  |  |  |  |  |  |  |
|  | Infectious and parasitic diseases | | 3332 | 3.2 | 275 | 5.8 | 770 | 5.8 | 271 | 5.8 |
|  | Neoplasms | | 10766 | 10.3 | 209 | 4.4 | 653 | 4.9 | 209 | 4.5 |
|  | Endocrine; nutritional; and metabolic diseases and immunity disorders | | 2265 | 2.2 | 191 | 4.1 | 528 | 4.0 | 187 | 4.0 |
|  | Diseases of the blood and blood-forming organs | | 598 | 0.6 | 48 | 1.0 | 146 | 1.1 | 47 | 1.0 |
|  | Mental illness | | 549 | 0.5 | 201 | 4.3 | 376 | 2.8 | 177 | 3.8 |
|  | Diseases of the nervous system and sense organs | | 1524 | 1.5 | 150 | 3.2 | 432 | 3.3 | 148 | 3.2 |
|  | Diseases of the circulatory system | | 16303 | 15.6 | 963 | 20.4 | 2693 | 20.3 | 952 | 20.5 |
|  | Diseases of the respiratory system | | 5532 | 5.3 | 625 | 13.3 | 1666 | 12.6 | 614 | 13.2 |
|  | Diseases of the digestive system | | 12379 | 11.8 | 498 | 10.6 | 1458 | 11.0 | 495 | 10.7 |
|  | Diseases of the genitourinary system | | 4773 | 4.6 | 350 | 7.4 | 1048 | 7.9 | 347 | 7.5 |
|  | Diseases of the skin and subcutaneous tissue | | 1740 | 1.7 | 138 | 2.9 | 389 | 2.9 | 136 | 2.9 |
|  | Diseases of the musculoskeletal system and connective tissue | | 32475 | 31.0 | 621 | 13.2 | 1669 | 12.6 | 621 | 13.4 |
|  | Injury and poisoning | | 10763 | 10.3 | 336 | 7.1 | 1077 | 8.1 | 336 | 7.2 |
|  | Symptoms; signs; and ill-defined conditions and factors influencing health status | | 1370 | 1.3 | 93 | 2.0 | 280 | 2.1 | 92 | 2.0 |
|  | Residual codes; unclassified; all E codes | | 154 | 0.1 | 15 | 0.3 | 47 | 0.4 | 15 | 0.3 |
|  | | | **Before Propensity Matching** | | | | **After Propensity Matching** | | | |
|  | | | **Opioid** | | **NSAID** | | **Opioid** | | **NSAID** | |
| Primary discharge procedure | | |  |  |  |  |  |  |  |  |
|  | Operations on the nervous system | | 3105 | 3.0 | 65 | 1.4 | 211 | 1.6 | 64 | 1.4 |
|  | Operations on the endocrine system | | 324 | 0.3 | -^a^ | -^a^ | 25 | 0.2 | -^a^ | -^a^ |
|  | Operations on the eye | | 36 | 0.0 | -^a^ | -^a^ | 12 | 0.1 | -^a^ | -^a^ |
|  | Operations on the ear | | 82 | 0.1 | -^a^ | -^a^ | -^a^ | -^a^ | -^a^ | -^a^ |
|  | Operations on the nose, mouth, and pharynx | | 338 | 0.3 | -^a^ | -^a^ | 31 | 0.2 | -^a^ | -^a^ |
|  | Operations on the respiratory system | | 3540 | 3.4 | 110 | 2.3 | 328 | 2.5 | 110 | 2.4 |
|  | Operations on the cardiovascular system | | 13228 | 12.6 | 446 | 9.5 | 1294 | 9.8 | 445 | 9.6 |
|  | Operations on the hemic and lymphatic system | | 661 | 0.6 | -^a^ | -^a^ | 48 | 0.4 | -^a^ | -^a^ |
|  | Operations on the digestive system | | 14510 | 13.9 | 365 | 7.7 | 1089 | 8.2 | 363 | 7.8 |
|  | Operations on the urinary system | | 3030 | 2.9 | 53 | 1.1 | 177 | 1.3 | 53 | 1.1 |
|  | Operations on the male genital organs | | 1453 | 1.4 | 17 | 0.4 | 63 | 0.5 | 17 | 0.4 |
|  | Operations on the female genital organs | | 952 | 0.9 | 99 | 2.1 | 333 | 2.5 | 99 | 2.1 |
|  | Operations on the musculoskeletal system | | 34893 | 33.3 | 617 | 13.1 | 1664 | 12.6 | 617 | 13.3 |
|  | Operations on the integumentary system | | 2083 | 2.0 | 77 | 1.6 | 215 | 1.6 | 77 | 1.7 |
|  | Miscellaneous diagnostic and therapeutic procedures | | 4800 | 4.6 | 454 | 9.6 | 1263 | 9.5 | 443 | 9.5 |
| Number of prior hospitalizations – mean, s.d. | | | 0.8 | 1.5 | 0.8 | 1.5 | 0.9 | 1.6 | 0.8 | 1.5 |
| Medication use in prior 90d | | |  |  |  |  |  |  |  |  |
|  | Number of claims – mean, s.d. | | 12.3 | 9.8 | 16.5 | 12.8 | 16.0 | 12.4 | 16.3 | 12.5 |
|  | Benzodiazepines | | 19346 | 18.5 | 1015 | 21.5 | 2823 | 21.3 | 997 | 21.5 |
|  | Muscle relaxants | | 6273 | 6.0 | 366 | 7.8 | 998 | 7.5 | 361 | 7.8 |
|  | Stimulants | | 577 | 0.6 | 26 | 0.6 | 75 | 0.6 | 26 | 0.6 |
|  | Zolpidem | | 4864 | 4.6 | 237 | 5.0 | 684 | 5.2 | 233 | 5.0 |
|  | Antidepressants | | 30558 | 29.2 | 1662 | 35.3 | 4637 | 34.3 | 1662 | 35.1 |
|  | Antipsychotics | | 3935 | 3.8 | 369 | 7.8 | 952 | 7.0 | 364 | 7.7 |
|  | Diuretics | | 39467 | 37.7 | 1885 | 40.0 | 5478 | 40.6 | 1895 | 40.0 |
|  | ACE-I/ARBs | | 44614 | 42.6 | 2155 | 45.7 | 6023 | 44.6 | 2154 | 45.4 |
|  | Acid-suppressive medications | | 35107 | 33.5 | 1968 | 41.8 | 5478 | 40.6 | 1969 | 41.5 |
| Medication use within 7d of discharge | | |  |  |  |  |  |  |  |  |
|  |  | | **Before Propensity Matching** | | | | **After Propensity Matching** | | | |
|  |  | | **Opioid** | | **NSAID** | | **Opioid** | | **NSAID** | |
|  | Number of claims – mean, s.d. | | 3.2 | 2.3 | 4.6 | 3.4 | 4.3 | 3.1 | 4.5 | 3.3 |
|  | Benzodiazepines | | 7311 | 7.0 | 384 | 8.1 | 1100 | 8.3 | 369 | 7.9 |
|  | Muscle relaxants | | 3356 | 3.2 | 138 | 2.9 | 404 | 3.1 | 136 | 2.9 |
|  | Stimulants | | 134 | 0.1 | -^a^ | -^a^ | 17 | 0.1 | -^a^ | -^a^ |
|  | Zolpidem | | 1228 | 1.2 | 62 | 1.3 | 180 | 1.4 | 60 | 1.3 |
|  | Antidepressants | | 6779 | 6.5 | 776 | 16.5 | 1918 | 14.5 | 733 | 15.8 |
|  | Antipsychotics | | 1544 | 1.5 | 235 | 5.0 | 500 | 3.8 | 208 | 4.5 |
|  | Diuretics | | 11211 | 10.7 | 812 | 17.2 | 2089 | 15.8 | 788 | 17.0 |
|  | ACE-I/ARBs | | 8748 | 8.4 | 866 | 18.4 | 2195 | 16.6 | 829 | 17.8 |
|  | Acid-suppressive medications | | 10719 | 10.2 | 991 | 21.0 | 2515 | 19.0 | 936 | 20.1 |
| Prior high-dose long-term opioid use | | | 3473 | 3.3 | 113 | 2.4 | 386 | 2.9 | 113 | 2.4 |
| Abbreviations: ACE-I/ARB = angiotensin converting enzyme inhibitor/angiotensin receptor blocker; d = days; ESRD = end-stage renal disease; HIV/AIDS = human immunodeficiency virus/acquired immunodeficiency virus; NSAID = non-steroidal anti-inflammatory drug; s.d. = standard deviation | | | | | | | | | | |
| ^a^ Cell suppressed owing to small cell size, in accordance with CMS policy | | | | | | | | | | |
