# Supplementary material for: Relative risks of adverse events among older adults receiving opioids versus NSAIDs after hospital discharge: A nationwide cohort study
Source: PLoS Med. 2021 Sep 27;18(9):e1003804. doi: 10.1371/journal.pmed.1003804 (PMC8504723; doi:10.1371/journal.pmed.1003804)
Supplement: S9 Table — NSAID, nonsteroidal anti-inflammatory drug. (DOCX) [file pmed.1003804.s009.docx]

| **S9 Table. Relative risks and odds ratios of outcomes for opioids compared to NSAIDs in specified sensitivity analyses.** | | | | | | |
| --- | --- | --- | --- | --- | --- | --- |
|  | | **Opioid** | **NSAID** | **Unadjusted outcome models in the overall cohort** | **Adjusted outcome models in the overall cohort^a^** | **Propensity-matched analysis using a logit link^b^** |
|  | | **n=111,061** | **n=4,713** | **n=115,774** | **n=115,774** | **n=18,062** |
|  | | **n (%)** | **n (%)** | **Odds Ratio** | **Odds Ratio** | **Odds Ratio** |
| **Outcome** | |  |  | **[95% CI], p-value** | **[95% CI], p-value** | **[95% CI]** |
| Death | | 1635 (1.5) | 53 (1.1) | 1.3 [0.997-1.7], 0.05 | 1.5 [1.1-2.0], 0.004 | 1.7 [1.3-2.3], <0.001 |
| Healthcare utilization | | 18446 (16.6) | 818 (17.4) | 0.9 [0.9-1.02], 0.17 | 1.1 [1.04-1.2], 0.004 | 1.1 [1.02-1.2], 0.01 |
| Any potential adverse effect | | 27106 (24.4) | 1081 (22.9) | 1.1 [1.01-1.2], 0.02 | 1.3 [1.2-1.4], <0.001 | 1.2 [1.2-1.4], <0.001 |
|  | Fall/fracture | 4853 (4.4) | 159 (3.4) | 1.3 [1.1-1.5], 0.001 | 1.3 [1.1-1.6], 0.004 | 1.3 [1.1-1.6], 0.002 |
|  | Delirium | 1939 (1.8) | 102 (2.2) | 0.8 [0.7-0.98], 0.03 | 1.2 [0.98-1.5], 0.08 | 1.2 [0.98-1.5], 0.07 |
|  | Nausea/vomiting | 10220 (9.2) | 341 (7.2) | 1.3 [1.2-1.5], 0.001 | 1.4 [1.2-1.5], <0.001 | 1.3 [1.1-1.5], <0.001 |
|  | Slowed colonic motility | 7710 (6.9) | 297 (6.3) | 1.1 [0.98-1.3], 0.09 | 1.3 [1.2-1.5], <0.001 | 1.3 [1.1-1.5], <0.001 |
|  | Acute renal failure | 4620 (4.2) | 221 (4.7) | 0.9 [0.8-1.01], 0.08 | 1.0 [0.8-1.1], 0.71 | 1.0 [0.9-1.2], 0.74 |
|  | Gastritis/duodenitis | 3721 (3.4) | 208 (4.4) | 0.8 [0.7-0.9], 0.001 | 1.0 [0.8-1.1], 0.57 | 0.9 [0.8-1.1], 0.28 |
| Abbreviations: NSAID = non-steroidal anti-inflammatory drug | | | | | | |
| ^a^ GEE model of each outcome in the full (pre-match) opioid and NSAID cohorts, including all covariates, plus a variable representing opioid versus NSAID exposure, as independent variables. Because some of the models would not converge with a log link, a logit link was used instead, generating odds ratios rather than relative risks | | | | | | |
| ^b^ Because the outcome models in the overall cohort required use of a logit link to attain convergence, we present the results of the propensity-matched analysis using a logit link to facilitate direct comparisons with the outcome models in the overall cohort | | | | | | |
