# Supplementary material for: Relative risks of adverse events among older adults receiving opioids versus NSAIDs after hospital discharge: A nationwide cohort study
Source: PLoS Med. 2021 Sep 27;18(9):e1003804. doi: 10.1371/journal.pmed.1003804 (PMC8504723; doi:10.1371/journal.pmed.1003804)
Supplement: S1 Fig — SMD, standardized mean difference. (DOCX) [file pmed.1003804.s010.docx]

**S1 Fig. Standardized mean differences (SMDs), before and after each propensity match.** Figures show variables with SMD ≥ 0.1 before the match; variables with SMD < 0.1 are not depicted. Abbreviations: ACE-I/ARB = angiotensin converting enzyme inhibitor/angiotensin receptor blocker; d = days; DRG = diagnosis-related group; NSAID = non-steroidal anti-inflammatory drug.

a)

b)

c)

d)

e)

f)
