# Supplementary material for: Relative risks of adverse events among older adults receiving opioids versus NSAIDs after hospital discharge: A nationwide cohort study
Source: PLoS Med. 2021 Sep 27;18(9):e1003804. doi: 10.1371/journal.pmed.1003804 (PMC8504723; doi:10.1371/journal.pmed.1003804)

**S2 Fig. Propensity score distributions by group, before and after the match.**

Abbreviations: NSAID = non-steroidal anti-inflammatory drugs.

a) Overall


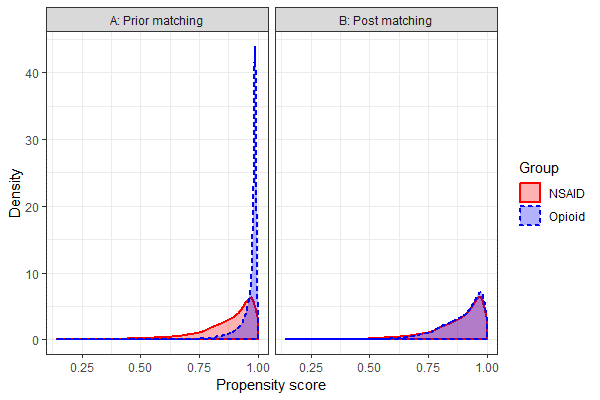


b) Mutually Exclusive Exposure Groups


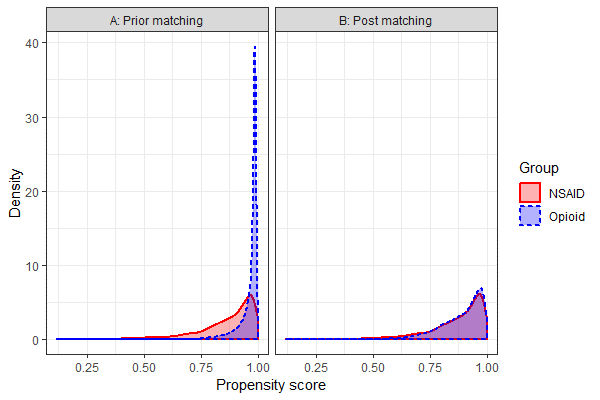


c) Beneficiaries without opioid or NSAID claims in the 90 days prior to hospitalization


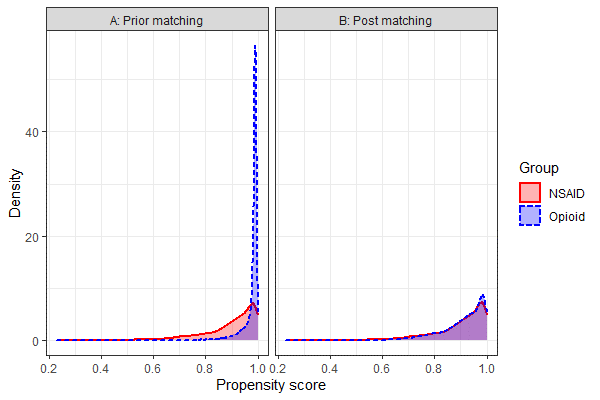


d) Medical Hospitalizations


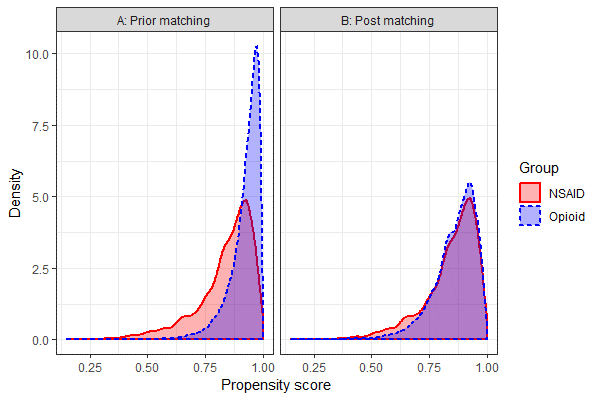


e) Surgical Hospitalizations


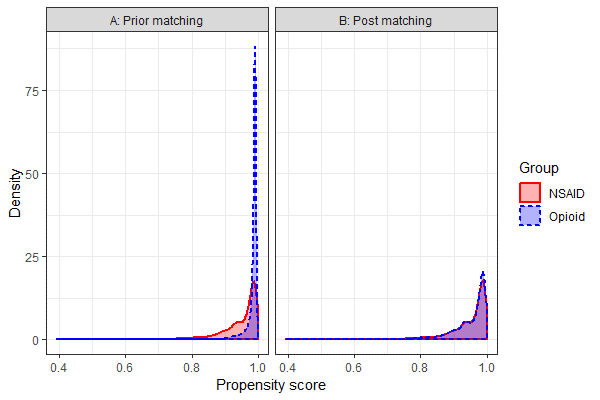


f) Beneficiaries without cancer


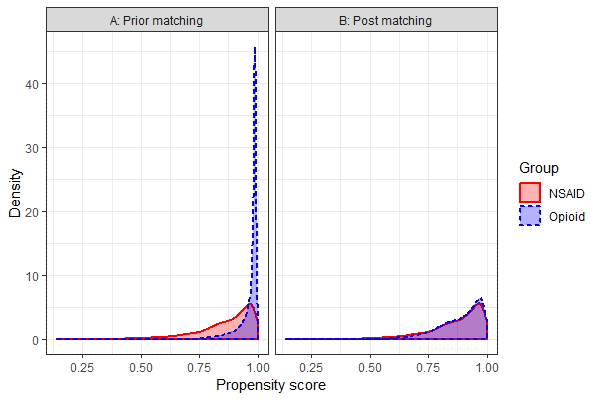

Supplement: S2 Fig — Density reflects the proportion of hospitalizations with a given propensity score. (DOCX) [file pmed.1003804.s011.docx]
