# Supplementary material for: Relative risks of adverse events among older adults receiving opioids versus NSAIDs after hospital discharge: A nationwide cohort study
Source: PLoS Med. 2021 Sep 27;18(9):e1003804. doi: 10.1371/journal.pmed.1003804 (PMC8504723; doi:10.1371/journal.pmed.1003804)
Supplement: S1 Protocol — (DOCX) [file pmed.1003804.s013.docx]

**Study Protocol**

**Aim 1: To define the incidence and predictors of adverse events within 30 days of hospital discharge among older adults with an opioid claim in the week after discharge using a large, national dataset.** Using Medicare claims, we will determine the national incidence and patient- and prescribing-related risk factors for post-discharge adverse events among older adults discharged on opioids, including falls, delirium, nausea, constipation, opioid overdose, emergency department visits, acute hospitalizations, and deaths.

Hypothesis 1A: The incidence of these adverse events within 30 days of discharge will be higher among patients with an opioid claim in the week after discharge compared to similar patients without such a claim.

Hypothesis 1B: We will identify several significant independent patient- and prescribing-related risk factors for post-discharge adverse events among patients with an opioid claim in the week after discharge.

Hypothesis 1C: Among patients with opioid use *prior to hospitalization,* distinct risk factors for post-discharge adverse events will exist, including dosage escalations and overlapping prescriptions.

**Overview:** For this aim, we will estimate the national incidence of adverse events within 30 days of hospital discharge among Medicare beneficiaries with an opioid claim within 7 days of discharge. Adverse events will include death, opioid overdose, measures of healthcare utilization (emergency department visits and acute hospitalizations) and clinical symptoms/conditions representing known risks of opioid therapy, (falls, delirium, nausea, constipation). We will compare rates of these adverse events between patients with an opioid claim within 7 days of discharge and a comparator group of propensity-matched patients, without an opioid claim within 7 days of discharge to determine the opioid attributable risk of these events. We will then use multivariable logistic regression to identify patient- and prescribing-related factors associated with such post-discharge adverse events among patients with an opioid claim within 7 days of discharge, with stratified analyses in medical/surgical patients and patients with/without opioid claims in the 90 days prior to admission.

**Data Sources:**  We will use the following Medicare files (Table 1) to identify our study sample, exposure, outcomes, and candidate patient- and prescribing-related factors described below. Two years of data will be purchased to create longitudinal histories for each beneficiary relative to their index hospitalization.

| **Table 1. Medicare files to be used in Aim 1** | |
| --- | --- |
| **Medicare File** | **Brief File Description** |
| Master Beneficiary Annual Summary File | Beneficiary demographic and Part A, B, C, D enrollment data, original entitlement reason, vital status, major comorbid conditions, summary health care utilization and costs |
| MedPAR File | Stays in acute hospitals and skilled nursing facilities (SNFs); includes dates, discharge diagnosis and procedure codes, ICU stay, and MS-DRG and revenue center codes |
| Part D Drug Event (PDE) File | Medication event claims; includes prescription details such as strength and route. Drug Characteristics Files and Prescriber Characteristics Files will be appended to PDE File |
| Carrier/Provider File | Claims for physician services; includes dates, diagnosis and procedure codes |
| Outpatient File | Claims from institutional outpatient providers and facilities; includes service dates, diagnosis and procedure codes, and revenue center codes |
| Hospice / Home Health Agency (HHA) Files | Claims for services by hospice providers and home health providers; includes service dates, number of visits, diagnosis, level of care (hospice), type of care (HHA) |
| Durable Medical Equipment (DME) File | Claims for DME; includes diagnosis and procedure codes |

**COHORT CONSTRUCTION**

We will use the 20% random sample of Medicare beneficiaries for the most recent year available to identify acute hospitalizations among Medicare beneficiaries age 66 and older who were discharged alive (at risk for adverse events). We will require ≥12 months of continuous fee-for-service (FFS) enrollment in Medicare Parts A, B and D prior to the admission date and 30 days following discharge, to allow ascertainment of comorbid conditions, pre- and post-hospitalization opioid exposure, and 30-day adverse events. We will exclude beneficiaries with use of hospice services in the prior year, or discharge to hospice since these patients are more likely to receive opioids and to experience mortality unrelated to opioid use. For beneficiaries meeting these criteria who had multiple hospitalizations during the study year, we will randomly select a single hospitalization as the index hospitalization (from which to evaluate the 30-day risk period for adverse events). This study population is our primary sample for analyses addressing Hypotheses 1A, 1B and 1C.

**MedPAR Data – 2016**

Using 2016 MedPAR, identify all Short-Stay Hospitalizations discharged alive (SSLSSNF= S and DSCHRGCD= A)

Sort by discharge date (DSCHRGDT) – When patients have 1+ discharges, randomly select one.

Exclude discharges in December 2016 (due to inability to capture 30days)

**2016 Master Beneficiary Summary Annual File (MBSF)**

- AGE >=66 in 2016
- Create look-back and look-forward periods for each beneficiary relative to their Discharge date 🡪 12 months BEFORE Discharge Date (DISCHRDR) and 1 month AFTER
- Parts A, B:

1. = Not entitled; 1 = Part A only; 2 = Part B only; **3 = Part A and Part B**;

A = Part A state buy-in; B = Part B state buy-in; **C = Part A and Part B state buy-in**

- need to look at the individual months relative to Discharge Dates

BUYIN01-BUYIN12 (A and B, values of 3 or C)

- Part D (PTDCNTRCT01-PTDCNTRCT12 = E,H,R,S,X)
- **Create indicator** to Exclude beneficiary if any HMO during the two look-back periods (HMOIND01-HMOIND12) 🡪 all fields in look back period must = 0
- Exclude beneficiaries with use of hospice services in 1 year look-back or 1 month look-forward.

Keep the following variables in MBSF

- BENE_ID DEATH_DT, SEX, RACE, RTI_RACE_CD, DUAL_MO, BUYIN_MO, STATE_CD, CNT_CD, ZIP_CD

***Hypothesis 1A:*** The incidence of these adverse events within 30 days of discharge will be higher among patients with an opioid claim in the week after discharge compared to similar patients without such a claim.

Using cohort derived above, create comparator groups using Medicare Part D.

**Comparator Groups:**

*Opioid Exposed:*

- Identify beneficiaries with a claim for an opioid analgesic within 7 days of hospital discharge

*Non-Opioid Exposed Comparator Group:* To determine the risk of an adverse event attributable to opioids (Hypothesis 1A only), we will include a comparator group of patients hospitalized during the same time frame, matched on their propensity to have received opioids, *but who* *instead had a claim for* *non-opioid analgesics within 7 days of discharge*, including non-steroidal anti-inflammatory drugs (NSAID) and cyclooxygenase-2 inhibitors (COX2).

- Identify beneficiaries with a claim for an NSAID or COX2

**Outcomes:** Adverse events in our analysis, described in Table 2, will be ascertained from the date of the analgesic claim in the 1st week after discharge (unless otherwise specified) through day 30 post-discharge.

| **Table 2. Definition and Operationalization of 30 Day Post-Discharge Adverse Events** | | |
| --- | --- | --- |
| **Adverse Event** | **Definition/Ascertainment** | **CMS Data File(s)** |
| Death | Death from any cause | Master Beneficiary Summary |
| Opioid Overdose | Any *ICD* code for prescription opioid overdose* | MEDPAR, Carrier, Outpatient |
| Healthcare utilization |  |  |
| Emergency department visit | Any claim for emergency department services | MEDPAR, Outpatient |
| Acute hospitalization | Any claim for an acute hospitalization | MEDPAR |
| Clinical symptoms/conditions |  |  |
| Fall | Any *ICD* code for ‘fall’* | MEDPAR, Carrier, Outpatient |
| Delirium | Any *ICD* code for ‘delirium’* | MEDPAR, Carrier, Outpatient |
| Nausea | Any *ICD* code for ‘nausea’* or claim for antiemetics > 7 days after discharge | MEDPAR, Carrier, Outpatient, PDE |
| Constipation | Any *ICD* code for ‘constipation’* or claim for bowel medications > 7 days after discharge | MEDPAR, Carrier, Outpatient, PDE |
| Acute renal failure | Any *ICD* code for acute renal failure | MEDPAR, Carrier, Outpatient |
| Upper GI complications | Any *ICD* code for upper GI complications | MEDPAR, Carrier, Outpatient |
| *The United States transitioned to the ICD-10 system on October 1, 2015. Depending on the most recent years of data available at the time of the request, our analyses may include ICD-9-CM and ICD-10-CM. | | |

***Hypothesis 1B, 1C:***

Hypothesis 1B: We will identify several significant independent patient- and prescribing-related risk factors for post-discharge adverse events among patients with an opioid claim in the week after discharge.

Hypothesis 1C: Among patients with opioid use *prior to hospitalization,* distinct risk factors for post-discharge adverse events will exist, including dosage escalations and overlapping prescriptions.

*Candidate Patient- and Prescribing-Related Risk Factors:* Candidate risk factors (Table 3), were chosen based on hypothesized associations with opioid-related adverse events, based on clinical grounds or prior literature from the outpatient setting, where indicated. *Prescribing-related factors* refer to features of the opioid prescription issued within 7 days of discharge, while *patient-related factors* describe all other characteristics (including prior use of opioid medications and use of non-opioid medications).

| **Table 3. Candidate Patient- and Prescribing-Related Factors Contributing to Post-Discharge Adverse Events** | |
| --- | --- |
| **Patient-Related Factors** | |
| Demographics | Medicare Severity - Diagnosis Related Group (MS-DRG)[^1^](#_ENREF_1)^,^[^52^](#_ENREF_52) |
| Age in 5 year increments[^4^](#_ENREF_4) | Major procedures during hospitalization^†^[^53^](#_ENREF_53) |
| Sex[^4^](#_ENREF_4), Race, Ethnicity | Pain-related diagnoses during hospitalization^†^[^14^](#_ENREF_14)^,^[^53^](#_ENREF_53) |
| Original reason for entitlement (age, disability, renal failure) | Non-specific chest pain |
| State-Buy In coverage (proxy for Medicaid) | Abdominal pain |
| Comorbidity burden/Severity of illness proxies | Pancreatic disorders |
| Elixhauser comorbidities[^4^](#_ENREF_4)^,^[^54-58^](#_ENREF_54) | Headache |
| Medicare chronic conditions (including chronic pain, depression, anxiety, bipolar disorder, and post-traumatic stress disorder | Musculoskeletal back problems |
|  | Calculus of urinary tract |
| Number of secondary discharge diagnoses*[^1^](#_ENREF_1)^,^[^58^](#_ENREF_58) | Surgical versus medical hospitalization |
| Length of hospitalization* | Home healthcare claims, SNF claims in 30d post-discharge |
| ICU stay during hospitalization | Medication use |
| Number of hospitalizations in prior 6 months* | Opioid therapy in 90d prior to admission[^56^](#_ENREF_56)^,^[^57^](#_ENREF_57) |
| Frailty measures^🞎^[^27^](#_ENREF_27)^,^[^28^](#_ENREF_28) | # medication claims in 60d prior to admission* |
| Proxies for history of substance abuse/misuse[^59^](#_ENREF_59) | # medication claims within 30d of discharge* |
| Opioid prescription with oral morphine equivalent (OME) dose of more than 120 mg/day in prior year | # new medication claims within 7d of discharge* |
|  | # claims for other sedating medications within 30d of discharge*[^55^](#_ENREF_55)^,^[^60^](#_ENREF_60) |
| Opioid prescription fills from ≥4 prescribers in prior year | Naloxone claim in 90 days prior or within 7d of discharge |
| Non-fatal prescription opioid overdose in prior year | Claim for bowel medications within 7d of discharge |
| **Prescribing-Related Factors** | |
| Specific opioid (morphine, hydromorphone, oxycodone, oxymorphone, hydrocodone, fentanyl, methadone) | Prescription origin (written, phone, electronic, fax) |
|  | *Variables that will only be included in model focusing on patients with opioid therapy in prior 90 days (Hypothesis 1C):* |
| Short-acting versus long-acting[^55^](#_ENREF_55) |  |
| Dosage in OME*[^55^](#_ENREF_55)^,^[^61-64^](#_ENREF_61) | *Opioid claim differs from preadmission opioid therapy in either type or dose* |
| Total OME*^±^ |  |
| Average daily dose in OME*^Δ^ | *Overlapping prescriptions*^Ф^ |
| Duration/number of pills prescribed |  |
| * Continuous predictor | |
| ^^ We will assess the Davidoff and Faurot frailty measures in terms of both predictive ability and impact on other variables in the models | |
| † We will group procedures and pain-related diagnoses using the Agency for Healthcare Research and Quality Clinical Classification Software – a diagnosis and procedure categorization scheme used in many prior analyses[^14^](#_ENREF_14)^,^[^53^](#_ENREF_53) | |
| ± Total OME will be calculated as dose in OME x quantity dispensed (standard fields in Medicare Part D) | |
| Δ Average daily dose in OME will be calculated as total OME / days supply (standard field in Medicare Part D) | |
| Ф Overlapping prescription will be defined as presence of an opioid claim in the 90 days prior to admission which, based on days supply, would not be expected to have been fully consumed by the time of admission | |
